# Identification of Unknown Substances in Ambient Air (PM_10_), Profiles and Differences between Rural, Urban and Industrial Areas

**DOI:** 10.3390/toxics10050220

**Published:** 2022-04-27

**Authors:** Antonio López, Esther Fuentes, Vicent Yusà, María Ibáñez, Clara Coscollà

**Affiliations:** 1 Foundation for the Promotion of Health and Biomedical Research in the Valencian Region, FISABIO-Public Health, 21, Avenida Catalunya, 46020 Valencia, Spain; lopez_anttob@gva.es (A.L.); fuentes_estfer@gva.es (E.F.); vicent.yusa@fisabio.es (V.Y.); 2 Environmental and Public Health Analytical Chemistry, Research Institute for Pesticides and Water, University Jaume I, S/N, Avenida Sos Baynat, 12071 Castelló de la Plana, Spain; ibanezm@uji.es; 3 Public Health Laboratory of Valencia, 21, Avenida Catalunya, 46020 Valencia, Spain; 4 Analytical Chemistry Department, University of Valencia, Edifici Jeroni Muñoz, Dr. Moliner 50, 46100 Burjassot, Spain

**Keywords:** PM_10_, HRMS, unknown analysis, confidence level, pollutants profiles

## Abstract

A fast and automated strategy has been developed for identifying unknown substances in the atmosphere (concretely, in the particulate matter, PM_10_) using LC-HRMS (MS^3^). A total of 15 samples were collected in three different areas (rural, urban and industrial). A sampling flow rate of 30 m^3^ h^−1^ was applied for 24 h, sampling a total volume of around 720 m^3^. A total of 49 compounds were tentatively identified using very restrictive criteria regarding exact mass, retention time, isotopic profile and both MS^2^ and MS^3^ spectra. Pesticides, pharmaceutical active compounds, drugs, plasticizers and metabolites were the most identified compounds. To verify whether the developed methodology was suitable, 11 substances were checked with their analytical standards and all of them were confirmed. Different profiles for industrial, rural and urban areas were examined. The Principal Component Analysis (PCA) model allowed us to separate the obtained data of the three assessed area. When the profiles obtained in the three evaluated areas were compared using a Volcano plot (the rural area was taken as reference), 11 compounds were confirmed as being discriminant: three of them (3-hydroxy-2-methylpyridine, 3-methyladenine and nicotine) were more likely to be found in industrial sites; ten compounds (3-hydroxy-2-methylpyridine, 3-methyladenine, azoxystrobin, cocaine, cotinine, ethoprophos, imidacloprid, metalaxyl-M, nicotine and pyrimethanil) were more probable in the case of urban sites; finally, triisopropanolamine was more likely to be detected in rural locations.

## 1. Introduction

The atmosphere is a reservoir of volatile emissions mainly coming from anthropogenic sources such as industrial processes, agricultural production or incineration of fossil fuels [1] but also from biogenic sources [2]. Furthermore, the atmosphere is a transport medium where chemical emissions might have lasting effects on the environment and a major impact on human health, even in communities that are somewhat remote from origin sources [1,3]. However, on top of that, the atmosphere is a chemical reactor where compounds continuously transform [4].

The literature provides comprehensive descriptions of the sources, emissions and levels of organic pollutants in the ambient air such as polyaromatic hydrocarbons (PAHs), pesticides, dioxins (PCDD/F), polychlorinated biphenyls (PCBs) and polybrominated diphenyl ethers (PBDEs) [5,6,7,8]. High concentrations of these organic air pollutants increase the risk of mortality, mainly due to respiratory and cardiovascular problems [9,10,11]. In the atmosphere, these airborne pollutants are mainly distributed between two different phases (particulate and gaseous phase) according to their physicochemical properties and ambient conditions (temperature and humidity) [12,13,14]. Particulate matter has raised significant concern in relation to public health, given that 80% or more will deposit somewhere in the respiratory system (often up to 50% of the average mass concentration) [15,16].

Concerning the levels of the organic pollutants in the ambient air, there are certain differences in the atmosphere between urban, industrial and rural areas [17,18]. On the one hand, on the whole, urban areas represent the main areas of anthropogenic emissions [19,20] although in China, black carbon emissions could be larger in rural than in urban areas [21]. In addition, many industrial areas exceed anthropogenic emissions as their activity is a major source of pollutant release into the European environment [22]. Meanwhile, pesticide concentrations are higher in rural than in urban and industrial areas, but other persistent organic pollutants (POPs), such as PCDD/F and PCBs, are found at low concentrations in rural areas [23,24]. In addition, there are differences between these pollutants taking into account the seasons of the year [25].

In order to identify new compounds, it is crucial to carry out an appropriate structural elucidation. In this sense, hybrid instruments have been successfully employed for this purpose in different matrices [26,27,28,29]. These instruments help to provide information on the accurate mass (full scan) and the fragmentation pattern (MS^2^, second-generation spectrum) to carry out a tentative identification of the unknown compound. Furthermore, the emergence of the Orbitrap-tribrid mass spectrometers (which include a quadrupole, an Orbitrap analyser and a linear ion-trap) has provided an increased mass resolving power (450,000 FWHM at *m*/*z* 200) and the scan speed (30 Hz) as well as the possibility of performing MSn for better structural elucidation [30,31]. The suitable characterization of unknown compounds relays on the acquisition of full-scan data, fragmentation data (MS^2^, MS^3^, third-generation spectrum, MS^n^) and their in-depth interpretation. Additionally, and trying to automate structural elucidation, new acquisition tools have been developed for background removal and exhaustive precursor selection [32]. Likewise, an automated library search has been improved through the data processing tool Compound Discoverer TM (CD) [33].

In the literature, there are few studies related to non-target analysis in ambient air using liquid chromatographic techniques coupled to high-resolution mass spectrometry [34,35,36,37,38], but in some cases, only MS^2^ fragmentation data were employed. The other published studies in the literature employed gas chromatography [39,40,41].

In the present study, a comprehensive strategy to identify unknown pollutants in the particulate matter of ambient air (PM_10_) and to characterize the pollutants profiles in different areas (industrial, urban and rural) has been developed. This methodology enables us to detect compounds in the ambient air using MS^2^ and MS^3^ spectra. The capabilities of a Tribrid instrument (MS^3^ acquisition data) improve the structural elucidation obtained in other studies (MS^2^ acquisition). Furthermore, the developed methodology is able to detect which compounds have the greatest power of discrimination between areas, obtaining 11 characteristic substances in the three assessed areas. For this purpose, the improved capabilities of a Tribrid instrument for non-target acquisition data (MS^3^) has been combined with the powerful automated data processing workflows including PCA analysis and Volcano plot.

Eleven compounds that allowed discriminating between these three areas were finally identified and confirmed using analytical reference standards. As far as we are aware, this study is the first work that uses Tribrid mass spectrometer to search for unknown compounds and to characterize pollutants profiles in the ambient air.

## 2. Materials and Methods

### 2.1. Reagent and Chemicals

The following certified commercial standards were employed to create a Quality Control Reference Material (QC_RM_): acetaminophen, caffeine, reserpine, sulfaguanidine, sulfadimethoxine, terfenadine and Val-Tyr-Val, all purchased from Sigma Aldrich (Taufkirchen, Germany). Individual stock standards were prepared weighting 5 mg of pure standard using a 5-decimal analytical balance and dissolving each compound in 25 mL of acetone. Mix working solutions at 10 and 1 mg L^−1^ were prepared in H_2_O/MeOH (70:30, *v*:*v*) and stored in capped amber vials at −20 °C. These standards were added in the evaluated samples at 10 ng mL^−1^.

In addition, to confirm the substances detected in the different areas studied, the following commercial standards were prepared: 3-hydroxy-2-methylpyridine, 3-methyladenine, azoxystrobin, cocaine, cotinine, ethoprophos, imidacloprid, metalaxyl, nicotine, pyrimethanil and triisopropanolamine, all provided by Sigma Aldrich (Taufkirchen, Germany). Individual analytical standards were prepared by weighting 10 mg of pure standard employing a 5-decimal analytical balance and dissolving each compound in 50 mL of acetone. Mix working solutions at two different concentrations (1000 ng mL^−1^ and 50 ng mL^−1^) were prepared in H_2_O/MeOH (70:30, *v*:*v*) and were stored at −20 °C using capped amber vials.

Acetone, ethyl acetate and water were of HPLC grade and were purchased from Merck (Darmstadt, Germany). Methanol was HPLC grade and was supplied by Scharlau (Barcelona, Spain). Formic acid 98% was provided by Panreac (Barcelona, Spain).

### 2.2. Sampling and Site Characterization

Sample collection was carried out in the same way as described by López et al. (2017) [6]. Samples were collected using a high-volume sampler from Digitel (Volketswill, Germany) and quartz fiber filters (QFF) of 150 mm of diameter, supplied by Munktell Filter AB (Falun, Sweden). A sampling flow rate of 30 m^3^ h^−1^ was applied for 24 h, sampling a total volume of around 720 m^3^. PM_10_ samples were collected in May 2021 from three different areas: one industrial (Onda), one urban (Valencia-Viveros), and one rural (Burriana) (see Table 1). A total of 15 samples (5 samples in each area) were collected.

### 2.3. Sample Preparation

When applying a wide-scope analysis, it is preferable to use a non-selective sample preparation to extract the highest number of compounds. A generic extraction method developed in a previous study using microwave assisted extraction (MAE) with ethyl acetate was employed [34]. In short, extraction was carried out using a Mars system from CEM Corporation (Mathews, NC, USA) equipped with Teflon^®^ TFM 100 mL extraction vessels. The extraction conditions were as follows: a temperature of 50 °C was applied for 20 min, using 1200 W, and 30 mL of ethyl acetate was added. After cooling, the reactor was opened, and the extracts were filtered. Samples were evaporated with a Turbo Vap 500 (Zymark, Idstein, Germany), and the extracts were then re-dissolved with 1 mL of QCRM and centrifuged prior to the LC–HRMS determination. The pooled sample was obtained by mixing an aliquot of each extracted sample.

### 2.4. LC-HRMS Analysis

The chromatographic separations were performed on a Thermo Ultimate 3000 UHPLC (Ultra High Performance Liquid Chromatography) system equipped with a UHPLC reversed-phase column Hypersil Gold, 1.9 μm particle size, 100 mm × 2.1 mm (Thermo Scientific) interfaced with an Orbitrap ID-X Tribrid mass spectrometer (Thermo Fisher Scientific, Bremen, Germany). The injection volume was 5 μL, and the column was eluted with a flow rate of 300 μL min^−1^. The initial conditions of the mobile phase were: 90% phase A (water + 0.1% formic acid) and 10% B (methanol + 0.1% formic acid). The employed gradient was from 0 to 18 min., linear gradient to 70% B; from 18 to 21.5 min., linear gradient to 98% B; maintained at 98% B from 21.5 to 25 min; linear decrease to initial conditions from 25 to 26 min, and maintained until 30 min.

The mass spectrometer used was a heated electrospray interface (H-ESI) operating in positive ionization. The H-ESI parameters were the following: electrospray voltage of 3.5 kV; sheath gas of 25 arbitrary units (a.u); and auxiliary gas of 5 a.u. The ion transfer tube operated at 270 °C and the vaporizer temperature at 180 °C. The full scan (FS) was acquired with a resolving power of 120,000 FWHM for parental ions and a mass range of 100–900 *m/z*. The MS^2^ data were acquired at a resolving power of 15,000 FWHM, with a precursor mass range of 125–900 *m/z*. Both FS and MS^2^ were acquired in the Orbitrap analyser. The MS^3^ precursor was isolated in the quadrupole mass filter (0.4 Da), fragmented in the HCD cell, and detected in the Ion Trap (FWHM ≤ 0.3) [29].

### 2.5. Acquisition Workflow for Unknown Analysis

A wide range of different acquisition workflows can be achieved by the use of Orbitrap Tribrid-HRMS. In this study, the AcquireX© Data Acquisition Technology (Thermo Fisher Scientific) was used in Deep Scan (DS) mode, which automatically creates an exclusion and inclusion lists after the injection of a blank and a sample (acquired in full scan), respectively. Three iterative injections of the air samples automatically update both lists (inclusion/exclusion) which permits an in-depth acquisition of the relevant features, excluding those present in the blank [29,42].

### 2.6. Data Processing

To be able to tentatively identify the chemicals, post-acquisition data processing was performed. Raw files were processed by CD ^TM^ 3.1 (Thermo Fisher Scientific). Mass Frontier ^TM^ 8.1 has been used in order to confirm MS^3^ spectra. Figure S1 shows the general workflow employed in the Orbitrap-tribrid system, and Table S1 provides a description of each node and Figure S2 illustrates the CD workflow.

In addition, CD has been employed for the statistical analysis. The data were analysed by PCA to compare the differences between the studied areas. PCA is the most commonly used approach to reduce the number of variables of a dataset while preserving as much information as possible [43]. PCA enables to explore differences between samples collected in different areas.

Moreover, a Volcano plot was performed to observe the more relevant substances in each area. A volcano plot is a type of scatter plot for replicate data that shows statistical significance (*p*-value) versus magnitude of change (log_2_Fold measures how much a variable has changed between the two measurements). In this plot, the x axis represents the log_2_Fold between two samples groups (generated ratio), and the y axis represents the negative log_10_ of the *p*-value (test of significance) of the fold change. Compounds in the volcano plot showing a relatively low-fold change between the two different evaluated areas appear in the centre of the plot (few differences between the two compared areas) while compounds that have significant differences between the compared areas are found in the upper-left (more likely to be found in industrial/urban areas) or the upper-right (more likely in rural areas) [44]. The following parameters were employed in order to construct the Volcano plot: *p*-value = 0.001 and log_2_Fold = 3.

### 2.7. Identification Criteria

In order to identify substances in the analysed samples, different levels of substance identification were considered following the criteria described by Yusà et al., (2020) [29].

The criteria used for the different levels of identification confidence were the following: predicted composition, exact mass, isotope pattern, MS^2^ and MS^3^ spectra match and RT (min). Taking into account these parameters, five different levels were defined (see Table 2) depending on the compliance of the identified substances with the identification criteria. The substances identified at Level 1 in the different Volcano plots have been compared with analytical reference standards, when these were available. In a second injection, concentrations in the ambient air (pg m^−3^) for these substances were estimated using the response of their analytical standards. The resulting one-point calibration (corresponding to 66 pg m^−3^) on identified compounds yielded semi-quantitative concentrations. This approach gives only approximate concentrations, because the purpose of the study is basically to identify and if possible confirm substances but not an accurate quantification method.

For the other 38 substances identified at Level 1, concentrations in the atmosphere (pg m^−3^) were estimated taking into account the average response factor of the seven substances employed in the QC_RM_ (see average factor in Table S2).

### 2.8. Quality Assurance/Quality Control

To show the robustness of the developed methodology, some aspects have been assessed.

(i) Instrument drift was monitored by analysing the areas of QC_RM_ in the batch. Coefficient of variation (CV (%)) should be lower than 30%.
(ii) To assure the unbiased of the methodology, all samples were injected in the same batch, sample injections were randomized, and sample preparation was performed the same day [45].
(iii) Blank samples results were checked in order to assure that the exclusion list acquired did not contain any contamination.
(iv) The batch acquired in CD was checked to assure that the seven substances added in the QC_RM_ were identified.

## 3. Results and Discussion

**Figure 1:**
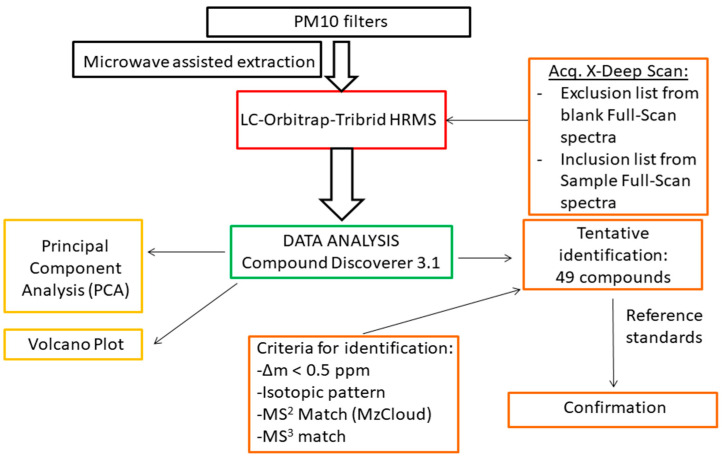
Workflow of the different steps of the study.

The fifteen samples (5 from each area) were analysed according to the workflow described in Figure 1. After the instrumental analysis, the acquired analytical data (raw data) were processed for the following: (i) identification of unknown substances and (ii) multivariate analysis (PCA) and Volcano plot.

### 3.1. Identification of Unknown Substances

Using the employed workflow, a total of 2528 features were automatically annotated (see Figure S2). These features were classified into five different levels taking into account the identification criteria (see Table 2): 49 features met the criteria to be considered the highest confidence level (Level 1), 52 features were identified with a high confidence level (Level 2) and 1011 features were identified in level 3; meanwhile, 920 substances were tentatively identified in level 4 and 496 features were tentatively identified with the lowest confidence level (Level 5).

The experimental retention time (RT) was compared with the predicted RT considering log K_ow_ [46] for each substance, using a known set of 116 analytical reference standards (see Table S3 and Figure S3). In most cases (more than 80%), the difference between the experimental retention time for the tentatively identified compounds and the predicted retention time was lower than 2 min.

Moreover, given that MS^3^ has been employed in our study and CD only automatically compares the acquired MS^2^ spectra with those included in the database (MzCloud) to perform the tentative identification, MS^3^ data need to be manually checked to increase the confidence of our results [47]. Ten out of the 49 identified substances did not have available MS^3^ acquired spectra in CD. Consequently, the experimental spectrum of the other 39 identified substances was compared with the available MS^3^ spectrum in MzCloud. For those compounds that did not have MS^3^ spectra in MzCloud, the theoretical in silico fragmentation performed with CD was compared with the experimental spectra. A total of 27 out of the 39 substances were successfully identified at the MS^3^ level.

Table S4 shows the 49 identified substances at the highest level of confidence (Level 1), which belong to different types of compounds such as pesticides, pharmaceutical active compounds, drugs, plasticizers or metabolites. While some of them had been previously detected in the ambient air, there were others which, as far as we know, have not been previously detected in the ambient air. In order to confirm that the developed methodology is suitable to identify unknown compounds, the discriminant substances detected in Section 3.2 (3-methyladenine, 3-hydroxy-2-methylpyridine, azoxystrobin, cocaine, cotinine, ethoprophos, imidacloprid, metalaxyl-M, nicotine, pyrimethanil and triisopropanolamine) were checked with their analytical reference standards. All of them were confirmed.

### 3.2. Differences between Areas

The acquisition in full-scan mode allowed us to obtain an individual footprint for each evaluated sample [48,49].

**Figure 2:**
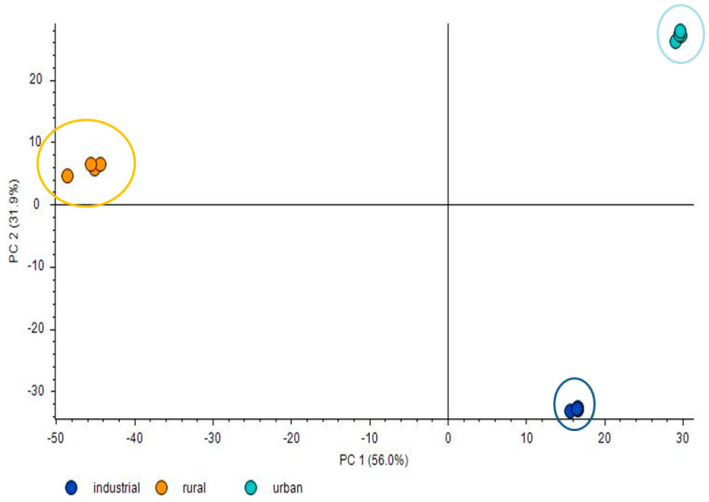
Two dimensional PCA score plot of air samples for industrial, rural and urban areas.

A PCA of the analysed samples was carried out. Figure 2 shows that the PCA model clearly enabled air samples collected in the different sampling sites to be separated. These differences imply different pollutant profiles for the assessed areas (industrial, urban and rural).

**Figure 3:**
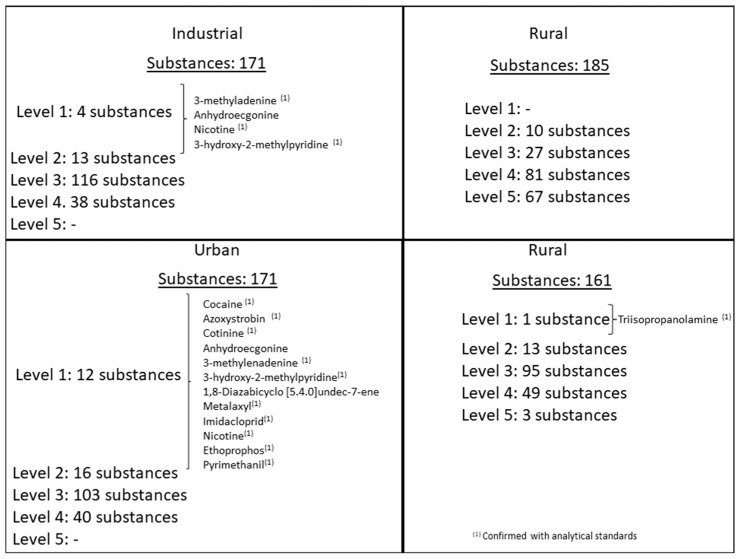
Obtained results in the Volcano plots (industrial vs. rural and urban vs. rural).

In a second step, the differences between each area (industrial, rural and urban) were assessed by comparing them in pairs to determine which substances have the greatest power of discrimination among the studied areas. In both cases, rural samples were used as reference. The substances with the greatest power of discrimination were classified according to the confidence level of their identification (see Table 2). Substances classified as Level 1 were compared with an analytical reference standard, when available so as to confirm their identification. Figure 3 summarizes the results obtained in the different Volcano plots.

Thus, taking into account the results obtained in the volcano plot where industrial samples versus rural samples were compared (see Figure S4), 171 substances (located in the upper-left) were more likely to be present in industrial areas (Table S5) while 185 compounds (located in the upper-right) were more likely to be found in rural areas (Table S6).

**Figure 4:**
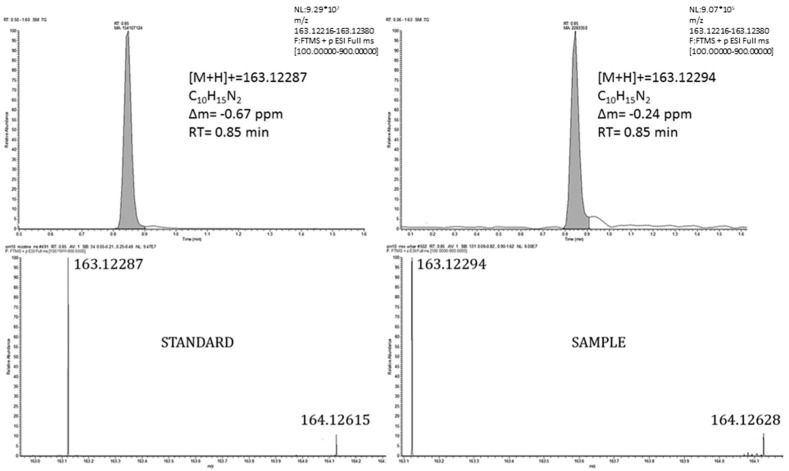
Extracted ion chromatogram (**top**) and isotopic profile (**bottom**) corresponding to the protonated molecule of nicotine in a standard (**left**) and a real sample (**right**).

Four out of the 171 substances more likely to be present in industrial areas were classified as Level 1. Three of them were confirmed with their analytical standard (3-methyladenine, 3-hydroxy-2-methylpyridine and nicotine), and one of them (anhydroecgonine) was not possible to confirm because the analytical standard was unavailable. Nicotine was previously detected in the ambient air of certain urban and industrial areas: the urban atmosphere of Algiers [50]; industrial and urban areas in the National Territory Capital of Delhi in India [51] and the urban atmosphere of cities such as Amsterdam, London and Stockholm [52]. 3-methyladenine is an inhibitor of phosphatidylinositol 3-kinases (PI3K) and also has a role as a human metabolite [53], and 3-hydroxy-2-methylpyridine is an intermediate in the vitamin B6 metabolism [54]. To our knowledge, neither of these two substances has been previously identified in the ambient air. Figure 4 illustrates the chromatographic confirmation for nicotine, while in Figure S5 and Figure S6, the chromatographic confirmations for 3-methyladenine and 3-hydroxy-2-methylpyridine are shown.

Regarding the compounds more likely to be detected in rural areas, none of those detected compounds were identified at the highest confidence level (Level 1).

In the volcano plot obtained between urban and rural samples (see Figure S7), 171 compounds (in the upper-left) were classified as being more probable detected in urban areas (see Table S7), and 161 compounds (in the upper-right) were classified as more likely in rural areas (Table S8).

Twelve out of the 171 compounds classified as more probable in urban areas were identified with the highest level of confidence (Level 1). Ten out of twelve substances were confirmed using analytical standards: 3-hydroxy-2-methylpyridine, 3-methyladenine, azoxystrobin, cocaine, cotinine, ethoprophos, imidacloprid, metalaxyl, nicotine and pyrimethanil. However, two other compounds, (anhydroecgonine and 1,8-diazabicyclo [5.4.0] undec-7-ene) could not be confirmed because the analytical standards were unavailable.

**Figure 5:**
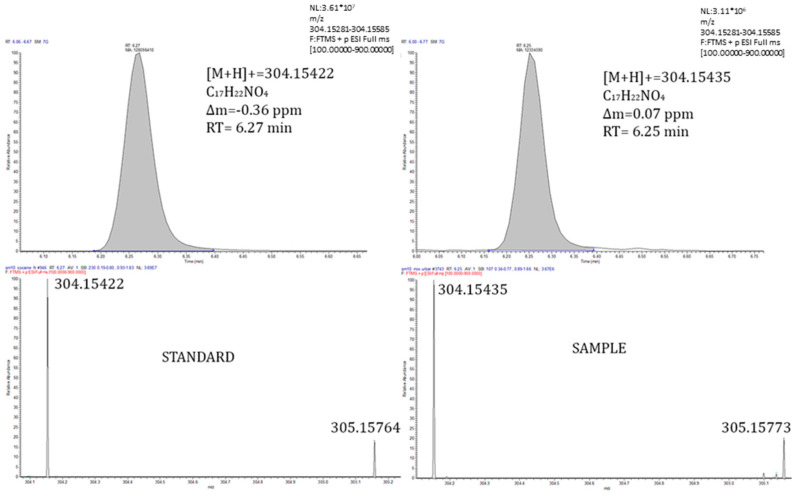
Extracted ion chromatogram (**top**) and isotopic profile (**bottom**) corresponding to the protonated molecule of cocaine in a standard (**left**) and a real sample (**right**).

Cocaine was also detected in the atmosphere of certain major cities such as Amsterdam, London [52] and Barcelona [55]. Cotinine, the most important metabolite of nicotine, was detected in urban samples. Taking into account that nicotine (its parent) has been found in some urban and industrial areas around the world, [50,51,52], it is likely that cotinine is present in urban areas. Figure 4 (nicotine), Figure 5 (cocaine) and Figure S8 (cotinine) show the confirmation of these compounds.

In this study, certain pesticides such as azoxystrobin, ethoprophos, imidacloprid, metalaxyl and pyrimethanil were identified and confirmed. These pesticides have been already detected in our region in previous studies [6]. Moreover, pyrimethanil was detected in the same urban area studied in an earlier study carried out by our research group. The detected semi-quantified levels in the present study are higher (21.84 pg m^−3^ vs. 8.00 pg m^−3^) than the ones found in our previous study [6]. Figures S9–S13 show the chromatographic confirmation of the detected pesticides.

Concerning the compounds that are more likely to be found in rural areas, one of them (triisopropanolamine) was confirmed with their analytical standard (see Figure S14) and then classified as Level 1. Triisopropanolamine is widely used as emulsifier, stabilizer, surfactant and chemical intermediate. One of its main applications of triisopropanolamine is to neutralize pesticides [56].

## 4. Study Limitations

Samples were collected in only one season, and there may be differences in the particulate phase at different seasons of the year.

In some cases, it could be difficult to classify in only one group a sampling area that should be part of more than one group.

Regarding structural elucidation, the detection of isomers is not possible, and we should use other techniques (for instance, nuclear magnetic resonance).

## 5. Conclusions

A novel analytical approach has been developed in order to identify unknown compounds in PM_10_ ambient air. This strategy enables the tentative identification of 49 compounds, belonging to different chemical groups such as pesticides, pharmaceutical products, drugs, plasticizers and metabolites. Furthermore, when applying it, the use of MS^3^ showed better structural elucidation for the detected compounds.

Different profiles were obtained for rural, urban and industrial areas, and since, each area has a specific footprint, different organic pollutants were detected.

When industrial and rural areas were compared, three substances were confirmed in industrial areas: 3-methyladenine, nicotine and 3-hydroxy-2-methylpyridine. In the comparison of urban and rural areas, 10 substances were detected in urban areas: 3-hydroxy-2-methylpyridine, 3-methyladenine, azoxystrobin, cocaine, cotinine, ethoprophos, imidacloprid, metalaxyl, nicotine and pyrimethanil whereas the presence of one substance (triisopropanolamine) was confirmed in rural areas.

This strategy could be implemented as an analytical tool in future studies related to Air Quality projects worldwide. Moreover, it may provide useful information about unknown compounds (regulated or not) which can be detected in the ambient air by LC-HRMS.

## Figures and Tables

**Table 1:** Description of sampling sites of PM_10_ samples.

Sampling Site	Latitude	Longitude	Description
Burriana	39°53′52″	0°03′54″	Rural and agricultural area surrounded by citrus groves (orange trees). Samples collected about 20 m above sea level.
Onda	39°57′46″	0°15′00″	Industrial area. Samples collected about 160 m above sea level.
Valencia-Viveros	39°28′46″	0°22′10″	Commercial and urban area and inside a park (Viveros) with gardens. Samples collected at 11 m above sea level.

**Table 2:** Levels of confidence and criteria used for tentative identification.

Level	Parameter	Criteria
Level 1	Molecular formula (Predicted composition) ^1^	Full match
	∆ mass (ppm) ^2^	<0.5 ppm
	Isotope profile (SFit) ^3^	>70%
	MzCloud Match ^4^	>70%
	MS^2^ Data ^5^	Yes
	RT (min)	Consistent with the predicted RT (min) (±2 min)/analytical standard *
Level 2	Molecular formula (Predicted composition) ^1^	Full match
	∆ mass (ppm) ^2^	<0.5 ppm
	Isotope profile (SFit) ^3^	50–70%
	MzCloud Match ^4^	50–70%
	MS^2^ Data ^5^	Yes
	RT (min)	Consistent with the predicted RT (min) (±2 min)
Level 3	Molecular formula (Predicted composition) ^1^	Full match
	∆ mass (ppm) ^2^	<0.5 ppm
	MS^2^ Data ^5^	Yes
Level 4	Molecular formula (Predicted composition) ^1^	Full match
	∆ mass (ppm) ^2^	<0.5 ppm
	MS^2^ Data ^5^	No
	Predicted substance ^6^	Name
Level 5	Molecular formula (Predicted composition) ^1^	Full match
	∆ mass (ppm) ^2^	<0.5 ppm
	MS^2^ Data ^5^	No
	Predicted substance ^6^	No Name

^1^ Molecular formula (Predicted composition): Assigned elemental composition coincidence with a proposed substance from databases (ChemSpider, Mass List). ^2^ Δ mass (ppm): mass error (in ppm). ^3^ Isotope profile (SFit): match between the experimental isotopic pattern and the theoretical one. ^4^ MzCloud Match: match between the experimental and the MS^2^ spectra in the mzCloud library. ^5^ MS^2^ data: Match between experimental MS^2^ spectra and the theoretical one in CD. ^6^ Predicted substance: Name described in one of the employed databases: mzVault/mzCloud Library/ChemSpider/Mass List. * When analytical standard was available, the RT difference between standard and sample was compared.

## Supplementary Materials

The following supporting information can be downloaded at: https://www.mdpi.com/article/10.3390/toxics10050220/s1. Table S1: Description of the different nodes of the CD workflow; Table S2: Average response factor of internal standard; Table S3: Table of Log K_ow_ vs. Retention time (RT) of the analytical reference standards; Table S4: Identified compounds and spectrometric and chromatographic criteria; Table S5: Compounds more probably present in industrial areas (Industrial-Rural); Table S6: Compounds more probably present in rural areas (Industrial-Rural); Table S7: Compounds more probably present in urban areas (Urban-Rural); Table S8: Compounds more probably present in rural areas (Urban-Rural); Figure S1: Workflow used in AcquireX Deep Scan Mode-MS^3^; Figure S2: Workflow used in Compound Discoverer™ data processing; Figure S3: Graphic of Log K_ow_ vs. Retention time (RT) of the analytical reference standards; Figure S4: Volcano plot for identification of discriminating substances between industrial and rural areas (employed parameters: *p*-value = 0.001 and log_2_Fold = 3); Figure S5: 3-methyladenine; Figure S6: 3-hydroxy-2-methylpyridine; Figure S7: Volcano plot for identification of discriminating substances between urban and rural areas (employed parameters: *p*-value = 0.001 and log_2_Fold = 3); Figure S8: Cotinine; Figure S9: Azoxystrobin; Figure S10: Ethoprophos; Figure S11: Imidacloprid; Figure S12: Metalaxyl; Figure S13: Pyrimethanil; Figure S14: Triisopropanolamine.
