# Supplementary material for: Identification of Unknown Substances in Ambient Air (PM10), Profiles and Differences between Rural, Urban and Industrial Areas
Source: Toxics. 2022 Apr 27;10(5):220. doi: 10.3390/toxics10050220 (PMC9145881; doi:10.3390/toxics10050220)
Supplement: Supplementary file 1 [file toxics-10-00220-s001.zip › toxics-1686634-supplementary.pdf]

Supplementary Materials: Identification of Unknown Substances in Ambient Air (PM_{10}), Profiles and Differences between Rural, Urban and Industrial Areas

Antonio López, Esther Fuentes, Vicent Yusà, María Ibáñez and Clara Coscollà

Table S1. Description of the different nodes of the CD workflow.

NODE	
SELECT SPECTRA	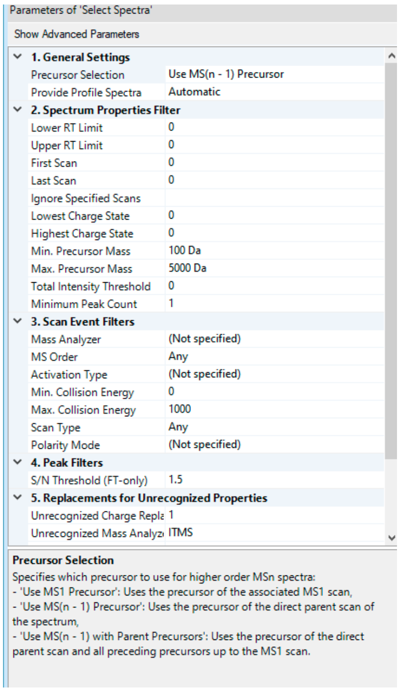
ALIGN RETENTION TIME	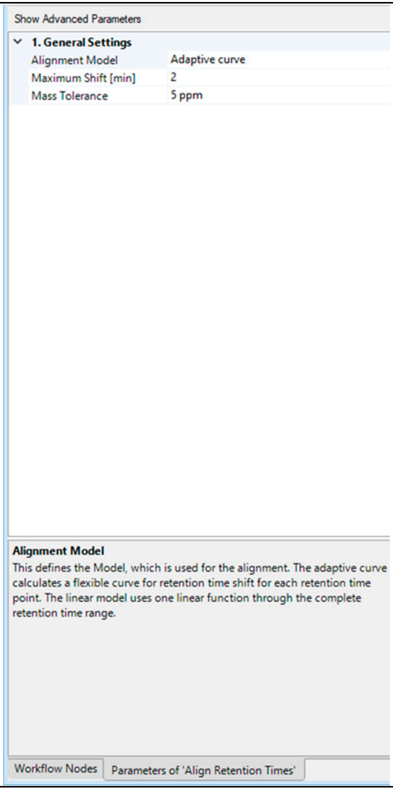

CREATE TRACE PATTERN	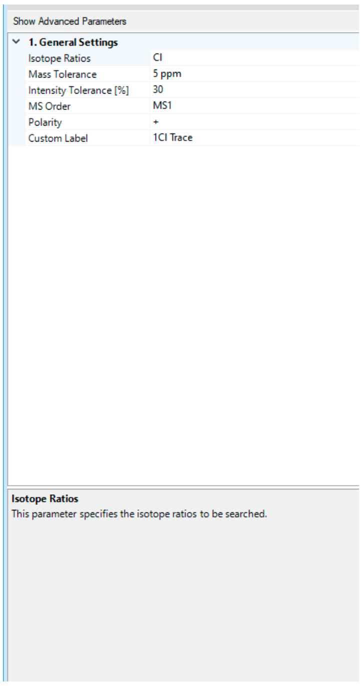
DETECTED COMPOUNDS	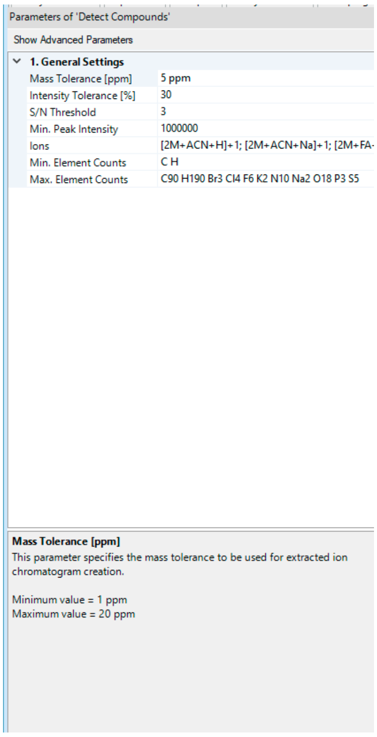

GROUP COMPOUNDS

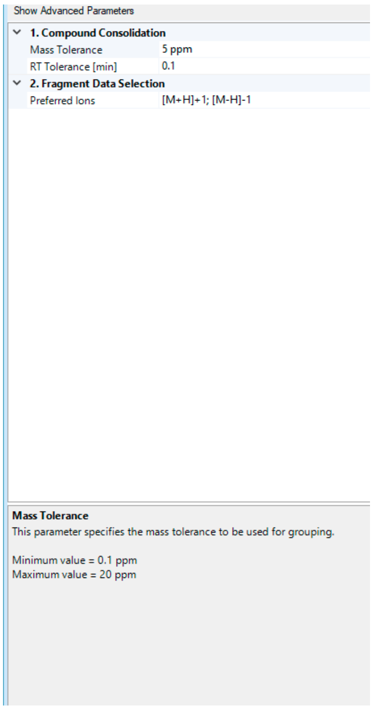

MERGE FEATURES

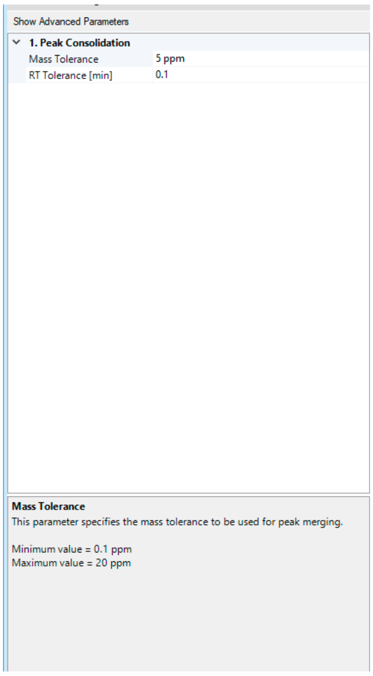

<table>
<tr>
<td>FILL GAPS</td>
<td>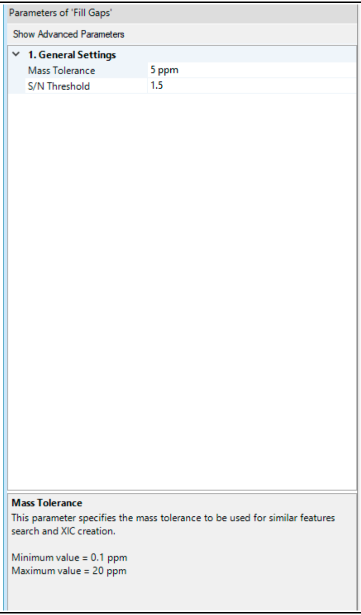
</td>
</tr>
<tr>
<td>NORMALIZED AREAS</td>
<td>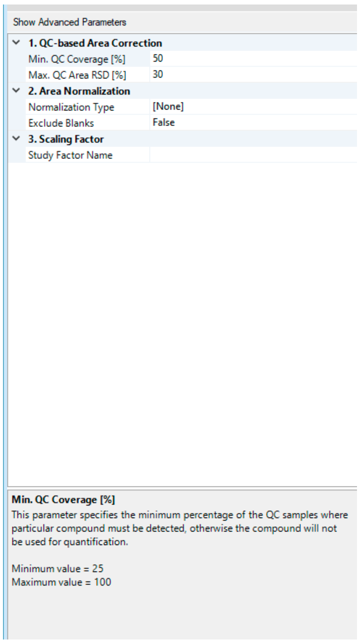
</td>
</tr>
</table>

<table>
<tr>
<td>MARK BACKGROUND COMPOUNDS</td>
<td>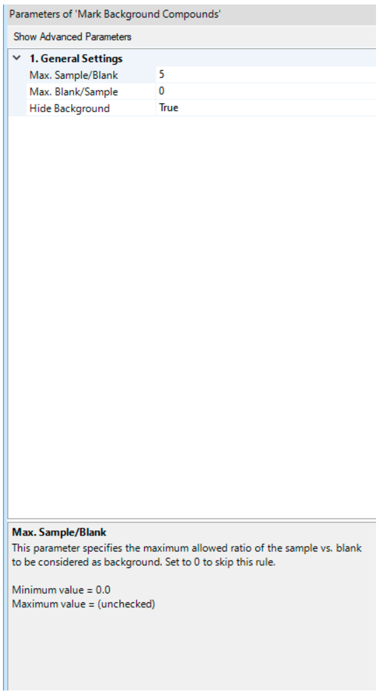
</td>
</tr>
<tr>
<td>CALCULATE MASS DEFECT</td>
<td>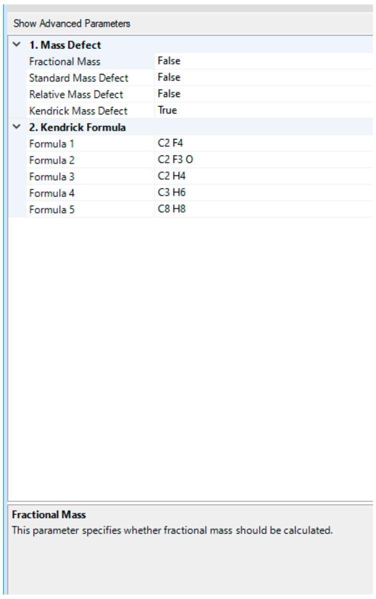
</td>
</tr>
</table>

<table>
<tr><td>SEARCH MzVault</td><td>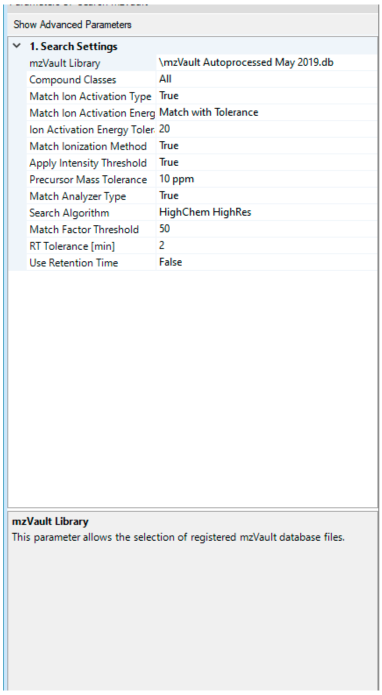
</td></tr>
<tr><td>PATTERN SCORING</td><td>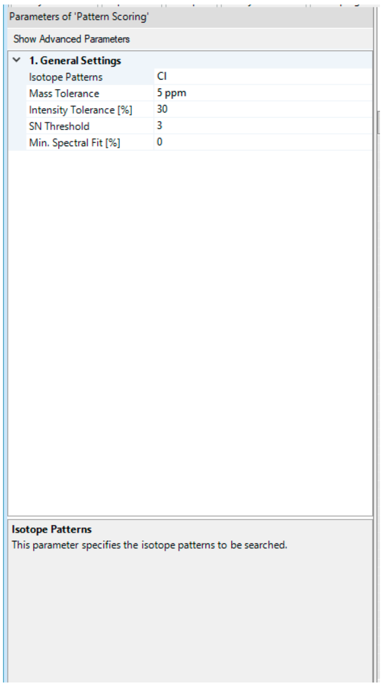
</td></tr>
</table>

SEARCH MzCloud

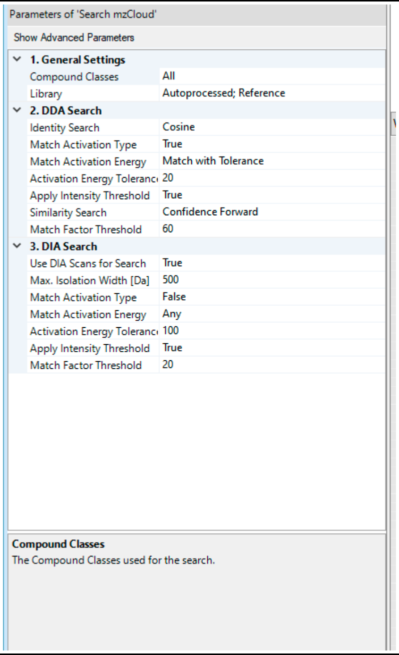

Parameters of 'Search mzCloud'

Show Advanced Parameters

**1. General Settings**	
Compound Classes	All
Library	Autoprocessed; Reference
**2. DDA Search**	
Identity Search	Cosine
Match Activation Type	True
Match Activation Energy	Match with Tolerance
Activation Energy Toleranc	20
Apply Intensity Threshold	True
Similarity Search	Confidence Forward
Match Factor Threshold	60
**3. DIA Search**	
Use DIA Scans for Search	True
Max. Isolation Width [Da]	500
Match Activation Type	False
Match Activation Energy	Any
Activation Energy Toleranc	100
Apply Intensity Threshold	True
Match Factor Threshold	20

Compound Classes
The Compound Classes used for the search.

PREDICT COMPOSITIONS

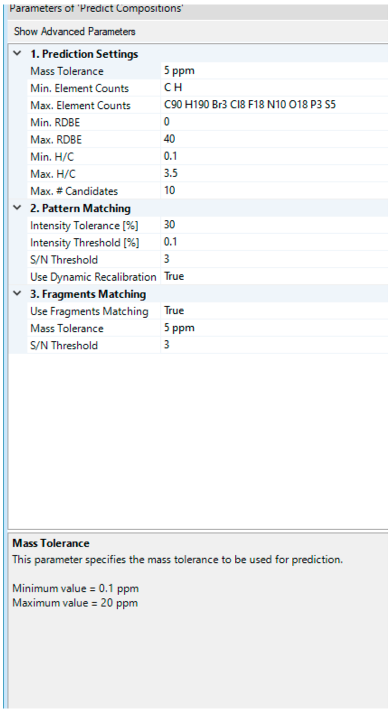

Parameters of 'Predict Compositions'

Show Advanced Parameters

**1. Prediction Settings**	
Mass Tolerance	5 ppm
Min. Element Counts	C H
Max. Element Counts	C90 H190 Br3 Cl8 F18 N10 O18 P3 S5
Min. RDBE	0
Max. RDBE	40
Min. H/C	0.1
Max. H/C	3.5
Max. # Candidates	10
**2. Pattern Matching**	
Intensity Tolerance [%]	30
Intensity Threshold [%]	0.1
S/N Threshold	3
Use Dynamic Recalibration	True
**3. Fragments Matching**	
Use Fragments Matching	True
Mass Tolerance	5 ppm
S/N Threshold	3

Mass Tolerance
This parameter specifies the mass tolerance to be used for prediction.

Minimum value = 0.1 ppm
Maximum value = 20 ppm

ASSIGN COMPOUND ANNOTATIONS	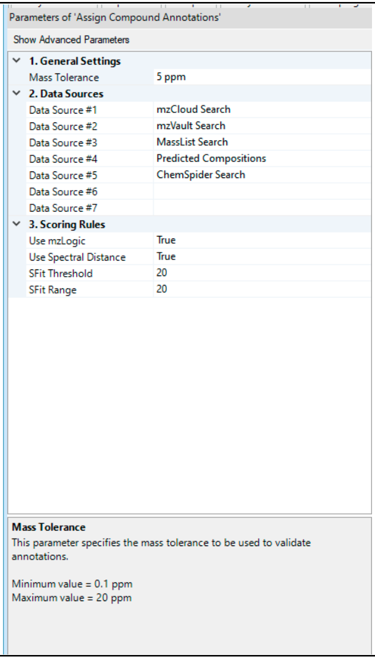
SEARCH MASS LIST	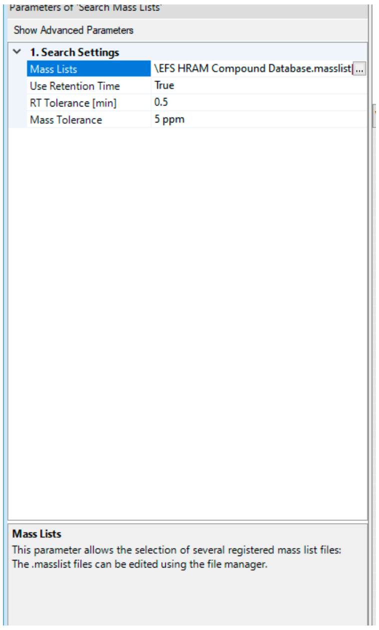

APPLY MzLogic	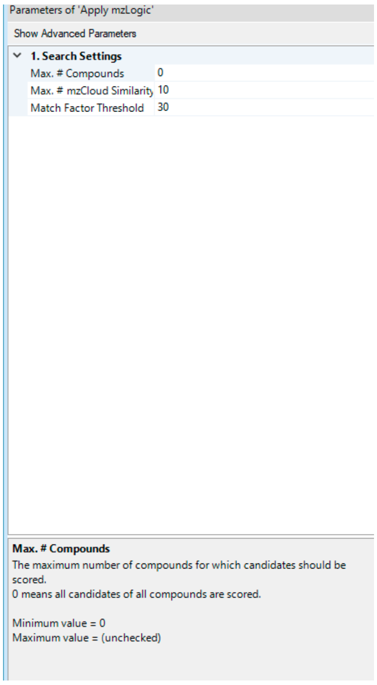
SEARCH ChemSpider	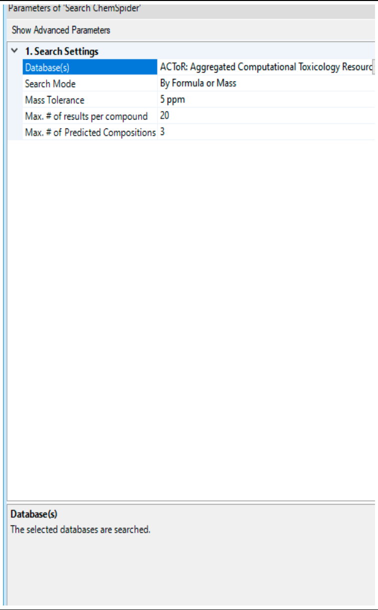

APPLY SPECTRAL DISTANCE

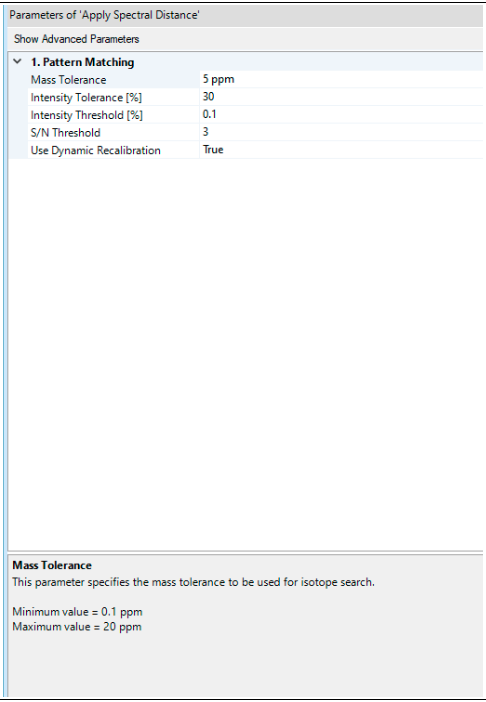

DIFFERENTIAL ANALYSIS

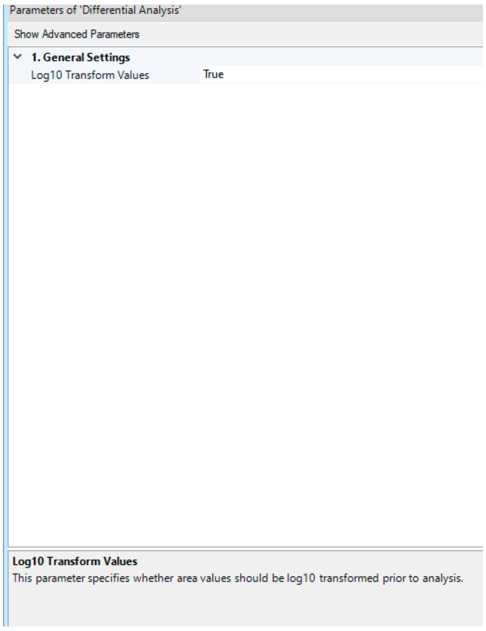

Table S2. Average response factor of internal standards.

Internal standard	Response factor (Peak area mL ng^{-1})
Acetaminophen	435189
Caffeine	263112
Reserpine	354877
Sulfadimethoxine	563987
Sulfaguanidine	145817
Terfenadine	1408605
Val-Tyr-Val	370978
**AVERAGE**	**506081**

Table S3. Table of Log Kow vs Retention time (RT) of the analytical reference standards.

Analite	RT (min)	log Kow (log P)
L-Histidine	0.78	−1.26
Chlormequat	0.79	−3.80
3-hydroxy-2-methylpyridine	0.84	−0.29
Nicotine	0.84	0.72
3-methyladenine	0.80	−0.98
Triisopropanolamine	0.83	−0.07
Sulfaguanidine	0.85	−1.22
L-Pyroglutamic acid	0.94	−2.39
L-Tyrosine	0.97	0.38
Cotinine	1.03	−0.23
Dimethyldithiophosphate	1.20	0.65
Urocanic acid	1.29	0.01
Methamidophos	1.46	−0.82
Acephate	1.84	−0.85
Acetaminophen	1.95	0.46
Omethoate	2.25	0.06
Diethylthiophosphate	2.41	0.68
6-methoaxyquinoline	2.79	2.17
5-hydroxy-thiabendazole	2.86	1.73
Carbendazim	2.90	1.52
Simazine 2-Hydroxy	2.92	−1.22
2-diethylamino-6-methyl pyrimidin-4-ol/one	2.96	1.11
Vancomycin	3.00	−1.44
PEG n5	3.08	−2.59
Caffeine	4.08	−0.07
Desisopropylatrazine	4.43	1.16
Val-Tyr-Val	4.86	0.80
6-Chloronicotinic acid	5.33	0.98
4-nitrophenol	5.77	1.57
acetamiprid-N-desmethyl	6.08	0.65
Cocaine	6.26	2.17
Cefoperazone	6.38	1.43
Salicylic acid	7.19	2.06
Sulfadimethoxine	7.67	1.63
Malathion dicarboxilic acid	7.78	1.48
Pyroquilon	10.00	1.40
Dichlorvos	10.15	0.71
Pyrimethanil	10.34	2.84
Bentazone	10.63	2.80
Trichlorfon (Dylox)	10.90	0.43
Phenytoin	11.08	2.29
Bromoxynil	11.25	2.95
Dibutyl phthalate	11.55	4.82
Diethyl phthalate	11.55	2.70
Pyrene	12.08	5.17
p-Toluenesulfonamide	12.15	0.79
Benodanil	12.20	3.23

3,5,6-Trichloro-2-pyridinol	12.71	2.16
2,4-D	12.77	2.59
Isoproturon	12.86	2.32
N,N-diethyl-m-toluamide/DEET	13.17	1.96
Reserpine	13.19	3.32
MCPA	13.32	2.49
Inabenfide	14.27	2.67
TCMTB	14.29	3.12
Acetochlor mercapturate	14.75	2.86
Alachlor mercapturate	14.75	2.86
N,N'-Dicyclohexylurea	14.79	3.10
Valproic acid	14.89	2.72
Flamprop	15.18	3.47
Mecoprop	15.19	2.84
Hydroxy-tebuconazole	15.42	2.50
3-phenoxybenzoic acid	15.64	3.91
4-Fluoro-3-phenoxybenzoic acid	15.83	4.05
D,L-Camphor	16.14	2.13
Methyl dihydrojasmonate	16.14	2.67
Tepraloxydim	16.28	2.88
Fenhexamid	16.54	4.02
Benzophenone-3 (Oxybenzone)	16.59	3.64
Acetochlor	16.61	2.92
Fluopyram	16.68	4.36
Fenvalerate free acid	16.72	3.33
Triclosan glucuronide	16.72	2.53
Rifaximin	16.85	3.22
Metolachlor	16.89	3.00
Dimethyl sebacate	16.89	2.79
Benzoic acid	16.98	1.89
Prochloraz	17.15	3.80
Fipronil	17.66	4.76
Triclosan sulfate	17.71	4.66
Dinoterb	17.77	3.42
Spinosad A (Spinosyn A)	18.21	4.80
Butocarboxim	18.27	1.49
Chlorfenvinphos	18.43	4.51
3,5-ditert-butyl-4-hydroxybenzaldehyde	18.54	4.77
Fipronil sulfone	18.60	7.44
Hexadecanedioic acid	19.15	5.05
Dibutyl hexanodioate	19.29	4.22
Citroflex A-4	19.34	6.49
Benazol P	19.38	4.30
Monobutyl phthalate	19.53	2.72
Triclosan	19.65	5.17
Tributyl phosphate	19.86	4.26
Galaxolidone	19.96	5.50
Ethyl Oleate	20.25	8.69
4-tert-Butylcyclohexyl acetate	20.47	3.96

Dibutyl sebacate	20.89	5.97
Dipentyl phthalate	21.00	5.89
Palmitoyl ethanolamide	21.32	5.82
Hexadecanamide	21.48	6.84
Palmitoleic acid	21.51	6.64
Oleamide	21.56	7.07
Avermectin B1a (Abamectin)	21.57	6.51
Benzyl octyl adipate	21.58	6.50
Triethyleneglycol bis(2-ethylhexanoate)	21.66	5.57
Bis(2-ethylhexyl)adipate	22.04	7.85
Salinomycin	22.06	6.10
1-Stearoylglycerol	22.11	7.23
Ivermectin B1a	22.19	6.61
Nigericin	22.26	4.82
Octyl decyl phthalate	22.39	9.34
Stearamide	22.45	6.70
2,2-Methylenebis(4-ethyl-6-tert-butylphenol)	22.90	6.24
Dodecyl sulfate	22.92	5.39
Erucamide	22.93	9.74
Irgafos 168	25.10	12.56

Table S4. Identified compounds and spectrometric and chromatographic criteria.

Compound (CAS number)	Structure	Δ Mass (ppm)[a]	Isotopic pattern (SFit %)[b]	MS^2 match[c]	Rt_{exp} (min)[d]	Rt_{theo} (min)[e]	MS^3 Match[f]	Description [Reference]	Detected areas[g]	Maximum estimated concentration (pg m^{-3})[h]
1,8-Diazabicyclo [5.4.0]undec-7-ene (6674-22-2)	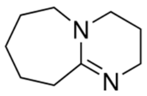	0.01	95	77.1	3.32	5.75	f1, f2	Catalyst/Endogenous metabolite	U	15.68
1-aminocyclohexanecarboxylic acid (2756-85-6)	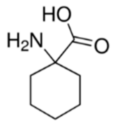	0.29	88	90.3	3.94	3.97	MS^3 (CD)	Aminoacid	R	38.38
1-(carboxymethyl)cyclohexanecarboxylic acid (67950-95-2)	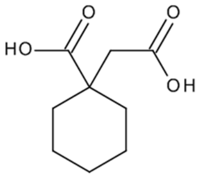	0.05	70	80.1	4.71	5.87	MS^3 (CD)	Pharmaceutical Secondary Standard	U, R, I	94.36
12-Aminododecanoic acid (693-57-2)	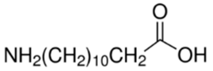	0.12	86	82.8	13.66	13.16	MS^3 (CD)	Bacterial metabolite/corrosion inhibitor	R, I	157.13

2-Amino-1,3,4-octadecanetriol (13552-11-9)	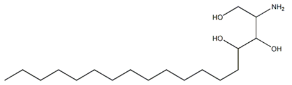	−0.03	92	70.3	16.59	16.48	MS^3 (CD)	Antiasthmatics/ Endogenous metabolite	R	9.23
2-methyl-4-isothiazolin-3-one (2682-20-4)	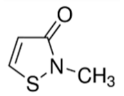	0.14	92	98.4	1.44	1.67	n.a.	Biocide/ Extractable&Leachables	U, R ,I	100.60
3-aminophenol (591-27-5)	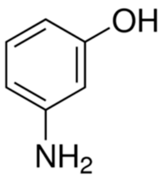	0.12	78	97	0.79	1.87	n.a.	Pesticide/Industrial Chemicals	U, R, I	47.82
3-hydroxy-2-methylpyridine (1121-25-1)	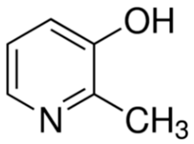	0.1	81	98.9	0.85	0.85*	n.a.	Intermediate in vitamin B6 metabolism	U, R, I	40.40 [Ref st]
3-Methyladenine (5142-23-4)	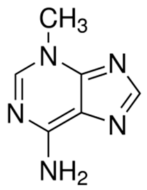	−0.04	91	95.6	0.80	0.80*	MS^3 (CD)	Human metabolite/Endogenous metabolite	U, I	8.86 [Ref st]
3-(2-methylpropyl)-octahydropyrrolo[1,2-a]pyrazine-1,4-dione (2873-36-1)	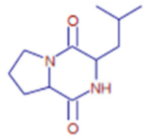	0.06	81	96.6	6.12	6.91	MS^3 (CD)	Amino acid-Flavouring ingredient	U, I	50.46
3,5-dimethoxyaniline (10272-07-8)	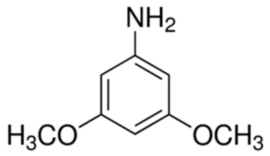	−0.01	70	71.7	0.80	2.55	n.a.	Medication	U	9.75

4-Pentylaniline (33228-44-3)	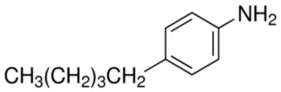	0.21	96	81.9	5.31	10.20	n.a.	Antimicrobial, colorant/ Extractables&Leachables	U, R, I	15.50
4,4'-Methylenebis(2-methylaniline) (838-88-0)	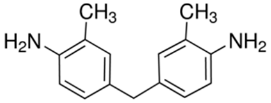	−0.07	70	73.1	2.96	5.70	n.a.	Primary aromatic amine (cosmetic, food, personal care)	I	16.56
5-Chloro-2-methyl-4-isothiazolin-3-one (26172-55-4)	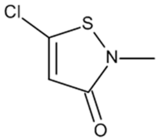	0.11	90	78.6	10.57	10.05	f1, f2	Biocide/Excipients-additives-colorants	R	18.00
7-hydroxy-1,4a-dimethyl-9-oxo-7-(propan-2-yl)-1,2,3,4,4a,4b,5,6,7,9,10,10a-dodecahydrophenanthrene-1-carboxylic acid	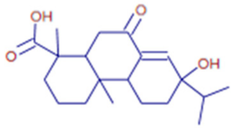	−0.24	86	75	16.72	15.48	f2	Endogenous metabolite, natural product/medicine	U, R, I	173.34
Anhydroecgonine (127379-23-1)	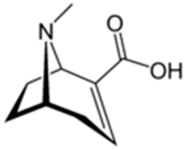	0.08	78	71.9	0.88	1.70	MS^3 (CD)	Chemical intermediate from cocaine/Drugs of abuse/Illegal drugs	U, I	16.20

Azoxystrobin (131860-33-8)	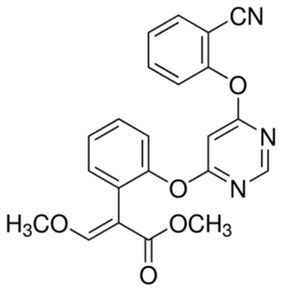	0.18	88	94.3	15.10	15.10*	MS^3 (CD)	Pesticide/fungicide	U	36.25 [Ref st]
Benzenesulfonamide (98-10-2)	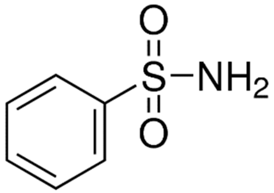	0.15	81	82.5	12.18	5.20	MS^3 (CD)	Inhibitor of human carbonic anhydrase B/Textile chemicals/auxilliary/dyes	R	17.41
Betaine (107-43-7)	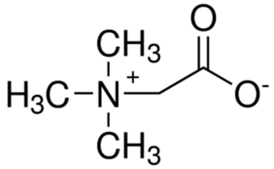	−0.03	79	94.3	7.48	6.86	n.a.	Inhibitor/Endogenous metabolite/lipotropic drug	R	12.29
Bis(2-ethylhexyl)adipate (103-23-1)	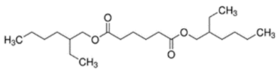	0.03	83	88.1	22.55	22.66	MS^3 (CD)	Plasticizer/Industrial chemicals/Personal care products	U, R, I	374.79
Bis(2-ethylhexyl) amine (106-20-7)	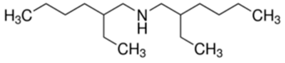	0.1	86	94.5	15.87	23.30	MS^3 (CD)	Surfactant/Industrial chemicals	U	19.48

Butyl 4-aminobenzoate (94-25-7)	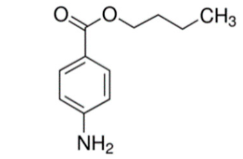	0.09	99	81.9	16.32	15.10	MS^3 (CD)	Local anaesthetic/Therapeutics/prescription drugs	R	14.45
Citroflex 2 (77-93-0)	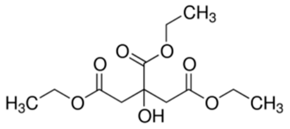	0.09	73	97.8	10.87	8.50	MS^3 (CD)	Plasticizer/Industrial chemicals	U, R, I	42.38
Cocaine (50-36-2)	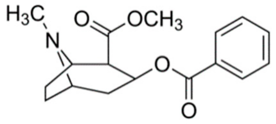	0.18	70	95.5	6.25	6.26	MS^3 (CD)	Drugs	U	28.13 [Ref st]
Cotinine (15569-85-4)	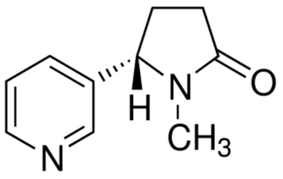	0.2	70	71.3	1.03	1.03	f2	Major metabolite of nicotine/Endogenous metabolite	U, I	67.48 [Ref st]
Cyclo(phenylalanyl-prolyl) (3705-26-8)	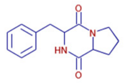	0.14	91	93.7	7.27	7.93	MS^3 (MzCloud)	Metabolite of organic compounds	U	12.20
Decanamide (2319-21-1)	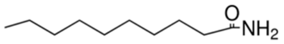	0.15	84	91.3	17.07	16.68	f1, f2	Derives from decanoic acid	U, R, I	111.18

Dextrorphan (125-73-5)	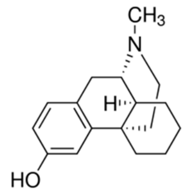	0.33	93	90.1	14.57	14.28	f2	Pyschoactive drugs	R	21.67
Diethyl phosphate (598-02-7)	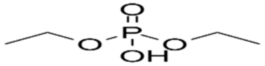	0.01	96	97.8	8.85	5.05	MS^3 (CD)	Metabolite of chlorpyrifos	U, R, I	24.36
Dibenzylamine (103-49-1)	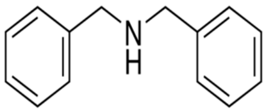	0.01	77	98.4	5.88	8.72	MS^3 (CD)	Indirect additives food contact	U	86.46
Dicyclohexyl phthalate (84-61-7)	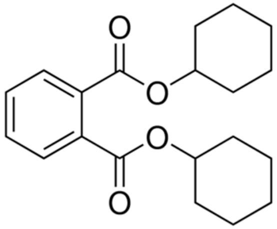	0.1	78	96.4	21.01	21.04	MS^3 (CD)	Adhesive/Industrial chemicals	R, I	121.70
Erucamide (112-84-5)	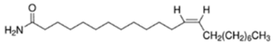	0.24	71	96.6	22.88	23.18	MS^3 (CD)	Slip promoter/Industrial chemicals	U, R, I	64.61

Ethoprophos (13194-48-4)	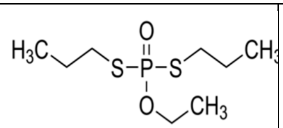	0.21	83	75.8	16.70	16.69	MS^3 (CD)	Nematicide/insecticide	U, I	37.67 [Ref st]
Imidacloprid (138261-41-3)	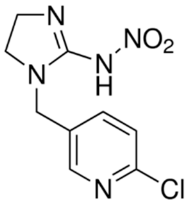	−0.09	71	97.8	5.32	5.32	MS^3 (MzCloud)	Insecticide	U	20.31 [Ref st]
L-Glutamic acid (617-65-5)	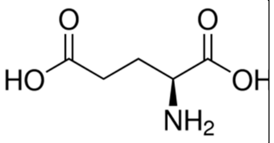	0.22	71	89.7	0.79	0.52	MS^3 (CD)	Peptide/Endogenous metabolite	R	11.25
Metalaxyl (57837-19-1)	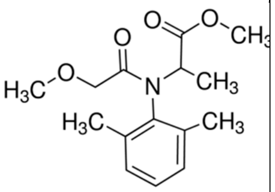	0.2	89	97.1	13.37	13.37	MS^3 (CD)	Fungicide	U	14.24 [Ref st]
N-Benzylethanolamine (104-63-2)	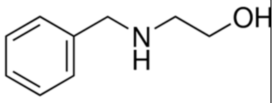	0.03	84	85.2	2.00	3.01	MS^3 (CD)	Corrosion inhibitor	R	112.91
N,N-dimethyldecylamine-N-oxide (2605-79-0)	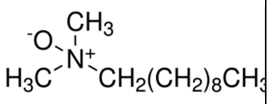	−0.17	84	94.5	13.70	14.17	f1, f2	Environmental contaminant/xenobiotic/Industrial chemicals	U, R, I	119.45

N,N'-Diphenylguanidine (20277-92-3)	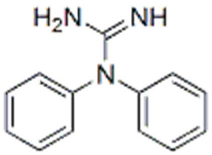	−0.18	81	96.7	4.62	7.03	f1, f2	Complexing agent	U, R, I	251.29
N-Methylcaprolactam (2556-73-2)	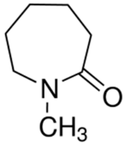	−0.06	71	70.9	6.55	6.22	n.a.	Catalyst	R	32.16
Norharman (244-63-3)	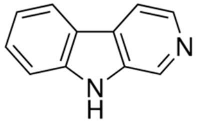	0.17	86	96.8	4.21	5.29	f1, f2	Repellent/Endogenous metabolite	U, R, I	15.82
Nicotine (22083-74-5)	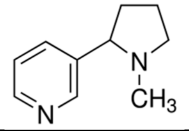	0.23	91	96.4	0.84	0.84	f2	Insecticide/drugs	U, I	55.67 [Ref st]
o-toluidine (1885-29-6)	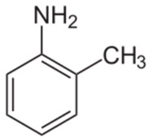	−0.1	88	86.3	0.85	2.59	n.a.	Intermediate in the synthesis of the large-volume herbicides	R, U	68.51
Oxepanone (502-44-3)	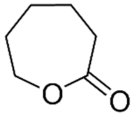	0.01	94	94.4	7.48	6.86	n.a.	Lactone	R, I	13.19

Pyrimethanil (53112-28-0)	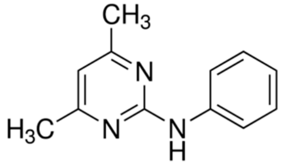	−0.03	89	98.5	10.36	10.34	f1, f2	Fungicide	U	21.84 Ref st
Scoparone (120-08-1)	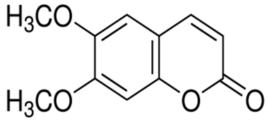	0.2	90.1	98	7.59	7.90	MS³ (MzCloud)	Natural organic compound	I	29.99
Triethyl phosphate (78-40-0)	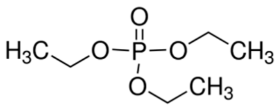	0.04	87	98.7	8.85	7.89	MS³ (CD)	Flame retardant	U, R, I	246.33
Triisopropanolamine (122-20-3)	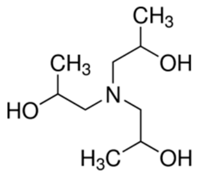	−0.38	70	92.2	0.82	0.83	MS³ (CD)	Amine/Employed in textiles and cosmetics	R	17.23 Ref st
Valpromide (2430-27-5)	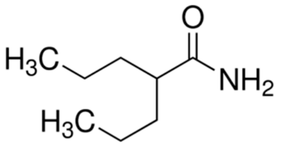	0.26	76	93	10.57	10.05	n.a.	Metabolite from valproic acid/Prescription drug	R	19.39

[a]Δmass (ppm): mass error

[b]Isotopic pattern (SFit %): match between the experimental isotopic pattern and the theoretical one

[c]MS^2 match: match between the experimental and the MS^2 spectra in the mzCloud library

[d]Rt_{exp}: experimental retention time;

[e]Rt_{pred}: predicted retention time based on log P

[f]MS^3 match: match between the experimental and the MS^3 spectra in the mzCloud library: n.a. (MS^3 not available in CD); f1 (mzCloud does not contain a MS^3 spectra of this compound); f2 (the experimental MS^3 spectra do not match with that stored in mzCloud or with the in-silico fragmentation)

[g]Detected areas: I= Industrial; U=urban; R=rural

[h]Maximum estimated concentration: Maximum concentration taking into account the maximum area detected and the average response factor of the seven substances employed in the QC_{RM} (Ref st: semiquantification

performed with its own reference standard)

Table S5. Compounds more probably present in industrial areas (Industrial-Rural).

Compound	Formula	Log2 Fold Change	*p*-value	Identified level
3-Methyladenine	$C_6H_7N_5$	4.67	5.45×10^{-10}	Level 1
Anhydroecgonine	$C_9H_{13}NO_2$	4.06	4.11×10^{-7}	Level 1
Nicotine	$C_{10}H_{14}N_2$	3.58	2.49×10^{-9}	Level 1
3-Hydroxy-2-methylpyridine	C_6H_7NO	3.56	2.29×10^{-9}	Level 1
3-(3-pyridinyl)propanoic acid	$C_8H_9NO_2$	4.10	1.50×10^{-9}	Level 2 (ms3)
Nikethamide	$C_{10}H_{14}N_2O$	3.21	5.45×10^{-10}	Level 2 (ms2)
Phenacetin	$C_{10}H_{13}NO_2$	3.27	7.43×10^{-9}	Level 2 (ms3)
2-Hydroxyphenylalanine	$C_9H_{11}NO_3$	3.46	5.63×10^{-10}	Level 2 (ms3)
3-(2,6-Dioxocyclohexyl)propanenitrile	$C_9H_{11}NO_2$	4.53	$5.76 \times 10_{-10}$	Level 2 (ms3)
5-(Cyanomethyl)-1H-imidazole-4-carbonitrile	$C_6H_4N_4$	5.59	1.26×10^{-7}	Level 2 (ms3)
2,2,2-Trifluoro-1-(1-naphthyl)ethanol	$C_{12}H_9F_3O$	5.12	2.10` $\times 10^{-6}$	Level 2 (ms2)
4-Hydroxybenzaldehyde	$C_7H_6O_2$	5.25	$5.45 \times 10^{--10}$	Level 2 (ms2)
ZV4	$C_5H_{11}NO$	5.60	3.07×10^{-6}	Level 2 (ms2)
Ethyl 4-hydroxy-3-methoxyphenylacetate	$C_{11}H_{14}O_4$	5.83	9.92×10^{-8}	Level 2 (ms2)
Hydroferulic acid	$C_{10}H_{12}O_4$	6.85	7.01×10^{-10}	Level 2 (ms3)
3,5-Dimethoxy-4-hydroxybenzoate	$C_9H_{10}O_5$	5.40	5.51×10^{-10}	Level 2 (ms3)
Ethyl gallate	$C_9H_{10}O_5$	4.41	5.2×10^{-10}	Level 2 (ms3)
Pyroquilon	$C_{11}H_{11}NO$	3.01	5.77×10^{-10}	Level 3
6-Methoxyquinoline	$C_{10}H_9NO$	3.18	5.45×10^{-10}	Level 3
3-hydroxy-3-methylpentanedioic acid	$C_6H_{10}O_5$	3.86	2.44×10^{-8}	Level 3
4,6-Bis(1-aziridinyl)-N-(2,2-dimethyl-1,3-dioxan-5-yl)-1,3,5-triazin-2-amine	$C_{13}H_{20}N_6O_2$	3.72	1.81×10^{-8}	Level 3
Methyl 2-oxo-4-(trifluoromethyl)cyclohexanecarboxylate	$C_9H_{11}F_3O_3$	3.92	1.06×10^{-9}	Level 3
Diaminotoluene	$C_7H_{10}N_2$	3.23	7.32×10^{-09}	Level 3
Ethyl 1-hydroxy-2,3,4,9-tetrahydro-1H-carbazole-6-carboxylate	$C_{15}H_{17}NO_3$	3.90	5.45×10^{-10}	Level 3
1-propylimidazole	$C_6H_{10}N_2$	4.01	1.70×10^{-8}	Level 3
3,3,3-Trifluoro-2-(2-furylmethyl)propanoic acid	$C_8H_7F_3O_3$	3.74	5.54×10^{-10}	Level 3
N'-[5-chloro-2-(4-chlorophenoxy)-4-(1H-pyrrol-1-yl)phenyl]-N,N-dimethyliminoformamide	$C_{19}H_{17}Cl_2N_3O$	5.08	5.45×10^{-10}	Level 3
Methyl [5-methoxy-2-nitro-4-(trifluoromethyl)phenyl]acetate	$C_{11}H_{10}F_3NO_5$	6.95	1.07×10^{-9}	Level 3
6-Methylnicotinonitrile	$C_7H_6N_2$	3.07	5.65×10^{-10}	Level 3
5-acetyl-2,6-dimethyl-1,2,3,4-tetrahydropyridin-4-one	$C_9H_{13}NO_2$	4.94	5.45×10^{-10}	Level 3
1-(Diazidomethyl)-2-nitrobenzene	$C_7H_5N_7O_2$	3.65	5.45×10^{-10}	Level 3
5,7-dihydroxy-2-(3-hydroxy-4-methoxyphenyl)-3,6-dimethoxy-4H-chromen-4-one	$C_{18}H_{16}O_8$	3.81	5.45×10^{-10}	Level 3

Compound	Formula	Log2 Fold Change	*p*-value	Identified level
4-Nitro-2-(1H-tetrazol-5-yl)aniline	$C_7H_6N_6O_2$	5.02	5.62×10^{-10}	Level 3
Xanthone	$C_{13}H_8O_2$	3.18	5.45×10^{-10}	Level 3
Fenuron	$C_9H_{12}N_2O$	3.26	5.45×10^{-10}	Level 3
1,2,3,4-Tetramethyl-1,3-cyclopentadiene	C_9H_{14}	3.16	5.10×10^{-6}	Level 3
2,7-Difluoro-4,5-dimethoxy-2'H,5'H-spiro[fluorene-9,4'-imidazolidine]-2',5'-dione	$C_{17}H_{12}F_2N_2O_4$	7.53	1.54×10^{-9}	Level 3
3,5-Octadiyne	C_8H_{10}	3.14	1.01×10^{-9}	Level 3
N'-[(2,4-dihydroxyphenyl)methylene]-4-methylbenzene sulfonohydrazide	$C_{14}H_{14}N_2O_4S$	3.29	1.24×10^{-08}	Level 3
1-Naphthylisocyanate	$C_{11}H_7NO$	4.43	5.65×10^{-10}	Level 3
(1S,8S,9S,10S,13R)-6,9,10-trimethyl-2-oxo-4,14-dioxatetracyclo[7.5.0.0Âa,ÂaÂl.0Âl,â ·]tetradeca-3(7),5-dien-8-yl acetate	$C_{17}H_{20}O_5$	3.46	5.45×10^{-10}	Level 3
8-Hydroxyquinoline	C_9H_7NO	5.71	5.75×10^{-10}	Level 3
Umbelliferone	$C_9H_6O_3$	3.12	5.45×10^{-10}	Level 3
S-(Allylcarbamothioyl)-L-cysteine	$C_7H_{12}N_2O_2S_2$	3.20	5.45×10^{-10}	Level 3
Dihydrothymine	$C_5H_8N_2O_2$	4.21	3.97×10^{-7}	Level 3
3-hydroxybenzylhydrazine	$C_7H_{10}N_2O$	4.11	5.79×10^{-10}	Level 3
Serotonin	$C_{10}H_{12}N_2O$	4.44	5.55×10^{-10}	Level 3
2-(5-Amino-1H-pyrazol-1-yl)ethanol	$C_5H_9N_3O$	4.45	1.17×10^{-9}	Level 3
119183	$C_7H_7N_3$	3.16	6.22×10^{-10}	Level 3
(4-Methyl-1H-imidazol-5-yl)methanol	$C_5H_8N_2O$	3.38	2.07×10^{-9}	Level 3
2-Methyl-2H-indazol-4-amine	$C_8H_9N_3$	4.75	5.45×10^{-10}	Level 3
6-Methyl-2-pyridinemethanol	C_7H_9NO	3.76	1.34×10^{-6}	Level 3
Aminobenzodiazapine	$C_9H_{11}N_3O$	4.38	5.45×10^{-10}	Level 3
11-Aminoundecanoic acid	$C_{11}H_{23}NO_2$	4.94	5.45×10^{-10}	Level 3
7-Amino-3,4-dihydro-2(1H)-quinoxalinone	$C_8H_9N_3O$	4.60	8.70×10^{-10}	Level 3
4-[3-(5H-Tetrazol-5-ylidene)triazanyl]benzoic acid	$C_8H_7N_7O_2$	4.27	5.45×10^{-10}	Level 3
2-NP-AHD	$C_{10}H_8N_4O_4$	4.62	5.48×10^{-10}	Level 3
4,6-Diamino-1-(4-nitrophenyl)-1,3,5-triazin-2(1H)-one	$C_9H_8N_6O_3$	4.95	2.82×10^{-06}	Level 3
Methyl piperonyl ketone	$C_{10}H_{10}O_3$	4.42	5.77×10^{-10}	Level 3
4,7-dioxosebacic acid	$C_{10}H_{14}O_6$	4.11	9.78×10^{-9}	Level 3
6-Methoxy-2-methyl-3-(3-oxobutyl)-4(1H)-quinolinone	$C_{15}H_{17}NO_3$	3.15	5.45×10^{-10}	Level 3
4-{(E)-[(5-Amino-1H-tetrazol-1-yl)imino]methyl}benzaldehyde	$C_9H_8N_6O$	3.16	8.03×10^{-10}	Level 3
NP-018817	$C_{15}H_{24}O_4$	3.20	8.64×10^{-4}	Level 3
Ethyl 5-formyl-2,4-dimethyl-1H-pyrrole-3-carboxylate	$C_{10}H_{13}NO_3$	3.79	2.48×10^{-9}	Level 3
Glutaric acid	$C_5H_8O_4$	5.61	7.21×10^{-10}	Level 3

**Compound**	**Formula**	**Log2 Fold Change**	***p*-value**	**Identified level**
p-Xylylenediamine	$C_8H_{12}N_2$	3.18	5.45×10^{-10}	Level 3
6-Vinylnicotinonitrile	$C_8H_6N_2$	4.30	5.45×10^{-10}	Level 3
1-deoxy-1-(2,4-difluorophenyl)-?-D-ribofuranose	$C_{11}H_{12}F_2O_4$	5.80	5.61×10^{-10}	Level 3
(-)-Ecgonine methyl ester	$C_{10}H_{17}NO_3$	3.90	1.59×10^{-9}	Level 3
(2E)-3-(3,4-Dimethoxyphenyl)acrylic acid	$C_{11}H_{12}O_4$	4.17	5.45×10^{-10}	Level 3
NP-019992	$C_{10}H_{12}O_4$	3.17	2.34×10^{-5}	Level 3
1-propyl-1H-benzo[d]imidazole hydrobromide	$C_{10}H_{12}N_2$	3.12	1.21×10^{-7}	Level 3
Ethyl 4,4,4-trifluoro-3-hydroxy-3-methoxybutanoate	$C_7H_{11}F_3O_4$	3.47	5.45×10^{-10}	Level 3
Methyl 4-hydroxy-3-methoxy-5-(trifluoromethyl)benzoate	$C_{10}H_9F_3O_4$	4.03	6.04×10^{-10}	Level 3
4-Tolylurea	$C_8H_{10}N_2O$	3.87	1.13×10^{-9}	Level 3
NP-020713	$C_{20}H_{26}O_4$	4.20	5.45×10^{-10}	Level 3
7-Aminoindole	$C_8H_8N_2$	3.69	2.08×10^{-9}	Level 3
2,3,5-Trimethoxy-1,4-benzenediol	$C_9H_{12}O_5$	3.22	5.45×10^{-10}	Level 3
Combretastatin	$C_{18}H_{22}O_6$	3.55	5.45×10^{-10}	Level 3
7beta,12alpha-Dihydroxykaurenolide	$C_{20}H_{28}O_4$	3.12	5.06×10^{-8}	Level 3
Syringaldehyde	$C_9H_{10}O_4$	4.40	5.46×10^{-10}	Level 3
Penicillic-Acid	$C_8H_{10}O_4$	3.12	5.45×10^{-10}	Level 3
4-Methoxysalicylic acid	$C_8H_8O_4$	3.72	5.45×10^{-10}	Level 3
4-Methylumbelliferone hydrate	$C_{10}H_8O_3$	3.17	5.45×10^{-10}	Level 3
1,3-Octadiyne	C_8H_{10}	3.01	1.25×10^{-9}	Level 3
3-[(4-Phenyl-2-butanyl)amino]propanenitrile	$C_{13}H_{18}N_2$	3.29	2.06×10^{-9}	Level 3
Metrafenone	$C_{19}H_{21}BrO_5$	7.20	5.45×10^{-10}	Level 3
(1R,2R)-1-(4-Hydroxy-3-methoxyphenyl)-1,2,3-propanetri ol	$C_{10}H_{14}O_5$	3.74	5.45×10^{-10}	Level 3
4-(2-Methyl-2-propanyl)-2-(trifluoromethyl)cyclohexano ne	$C_{11}H_17F_3O$	4.78	3.74×10^{-6}	Level 3
Dimethyl (3-oxocyclohexyl)malonate	$C_{11}H_{16}O_5$	4.29	5.45×10^{-10}	Level 3
Radicinin	$C_{12}H_{12}O_5$	3.74	5.45×10^{-10}	Level 3
2-Methoxy-5-(trifluoromethoxy)benzyl alcohol	$C_9H_9F_3O_3$	3.62	5.45×10^{-10}	Level 3
2-deoxyglucose	$C_6H_{12}O_5$	4.43	1.28×10^{-9}	Level 3
4-Phenyl-2-(trifluoromethyl)-1,2,4-butanetriol	$C_{11}H_{13}F_3O_3$	4.20	5.45×10^{-10}	Level 3
1,1,2,2,3,3,4,4,5,5-Cyclopentanedecol	$C_5H_{10}O_{10}$	4.73	5.45×10^{-10}	Level 3
7-hydroxy-6-methoxy-2H-chromen-2-one	$C_{10}H_8O_4$	4.49	5.45×10^{-10}	Level 3
N4-(3-chloro-4-fluorophenyl)-6-methylpyrimidine-2,4-di amine hydrochloride	$C_{11}H_{10}ClFN_4$	4.79	5.45×10^{-10}	Level 3
5,6,7-Trimethoxy-2H-chromen-2-one	$C_{12}H_{12}O_5$	4.02	2.36×10^{-7}	Level 3
MW5143500	$C_9H_{10}O_4$	3.69	5.45×10^{-10}	Level 3
NP-002855	$C_{12}H_{22}O_4$	3.19	5.45×10^{-10}	Level 3
dihydralazine	$C_8H_{10}N_6$	3.41	8.06×10^{-5}	Level 3
3,4-Dimethoxy-5-(3,3,3-trifluoropropyl)benzoic acid	$C_{12}H_{13}F_3O_4$	4.74	5.46×10^{-10}	Level 3

Compound	Formula	Log2 Fold Change	*p*-value	Identified level
3964	$C_7H_8N_2O$	3.64	5.45×10^{-10}	Level 3
(1S,4R,7S,8S,11S)-9-tert-butyl-7,9-dihydroxy-3,5,12-trioxatetracyclo[6.6.0.0Âa,ÂaÂa.0â,â]tetradecane-2,6,13-trione	$C_{15}H_{18}O_8$	4.62	5.46×10^{-10}	Level 3
6,7-Dihydro-8(5H)-quinolinone	C_9H_9NO	3.17	1.85×10^{-9}	Level 3
2,2,2-Trifluoro-1-(3,4,5-trimethoxyphenyl)ethanol	$C_{11}H_{13}F_3O_4$	3.53	5.45×10^{-10}	Level 3
Aldicarb oxime	$C_5H_{11}NOS$	3.32	7.57×10^{-10}	Level 3
Methyl 2-{[(4-oxo-3,4-dihydrophthalazin-1-yl)methyl]thio}acetate	$C_{12}H_{12}N_2O_3S$	4.02	5.45×10^{-10}	Level 3
NSC 131681	C_8H_9NO	4.06	7.30×10^{-10}	Level 3
5-Ethylcyclohexane-1,3-dione	$C_8H_{12}O_2$	4.79	2.29×10^{-9}	Level 3
Cyclodecyl trifluoroacetate	$C_{12}H_{19}F_3O_2$	3.51	5.45×10^{-10}	Level 3
4,7-dihydroxy-4-(hydroxymethyl)-3,4a,8,8-tetramethyl-1,4,4a,5,6,7,8,8a-octahydronaphthalen-1-one	$C_{15}H_{24}O_4$	4.01	3.30×10^{-8}	Level 3
hymecromone	$C_{10}H_8O_3$	3.09	5.45×10^{-10}	Level 3
2-Diazonio-1-({2-[(2,4-dinitrophenyl)amino]ethyl}amino)ethenolate	$C_{10}H_{10}N_6O_5$	4.73	5.45×10^{-10}	Level 3
4-Nitro-N-(1H-tetrazol-5-yl)benzamide	$C_8H_6N_6O_3$	3.87	5.45×10^{-10}	Level 3
4-(3,4-DIMETHOXYPHENYL)BUTYRIC ACID	$C_{12}H_{16}O_4$	4.06	5.45×10^{-10}	Level 3
NP-019983	$C_{20}H_{16}O_6$	4.20	5.49×10^{-10}	Level 3
[(3S)-3-(5-Benzyl-1,3,4-oxadiazol-2-yl)-1-pyrrolidinyl](4-pyridinyl)methanone	$C_{19}H_{18}N_4O_2$	3.48	5.45×10^{-10}	Level 3
4-decyl-3-hydroxy-5-oxooxolane-2,3-dicarboxylic acid	$C_{16}H_{26}O_7$	6.45	7.47×10^{-7}	Level 3
ethyl 1-(3-nitro-2-thienyl)piperidine-4-carboxylate	$C_{12}H_{16}N_2O_4S$	5.49	5.46×10^{-10}	Level 3
Prohexadione	$C_{10}H_{12}O_5$	4.09	5.45×10^{-10}	Level 3
N-(4-fluorophenyl)-2-methyl-5-(morpholinosulfonyl)-3-furamide	$C_{16}H_{17}FN_2O_5S$	7.44	6.30×10^{-10}	Level 3
Cytidine	$C_9H_{13}N_3O_5$	3.44	5.45×10^{-10}	Level 3
DL-carvone	$C_{10}H_{14}O$	3.02	1.23×10^{-8}	Level 3
Ethyl 3-hydroxy-4,4,4-trifluorobutyrate	$C_6H_9F_3O_3$	5.23	5.75×10^{-7}	Level 3
4-(tert-butyl)phenyl 3,5-dimethylisoxazole-4-carboxylate	$C_{16}H_{19}NO_3$	3.66	5.45×10^{-10}	Level 3
Ethyl orsellinate	$C_{10}H_{12}O_4$	4.22	5.45×10^{-10}	Level 3
3-Formylbenzoic acid	$C_8H_6O_3$	3.50	5.45×10^{-10}	Level 3
dl-Perillaldehyde	$C_{10}H_{14}O$	3.06	9.24×10^{-6}	Level 3
NP-003553	$C_{20}H_{34}O_4$	4.26	6.00×10^{-10}	Level 3
LW8000000	$C_8H_8O_5$	4.51	2.10×10^{-9}	Level 3
8-[4-(3,4,5-Trifluorophenyl)cyclohexyl]-1,4-dioxaspiro[4.5]decane	$C_{20}H_{25}F_3O_2$	3.06	2.64×10^{-6}	Level 3

Compound	Formula	Log2 Fold Change	*p*-value	Identified level
6-anilino-2,4-dioxo-1,2,3,4-tetrahydropyrimidine-5-carbo nitrile	$C_{11}H_8N_4O_2$	5.11	4.08×10^{-5}	Level 3
(-)	$C_7H_{15}N_7O_2$	8.28	5.45×10^{-10}	Level 4
(-)	$C_{14}H_{26}F_3N_6O_8PS$	7.60	5.15×10^{-9}	Level 4
(-)	$C_{12}H_{29}N_4O_4P$	3.71	7.14×10^{-8}	Level 4
(-)	$C_6H_7F_3O_4P_2$	4.71	6.04×10^{-10}	Level 4
(-)	$C_{11}H_{15}F_5N_4OS$	5.68	7.98×10^{-10}	Level 4
(-)	$C_4H_4N_7P$	8.61	4.62×10^{-8}	Level 4
(-)	$C_9H_{13}F_3O_5$	5.57	5.96×10^{-10}	Level 4
(-)	$C_3H_4N_6O_3$	7.11	1.46×10^{-9}	Level 4
(-)	$C_4H_{12}F_2N_5OPS$	5.77	5.48×10^{-10}	Level 4
(-)	$C_{12}H_9N_7O_4P_2$	5.99	3.08×10^{-9}	Level 4
(-)	$C_{15}H_{16}FN_2P_3S$	6.12	5.45×10^{-10}	Level 4
(-)	$C_8H_{10}N_6O_6$	5.55	5.45×10^{-10}	Level 4
(-)	$C_{14}H_{24}N_6O_3$	4.22	3.91×10^{-4}	Level 4
(-)	$C_4H_7FN_2O_5$	7.11	4.23×10^{-9}	Level 4
(-)	$C_6H_{13}F_2N_2O_5P$	6.07	2.51×10^{-6}	Level 4
(-)	$C_{12}H_{17}FN_4O_4S_2$	6.65	1.23×10^{-9}	Level 4
(-)	$C_5H_{12}O_{10}$	4.73	9.29×10^{-7}	Level 4
(-)	$C_{10}H_{22}F_2N_4OS_3$	5.64	6.64×10^{-10}	Level 4
(-)	$C_2H_7N_{10}O_3P$	5.77	1.06×10^{-9}	Level 4
(-)	$C_{14}H_{22}N_2OP_2S$	4.50	5.45×10^{-10}	Level 4
(-)	$C_5H_{16}FN_4P_3$	5.01	5.45×10^{-10}	Level 4
(-)	$C_{21}H_{30}ClO_{10}P$	6.80	5.48×10^{-10}	Level 4
(-)	$C_{11}H_{16}F_3NO$	3.00	5.45×10^{-10}	Level 4
(-)	$C_9H_{13}F_6PS$	3.65	5.63×10^{-10}	Level 4
(-)	$C_{14}H_{13}FS$	3.53	5.45×10^{-10}	Level 4
(-)	$C_4H_8N_{10}P_2$	6.35	9.58×10^{-10}	Level 4
(-)	$C_3H_5Cl_3N_2O_5$	3.56	9.16×10^{-09}	Level 4
(-)	$C_{20}H_{21}F_3O$	3.05	3.20×10^{-05}	Level 4
(-)	$C_{10}H_{11}F_3O_4$	4.53	5.45×10^{-10}	Level 4
(-)	$C_7H_7N_2O_6P$	4.44	5.45×10^{-10}	Level 4
(-)	$C_{20}H_{20}F_4N_2O_{10}$	9.15	5.45×10^{-10}	Level 4
(-)	$C_9H_9F_3O_4$	4.46	5.46×10^{-10}	Level 4
(-)	$C_{16}H_{25}FO_3$	4.45	5.47×10^{-10}	Level 4
(-)	$C_{10}H_{19}O_2P_3$	4.61	6.09×10^{-10}	Level 4
(-)	$C_{14}H_{27}FN_2O_3P_2$	6.36	3.16×10^{-6}	Level 4
(-)	$C_{20}H_{21}F_3O_3$	4.84	5.45×10^{-10}	Level 4

Compound	Formula	Log2 Fold Change	p-value	Identified level
(-)	$C_8H_{15}FN_4O_9S$	8.03	5.45×10^{-10}	Level 4
(-)	$C_6HBrCl_2N_4O$	6.81	5.45×10^{-10}	Level 4

(-) = No name described in CD

Table S6. Compounds more probably present in industrial areas (Industrial-Rural).

Compound	Formula	Log2 Fold Change	p-value	Identified level
Methcathinone	$C_{10}H_{13}NO$	−3.01	8.85×10^{-04}	Level 2 (ms2)
MHPG	$C_9H_{12}O_4$	−3.25	5.35×10^{-10}	Level 2 (ms3)
4-hydroxy-6-undecylpyran-2-one	$C_{16}H_{26}O_3$	−3.12	5.46×10^{-10}	Level 2 (ms3)
Euscaphic acid	$C_{30}H_{48}O_5$	−3.30	5.23×10^{-10}	Level 2 (ms3)
1-(3-Phenyl-prop-2-ynyl)-piperidine	$C_{14}H_{17}N$	−4.07	7.43×10^{-10}	Level 2 (ms2)
5-Ethyl-5-(2-methylbutyl)-1,3-bis(2-oxiranylmethyl)-2,4-imidazolidindion	$C_{16}H_{26}N_2O_4$	−3.40	5.46×10^{-10}	Level 2 (ms3)
8-Amino-7-oxononanoic acid	$C_9H_{17}NO_3$	−5.69	1.25×10^{-04}	Level 2 (ms3)
1-Aminocyclododecanecarboxylic acid	$C_{13}H_{25}NO_2$	−3.22	5.95×10^{-10}	Level 2 (ms2)
(2R,3R,4R,5S,6R)-2-(Hydroxymethyl)-6-propyl-3,4,5-piperidinetriol	$C_9H_{19}NO_4$	−4.41	1.09×10^{-9}	Level 2 (ms3)
2,2'-(1,7-Dioxa-4,10-diazacyclododecane-4,10-diyl)diethanol	$C_{12}H_{26}N_2O_4$	−3.72	6.00×10^{-10}	Level 2 (ms3)
DL-Carnitine	$C_7H_{15}NO_3$	−4.13	5.45×10^{-10}	Level 3
1,3-di-o-Tolylguanidine	$C_{15}H_{17}N_3$	−7.60	5.45×10^{-10}	Level 3
7-Hydroxycoumarinyl-γ-linolenate	$C_{27}H_{34}O_4$	−3.08	2.83×10^{-09}	Level 3
Fenuron	$C_9H_{12}N_2O$	−3.33	5.45×10^{-10}	Level 3
Metolachlor morpholinone	$C_{14}H_{19}NO_2$	−3.71	5.45×10^{-10}	Level 3
Diheptyl phthalate	$C_{22}H_{34}O_4$	−4.48	2.25×10^{-4}	Level 3
N-Boc-2-amino-2-methyl-1-propanol	$C_9H_{19}NO_3$	−4.20	1.13×10^{-4}	Level 3
4-Hydroxy-1-butanesulfonic acid	$C_4H_{10}O_4S$	−4.96	3.98×10^{-4}	Level 3
Adipic acid dihydrazide	$C_6H_{14}N_4O_2$	−3.13	1.35×10^{-9}	Level 3
[2-(hydroxymethyl)-5,5,8a-trimethyl-1,4,4a,5,6,7,8,8a-octahydronaphthalen-1-yl]methanol	$C_{15}H_{26}O_2$	−3.27	8.35×10^{-10}	Level 3
L-Pyroglutamic acid	$C_5H_7NO_3$	−4.65	5.47×10^{-10}	Level 3
Gabapentin	$C_9H_{17}NO_2$	−4.23	5.42×10^{-9}	Level 3
N-Acetylvaline	$C_7H_{13}NO_3$	−3.51	4.79×10^{-4}	Level 3
DL-Glutamine	$C_5H_{10}N_2O_3$	−4.96	5.45×10^{-10}	Level 3
2-Thiophenetellurol	C_4H_4STe	−5.53	5.55×10^{-07}	Level 3
2-Methyl-5,8,11-trioxa-2-azatetradecan-13-ol	$C_{11}H_{25}NO_4$	−3.97	5.61×10^{-10}	Level 3
5-Methyl-4-sulfanyl-2-(2,4,4-trimethyl-2-pentanyl)phenol	$C_{15}H_{24}OS$	−3.62	4.74×10^{-4}	Level 3
Ethephon	$C_2H_6ClO_3P$	-3.23	5.35×10^{-05}	Level 3
(2E)-3-Ethoxy-N-{[3-hydroxy-4-(hydroxymethyl)cyclopentyl]carbamoyl}acrylamide	$C_{12}H_{20}N_2O_5$	-3.70	6.00 × 10−10	Level 3
8-(Dimethylamino)guanosine	$C_{12}H_{18}N_6O_5$	-6.69	9.11×10^{-6}	Level 3
11-Oxoetiocholanolone	$C_{19}H_{28}O_3$	-4.24	5.45×10^{-10}	Level 3
UNII:FD6L8T043R	$C_{16}H_{26}N_2O_4$	-3.62	1.87×10^{-04}	Level 3
4-Nitro-2-(1H-tetrazol-5-yl)aniline	$C_7H_6N_6O_2$	-3.16	8.46×10^{-9}	Level 3

Compound	Formula	Log2 Fold Change	*p*-value	Identified level
2-Methyl-N-[3-(trifluoromethyl)phenyl]propanamide	$C_{11}H_{12}F_3NO$	−6.71	5.45×10^{-10}	Level 3
3603307	$C_9H_{19}NO_3$	−3.32	9.75×10^{-5}	Level 3
2-Methyl-2-propanyl {2-[2-(2-hydroxyethoxy)ethoxy]ethyl}carbamate	$C_{11}H_{23}NO_5$	−4.30	3.25×10^{-9}	Level 3
2-Cyclohexylacetohydrazide	$C_8H_{16}N_2O$	−3.23	5.45×10^{-10}	Level 3
(-)	$C_5HF_3N_2OP_2S_2$	−7.18	8.74×10^{-10}	Level 4
(-)	$C_5H_4F_4N_2S_3$	−5.06	1.80×10^{-09}	Level 4
(-)	$C_{45}H_{91}F_2N_7O_6$	−8.99	5.46×10^{-10}	Level 4
(-)	$C_{18}H_{29}N_6O_2P_3$	−3.07	5.46×10^{-10}	Level 4
(-)	$C_8H_9N_7O_3$	−3.05	2.13×10^{-9}	Level 4
(-)	$C_4HN_3O_7P_2S$	−6.80	6.08×10^{-10}	Level 4
(-)	$C_{17}H_{27}F_5N_7P_3$	−8.36	5.45×10^{-10}	Level 4
(-)	$C_6HF_7N_3O_3P_3S_3$	−6.08	9.08×10^{-10}	Level 4
(-)	C12H24F4N4O2	−3.73	6.61×10^{-10}	Level 4
(-)	$C_9H_4ClF_{10}NO_2P_2S_5$	−7.82	5.64×10^{-10}	Level 4
(-)	$C_2H_5BrF_4N_4S_2$	−5.35	2.95×10^{-9}	Level 4
(-)	$C_{24}H_3F_2O_{17}P_3S_5$	−9.14	5.45×10^{-10}	Level 4
(-)	$C_{10}H_{20}FN_7O_5$	−4.38	1.15×10^{-5}	Level 4
(-)	$C_5H_4ClF_4N_2O_5P_3$	−5.77	3.23×10^{-8}	Level 4
(-)	$C_{56}H_{87}F_{16}N_3O_{18}P_2S_3$	−8.55	5.58×10^{-10}	Level 4
(-)	$C_{12}H_2F_{11}O_4P_3S_3$	−5.58	1.02×10^{-8}	Level 4
(-)	$C_3HF_2N_5O_8P_2S_5$	−6.41	9.16×10^{-8}	Level 4
(-)	$C_4H_4F_3NO_4P_2S_3$	−5.68	5.48×10^{-10}	Level 4
(-)	$C_4H_2F_3N_4O_{14}P_3S_3$	−7.19	1.04×10^{-9}	Level 4
(-)	$C_3H_3N_4O_8PS_3$	−4.65	3.28×10^{-6}	Level 4
(-)	$C_{19}H_{25}F_4NOS$	−3.31	2.01×10^{-9}	Level 4
(-)	$C_{20}H_{45}Cl_2F_3N_8O_5$	−9.32	9.25×10^{-9}	Level 4
(-)	$C_{26}H_{36}O_4S_2$	−3.57	1.01×10^{-9}	Level 4
(-)	$C_{16}H_{38}N_8O_2S$	−6.04	1.42×10^{-9}	Level 4
(-)	$C_4H_{10}FN_3O_4$	−5.26	1.21×10^{-9}	Level 4
(-)	$C_{15}H_{32}FN_2O_{18}P$	−3.21	7.16×10^{-8}	Level 4
(-)	$C_5HF_6O_7P_3S$	−7.56	7.20×10^{-9}	Level 4
(-)	$C_{18}H_{35}FN_2O_6S$	−6.71	8.47×10^{-10}	Level 4
(-)	$C_4H_2F_6O_{18}S_3$	−6.56	5.76×10^{-10}	Level 4
(-)	$C_9H_9F_2O_{17}P_3S_3$	−6.09	6.96×10^{-7}	Level 4
(-)	$C_8H_{10}F_7O_{14}P_3S_4$	−6.83	4.60×10^{-9}	Level 4
(-)	$C_{20}H_{39}FN_2O_4$	−6.51	5.45×10^{-10}	Level 4
(-)	$C_7HF_4O_{16}P_3S$	−8.01	7.06×10^{-10}	Level 4

Compound	Formula	Log2 Fold Change	*p*-value	Identified level
(-)	$C_{10}H_3F_{10}N_4O_{12}PS_5$	−6.96	2.66×10^{-7}	Level 4
(-)	$C_{10}HFN_4O_{16}P_2S_3$	−7.26	7.91×10^{-4}	Level 4
(-)	$C_9H_2F_5O_{16}P_3$	−3.05	2.08×10^{-9}	Level 4
(-)	$C_8HF_2N_4O_9P_3S_2$	−8.39	5.45×10^{-10}	Level 4
(-)	$C_{18}H_{31}FS$	−3.44	6.79×10^{-10}	Level 4
(-)	$C_{28}H_{59}F_2O_5P_3S_4$	−8.13	8.64×10^{-10}	Level 4
(-)	$C_{19}H_{47}ClN_9OP$	−7.83	5.45×10^{-10}	Level 4
(-)	$C_{14}H_{23}F_4N_5O_2$	−3.70	5.55×10^{-10}	Level 4
(-)	$C_5H_6F_2N_2O_8P_2S_3$	−3.33	1.42×10^{-09}	Level 4
(-)	$C_3H_8P_2S$	−6.49	1.16×10^{-09}	Level 4
(-)	$C_5H_{14}FN_2O_7P_3S_5$	-3.16	7.14×10^{-10}	Level 4
(-)	$C_4H_2F_{11}O_4P_3S_2$	−7.73	5.12×10^{-08}	Level 4
(-)	$C_5H_5N_4O_{11}P_3S_3$	−8.39	5.52×10^{-10}	Level 4
(-)	$C_5HF_6N_8O_4P_3S_5$	−8.72	5.45×10^{-10}	Level 4
(-)	$C_8H_{10}BrClN_2OS_3$	−6.65	5.61×10^{-10}	Level 4
(-)	$C_9H_4F_7N_2O_{13}P_3S_5$	−10.96	5.45×10^{-10}	Level 4
(-)	$C_{18}H_{25}F_3N_2O_8$	−3.64	5.45×10^{-10}	Level 4
(-)	$C_6H_2ClF_3O_4$	−8.76	1.13×10^{-9}	Level 4
(-)	$C_2H_2F_5O_2PS$	−6.95	5.23×10^{-7}	Level 4
(-)	$C_3H_3F_3N_5O_8P_3S_3$	−11.07	5.51×10^{-10}	Level 4
(-)	$C_2H_4Cl_2FN_2OPS_2$	−10.82	1.40×10^{-09}	Level 4
(-)	$C_4H_2F_3O_2P_3$	−5.55	5.45×10^{-10}	Level 4
(-)	$C_4H_2F_3O_7P_3S$	−4.55	1.74×10^{-7}	Level 4
(-)	$C_3H_6Cl_2P_2S_2$	−8.68	1.39×10^{-6}	Level 4
(-)	$C_{14}H_{29}FO_3$	−4.28	5.45×10^{-10}	Level 4
(-)	$C_4HF_2O_8P$	−5.49	1.45×10^{-9}	Level 4
(-)	$C_6H_2F_3P$	−7.30	2.63×10^{-9}	Level 4
(-)	$C_6H_3F_{14}O_7P_3S_5$	−8.44	6.24×10^{-10}	Level 4
(-)	$C_{15}H_6F_9O_8P_3S_5$	−8.59	5.52×10^{-10}	Level 4
(-)	$C_{14}H_{27}F_3N_2O_6S_2$	−4.16	1.40×10^{-09}	Level 4
(-)	$C_{13}H_{29}NO_5$	−3.79	5.92×10^{-07}	Level 4
(-)	$C_{44}H_{90}F_3N_8P$	−7.19	5.45×10^{-10}	Level 4
(-)	$C_6HClF_5N_2O_4P_3S$	−6.22	5.46×10^{-10}	Level 4
(−)	$C_9HO_7PS_3$	−5.40	5.45×10^{-10}	Level 4
(−)	$C_{10}H_{17}F_3N_2O_2S_3$	−3.32	5.45×10^{-10}	Level 4
(−)	$C_5H_2Cl_2F_3N_3S_2$	−3.92	9.44×10^{-06}	Level 4
(−)	$C_8HClF_{12}N_3O_{11}P_3S_4$	−9.27	5.45×10^{-10}	Level 4
(−)	$C_8HCl_2F_5N_6OS_4$	−8.29	5.55×10^{-10}	Level 4

Compound	Formula	Log2 Fold Change	*p*–value	Identified level
(−)	$C_{14}H_{21}F_3O_5$	−5.16	1.29×10^{-6}	Level 4
(−)	$C_{49}H_{99}N_4P_3$	−6.31	5.74×10^{-10}	Level 4
(−)	$C_4H_5N_2PS_2$	−3.31	3.12×10^{-8}	Level 4
(−)	$C_{11}H_{29}F_5N_8O_{17}P_2S$	−9.79	5.45×10^{-10}	Level 4
(−)	$C_{53}H_{97}F_2NO_2S$	−9.29	5.48×10^{-10}	Level 4
(−)	$C_{14}H_2F_2O_{14}P_2S_3$	−4.70	6.08×10^{-9}	Level 4
(−)	$C_3H_2F_2O_7P_2S$	−7.04	4.90×10^{-8}	Level 4
(−)	$C_3H_2BrF_2N_9O_3S_3$	−9.99	2.09×10^{-9}	Level 4
(−)	$C_{10}H_{20}F_2N_6O_2S$	−4.92	1.46×10^{-8}	Level 4
(−)	$C_6H_9F_3N_2S_2$	−5.42	5.45×10^{-10}	Level 4
(−)	(−) (m/z=115.97859)	−3.88	5.45×10^{-10}	Level 5
(−)	(−) (m/z=863.69976)	−8.70	5.49×10^{-10}	Level 5
(−)	(−) (m/z=647.43803)	−8.40	5.45×10^{-10}	Level 5
(−)	(−) (m/z=656.10704)	−8.60	5.45×10^{-10}	Level 5
(−)	(−) (m/z=123.96857)	−3.05	4.82×10^{-9}	Level 5
(−)	(−) (m/z=436.51015)	−8.26	5.45×10^{-10}	Level 5
(−)	(−) (m/z=777.39584)	−8.06	5.53×10^{-10}	Level 5
(−)	(−) (m/z=734.40917)	−8.29	5.47×10^{-10}	Level 5
(−)	(−) (m/z=119.97166)	−7.93	5.45×10^{-10}	Level 5
(−)	(−) (m/z=793.72866)	−9.03	5.45×10^{-10}	Level 5
(−)	(−) (m/z=840.69745)	−8.22	5.53×10^{-10}	Level 5
(−)	(−) (m/z=489.83934)	−8.47	5.45×10^{-10}	Level 5
(−)	(−) (m/z=820.71495)	−8.82	5.45×10^{-10}	Level 5
(−)	(−) (m/z=735.07365)	−9.01	5.45×10^{-10}	Level 5
(−)	(−) (m/z=821.71117)	−7.78	6.01×10^{-10}	Level 5
(−)	(−) (m/z=547.83043)	−7.81	1.97×10^{-5}	Level 5
(−)	(−) (m/z=609.80086)	−7.55	2.91×10^{-6}	Level 5
(−)	(−) (m/z=696.42571)	−8.63	6.02×10^{-5}	Level 5
(−)	(−) (m/z=541.81348)	−8.14	8.20×10^{-6}	Level 5
(−)	(−) (m/z=539.80515)	−8.05	6.63×10^{-10}	Level 5
(−)	(−) (m/z=517.14610)	−8.36	5.45×10^{-10}	Level 5
(−)	(−) (m/z=442.51360)	−8.03	5.45×10^{-10}	Level 5
(−)	(−) (m/z=983.67282)	−8.16	5.45×10^{-10}	Level 5
(−)	(−) (m/z=609.78801)	−8.05	5.45×10^{-10}	Level 5
(−)	(−) (m/z=723.75475)	−8.61	2.51×10^{-6}	Level 5
(−)	(−) (m/z=778.39331)	−8.04	5.74×10^{-10}	Level 5
(−)	(−) (m/z=820.38066)	−8.71	5.46×10^{-10}	Level 5
(−)	(−) (m/z=817.69453)	−8.50	6.09×10^{-10}	Level 5
(−)	(−) (m/z=538.80125)	−10.88	1.33×10^{-9}	Level 5
(−)	(−) (m/z=482.83147)	−10.69	5.50×10^{-10}	Level 5
(−)	(−) (m/z=723.75762)	−6.55	1.21×10^{-09}	Level 5
(−)	(−) (m/z=603.78429)	−9.24	5.45×10^{-10}	Level 5
(−)	(−) (m/z=117.97467)	−3.34	3.23×10^{-8}	Level 5
(−)	(−) (m/z=102.94277)	−3.47	3.74×10^{-5}	Level 5
(−)	(−) (m/z=835.70505)	−10.14	7.70×10^{-9}	Level 5
(−)	(−) (m/z=101.96297)	−5.42	1.59×10^{-4}	Level 5
(−)	(−) (m/z=119.97352)	−3.78	7.21×10^{-9}	Level 5
(−)	(−) (m/z=129.95791)	−7.44	1.60×10^{-6}	Level 5
(−)	(−) (m/z=133.98920)	−3.76	5.45×10^{-10}	Level 5

(−)	(−) (m/z=147.96855)	−3.33	1.67×10^{-6}	Level 5
(−)	(−) (m/z=133.98922)	−6.54	5.54×10^{-10}	Level 5
(−)	(−) (m/z=473.81270)	−6.52	5.45×10^{-10}	Level 5
(−)	(−) (m/z=119.97166)	−7.93	2.07×10^{-8}	Level 5
(−)	(−) (m/z=668.76319)	−10.25	5.60×10^{-10}	Level 5
(−)	(−) (m/z=690.75736)	−8.86	5.45×10^{-10}	Level 5
(−)	(−) (m/z=547.81046)	−8.80	1.10×10^{-9}	Level 5
(−)	(−) (m/z=105.95588)	−3.26	1.15×10^{-7}	Level 5
(−)	(−) (m/z=777.06124)	−9.46	5.46×10^{-10}	Level 5
(−)	(−) (m/z=117.98287)	−3.36	5.45×10^{-10}	Level 5
(−)	(−) (m/z=690.42275)	−9.38	5.46×10^{-10}	Level 5
(−)	(−) (m/z=647.10355)	−9.70	5.45×10^{-10}	Level 5
(−)	(−) (m/z=784.72349)	−9.41	5.45×10^{-10}	Level 5
(−)	(−) (m/z=671.77350)	−7.24	4.14×10^{-9}	Level 5
(−)	(−) (m/z=475.83582)	−6.43	2.32×10^{-8}	Level 5
(−)	(−) (m/z=668.76323)	−7.81	1.86×10^{-9}	Level 5
(−)	(−) (m/z=546.46825)	−9.17	5.46×10^{-10}	Level 5
(−)	(−) (m/z=798.72096)	−9.26	5.50×10^{-10}	Level 5
(−)	(−) (m/z=492.78803)	−3.29	9.19×10^{-10}	Level 5
(−)	(−) (m/z=479.82963)	−8.57	5.45×10^{-10}	Level 5
(−)	(−) (m/z=737.74217)	−11.3	5.45×10^{-10}	Level 5
(−)	(−) (m/z=485.83283)	−8.38	5.45×10^{-10}	Level 5
(−)	(−) (m/z=483.33303)	−8.93	5.81×10^{-10}	Level 5
(−)	(−) (m/z=436.50843)	−7.72	6.65×10^{-10}	Level 5
(−)	(−) (m/z=442.51392)	−8.01	5.96×10^{-10}	Level 5
(−)	(−) (m/z=734.40977)	−8.42	5.45×10^{-10}	Level 5
(−)	(−) (m/z=509.78710)	−8.83	5.59×10^{-10}	Level 5
(−)	(−) (m/z=566.80270)	−8.34	5.45×10^{-10}	Level 5

(−) = No name/No formula described in CD

Table S7. Compounds more probably present in urban areas (Urban−Rural).

Compound	Formula	Log2 Fold Change	*p*−value	Identified level
Cocaine	$C_{17}H_{21}NO_4$	4.68	6.15×10^{-10}	Level 1
Azoxystrobin	$C_{22}H_{17}N_3O_5$	8.02	5.43×10^{-10}	Level 1
Cotinine	$C_{10}H_{12}N_2O$	4.20	5.40×10^{-10}	Level 1
Anhydroecgonine	$C_9H_{13}NO_2$	4.31	1.67×10^{-7}	Level 1
3−Methyladenine	$C_6H_7N_5$	6.46	5.45×10^{-5}	Level 1
3−Hydroxy−2−methylpyridine	C_6H_7NO	3.66	2.06×10^{-9}	Level 1
Imidacloprid	$C_9H_{10}ClN_5O_2$	6.57	5.47×10^{-10}	Level 1
Metalaxyl	$C_{15}H_{21}NO_4$	3.78	5.42×10^{-10}	Level 1
Nicotine	$C_{10}H_{14}N_2$	10.37	2.36×10^{-6}	Level 1
Ethoprophos	$C_8H_{19}O_2PS_2$	4.24	5.43×10^{-10}	Level 1
1,8−Diazabicyclo [5.4.0]undec−7−ene	$C_9H_{16}N_2$	4.01	2.73×10^{-9}	Level 1
Pyrimethanil	$C_{12}H_{13}N_3$	3.03	5.17×10^{-10}	Level 1
Nikethamide	$C_{10}H_{14}N_2O$	6.40	5.45×10^{-10}	Level 2 (ms2)
Melamine	$C_3H_6N_6$	4.64	4.08×10^{-5}	Level 2 (ms2)
Phenacetin	$C_{10}H_{13}NO_2$	4.62	1.19×10^{-9}	Level 2 (ms3)
3−(2,6−Dioxocyclohexyl)propanenitrile	$C_9H_{11}NO_2$	6.21	5.33×10^{-10}	Level 2 (ms3)
2−Hydroxyphenylalanine	$C_9H_{11}NO_3$	4.98	5.26×10^{-10}	Level 2 (ms3)
3−(3−pyridinyl)propanoic acid	$C_8H_9NO_2$	5.11	7.72×10^{-10}	Level 2 (ms2)
Fenuron	$C_9H_{12}N_2O$	5.12	5.45×10^{-10}	Level 2 (ms2)
1−propylimidazole	$C_6H_{10}N_2$	6.53	1.02×10^{-09}	Level 2 (ms2)
Hydroferulic acid	$C_{10}H_{12}O_4$	5.30	1.51×10^{-09}	Level 2 (ms2)
Dihydrothymine	$C_5H_8N_2O_2$	5.21	4.74×10^{-08}	Level 2 (ms2)
(4−Methyl−1H−imidazol−5−yl)methanol	$C_5H_8N_2O$	6.63	5.48×10^{-10}	Level 2 (ms2)
(Butylamino)acetonitrile	$C_6H_{12}N_2$	6.29	5.45×10^{-10}	Level 2 (ms2)
Benzocaine	$C_9H_{11}NO_2$	5.13	4.04×10^{-4}	Level 2 (ms2)
N−Methyloctan−1−amine	$C_9H_{21}N$	5.82	5.42×10^{-10}	Level 2 (ms2)
Ropivacaine	$C_{17}H_{26}N_2O$	6.26	5.46×10^{-10}	Level 2 (ms2)
Cardiopetalidine	$C_{21}H_{33}NO_4$	5.14	5.45×10^{-10}	Level 2 (ms3)
Pyroquilon	$C_{11}H_{11}NO$	3.35	5.41×10^{-10}	Level 3
Norfenefrine	$C_8H_{11}NO_2$	4.13	1.24×10^{-8}	Level 3
Ethyl 4−hydroxy−3−methoxyphenylacetate	$C_{11}H_{14}O_4$	3.74	4.02×10^{-6}	Level 3
Diaminotoluene	$C_7H_{10}N_2$	5.22	8.00×10^{-10}	Level 3
NP−019811	$C_6H_7NO_2$	4.02	5.45×10^{-10}	Level 3
3h−1,2,3−triazolo[4,5−c]pyridin−4−amine	$C_5H_5N_5$	3.85	5.44×10^{-10}	Level 3
6−Methylnicotinonitrile	$C_7H_6N_2$	3.80	5.46×10^{-10}	Level 3
5−acetyl−2,6−dimethyl−1,2,3,4−tetrahydropyridin−4−one	$C_9H_{13}NO_2$	3.29	6.25×10^{-10}	Level 3
2,2,2−Trifluoro−1−(1−naphthyl)ethanol	$C_{12}H_9F_3O$	4.39	8.95×10^{-6}	Level 3
Methyl piperonyl ketone	$C_{10}H_{10}O_3$	3.16	1.10×10^{-9}	Level 3

Compound	Formula	Log2 Fold Change	p−value	Identified level
Methyl [5−methoxy−2−nitro−4−(trifluoromethyl)phenyl]acetate	$C_{11}H_{10}F_3NO_5$	4.81	7.86×10^{-9}	Level 3
8−Hydroxyquinoline	C_9H_7NO	5.12	6.38×10^{-10}	Level 3
1−Naphthylisocyanate	$C_{11}H_7NO$	5.45	5.46×10^{-10}	Level 3
3−methyl−5−phenylpyridazine	$C_{11}H_{10}N_2$	3.93	5.58×10^{-10}	Level 3
Xanthone	$C_{13}H_8O_2$	3.09	5.45×10^{-10}	Level 3
2−(Diethylamino)ethanol	$C_6H_{15}NO$	3.50	5.45×10^{-10}	Level 3
Duloxetine	$C_{18}H_{19}NOS$	3.05	1.23×10^{-9}	Level 3
4−Hexyl−1H−pyrazole	$C_9H_{16}N_2$	3.76	1.38×10^{-9}	Level 3
2,7−Difluoro−4,5−dimethoxy−2'H,5'H−spiro[fluorene−9, 4'−imidazolidine]−2',5'−dione	$C_{17}H_{12}F_2N_2O_4$	6.21	3.98×10^{-9}	Level 3
(2Z)−2−(2,2,2−Trifluoro−1−hydroxyethylidene)cyclohept anone	$C_9H_{11}F_3O_2$	3.30	9.63×10^{-6}	Level 3
Mepivacaine	$C_{15}H_{22}N_2O$	6.25	5.45×10^{-10}	Level 3
3−(Octylamino)propanenitrile	$C_{11}H_{22}N_2$	3.42	5.45×10^{-10}	Level 3
Propylhexedrine	$C_{10}H_{21}N$	3.28	5.48×10^{-10}	Level 3
Detomidine	$C_{12}H_{14}N_2$	3.43	5.45×10^{-10}	Level 3
(−)−Ecgonine methyl ester	$C_{10}H_{17}NO_3$	5.58	6.25×10^{-10}	Level 3
Ethyl 5−formyl−2,4−dimethyl−1H−pyrrole−3−carboxylate	$C_{10}H_{13}NO_3$	4.79	9.57×10^{-10}	Level 3
2,2'−(1,2−Phenylene)bis(1,1,3,3−tetramethylguanidine)	$C_{16}H_{28}N_6$	3.20	2.02×10^{-5}	Level 3
119183	$C_7H_7N_3$	4.51	5.45×10^{-10}	Level 3
7−Amino−3,4−dihydro−2(1H)−quinoxalinone	C8 H9 N3 O	6.00	5.77×10^{-10}	Level 3
Epinephrine	$C_9H_{13}NO_3$	3.59	5.55×10^{-10}	Level 3
5−(Cyanomethyl)−1H−imidazole−4−carbonitrile	$C_6H_4N_4$	6.22	3.09×10^{-8}	Level 3
2−Methyl−2H−indazol−4−amine	$C_8H_9N_3$	6.25	5.45×10^{-10}	Level 3
Aminobenzodiazapine	$C_9H_{11}N_3O$	5.72	5.45×10^{-10}	Level 3
11−Aminoundecanoic acid	$C_{11}H_{23}NO_2$	5.95	5.45×10^{-10}	Level 3
ZV4	$C_5H_{11}NO$	3.30	2.14×10^{-4}	Level 3
6−anilino−2,4−dioxo−1,2,3,4−tetrahydropyrimidine−5−ca rbonitrile	$C_{11}H_8N_4O_2$	4.36	1.21×10^{-4}	Level 3
KK9000000	$C_4H_7F_3O_2$	3.52	6.24×10^{-10}	Level 3
Anabasine	$C_{10}H_{14}N_2$	3.98	1.40×10^{-4}	Level 3
Gramine	$C_{11}H_{14}N_2$	3.34	2.27×10^{-4}	Level 3
2−(5−Amino−1H−pyrazol−1−yl)ethanol	$C_5H_9N_3O$	6.74	5.57×10^{-10}	Level 3
Glutaric acid	$C_5H_8O_4$	5.45	7.65×10^{-10}	Level 3
p−Xylylenediamine	$C_8H_{12}N_2$	5.62	5.45×10^{-10}	Level 3
4−(2−Aminopropyl)−N,N−dimethylaniline	$C_{11}H_{18}N_2$	4.26	5.45×10^{-10}	Level 3
N,N−Diethyltryptamine	$C_{14}H_{20}N_2$	3.34	5.45×10^{-10}	Level 3

Compound	Formula	Log2 Fold Change	p−value	Identified level
Iprovalicarb	$C_{18}H_{28}N_2O_3$	6.08	8.27×10^{-10}	Level 3
1−(Cyclohexylmethyl)piperazine	$C_{11}H_{22}N_2$	3.34	5.45×10^{-10}	Level 3
6−Vinylnicotinonitrile	$C_8H_6N_2$	5.89	5.45×10^{-10}	Level 3
(3−aminobenzyl)diethylamine	$C_{11}H_{18}N_2$	3.56	5.45×10^{-10}	Level 3
1−deoxy−1−(2,4−difluorophenyl)−?−D−ribofuranose	$C_{11}H_{12}F_2O_4$	5.07	6.18×10^{-10}	Level 3
3−hydroxybenzylhydrazine	$C_7H_{10}N_2O$	5.25	5.46×10^{-10}	Level 3
Dodecanedinitrile	$C_{12}H_{20}N_2$	4.26	5.45×10^{-10}	Level 3
Serotonin	$C_{10}H_{12}N_2O$	6.93	5.45×10^{-10}	Level 3
L−Tyrosine	$C_9H_{11}NO_3$	3.38	5.45×10^{-10}	Level 3
Ethyl 4,4,4−trifluoro−3−hydroxy−3−methoxybutanoate	$C_7H_{11}F_3O_4$	3.39	5.45×10^{-10}	Level 3
4−Tolylurea	$C_8H_{10}N_2O$	4.77	6.71×10^{-10}	Level 3
3−Methoxytyramine	$C_9H_{13}NO_2$	4.14	5.45×10^{-10}	Level 3
4,4'−Bipyridine	$C_{10}H_8N_2$	3.44	5.45×10^{-10}	Level 3
2,2−Diethoxy−N,N−diethylethanamine	$C_{10}H_{23}NO_2$	3.46	5.45×10^{-10}	Level 3
2−Methoxy−5−methylaniline	$C_8H_{11}NO$	4.01	5.45×10^{-10}	Level 3
7−Aminoindole	$C_8H_8N_2$	4.57	1.01×10^{-9}	Level 3
2,2,6,6−Tetramethyl−4−piperidinol	$C_9H_{19}NO$	6.37	5.58×10^{-10}	Level 3
Pyrene	$C_{16}H_{10}$	9.58	1.94×10^{-4}	Level 3
NP−020713	$C_{20}H_{26}O_4$	3.03	5.47×10^{-10}	Level 3
3−[(4−Phenyl−2−butanyl)amino]propanenitrile	$C_{13}H_{18}N_2$	7.18	5.46×10^{-10}	Level 3
Skatole	C_9H_9N	3.11	5.45×10^{-10}	Level 3
Metrafenone	$C_{19}H_{21}BrO_5$	5.89	5.46×10^{-10}	Level 3
3−hydroxy−3−methylpentanedioic acid	$C_6H_{10}O_5$	3.14	1.24×10^{-7}	Level 3
1−Azaspiro[5.7]tridecane	$C_{12}H_{23}N$	4.51	5.45×10^{-10}	Level 3
2−deoxyglucose	$C_6H_{12}O_5$	3.53	2.71×10^{-9}	Level 3
4−hydroxy−3−(3−methylbut−2−en−1−yl)benzoic acid	$C_{12}H_{14}O_3$	3.63	1.51×10^{-9}	Level 3
2−aminododecanol	$C_{12}H_{27}NO$	3.82	5.45×10^{-10}	Level 3
7−hydroxy−6−methoxy−2H−chromen−2−one	$C_{10}H_8O_4$	5.99	5.45×10^{-10}	Level 3
6−(Diethylamino)−1−hexanol	$C_{10}H_{23}NO$	3.26	5.45×10^{-10}	Level 3
7beta,12alpha−Dihydroxykaurenolide	$C_{20}H_{28}O_4$	6.68	7.07×10^{-10}	Level 3
Paraxanthine	$C_7H_8N_4O_2$	4.30	5.72×10^{-10}	Level 3
(4−Methoxybenzyl)(phenyl)phosphine	$C_{14}H_{15}OP$	3.05	5.51×10^{-10}	Level 3
16−Heptadecyne−1,2,4−triol	$C_{17}H_{32}O_3$	3.01	5.45×10^{-10}	Level 3
MFCD00995440	$C_{10}H_{20}N_2$	3.68	5.45×10^{-10}	Level 3
3964	$C_7H_8N_2O$	4.33	5.45×10^{-10}	Level 3
Triethylamine	$C_6H_{15}N$	3.46	5.86×10^{-10}	Level 3
1,3−Phenylenediamine	$C_6H_8N_2$	3.73	5.45×10^{-10}	Level 3
6,7−Dihydro−8(5H)−quinolinone	C_9H_9NO	4.67	6.55×10^{-10}	Level 3

Compound	Formula	Log2 Fold Change	*p*–value	Identified level
4,6–Bis(1–aziridinyl)–N–(2,2–dimethyl–1,3–dioxan–5–yl)–1,3,5–triazin–2–amine	$C_{13}H_{20}N_6O_2$	4.45	7.98×10^{-10}	Level 3
NSC 131681	C_8H_9NO	6.08	5.46×10^{-10}	Level 3
5–Ethylcyclohexane–1,3–dione	$C_8H_{12}O_2$	6.60	8.16×10^{-10}	Level 3
3–CYCLOHEXYLAMINOPROPIONITRILE	$C_9H_{16}N_2$	3.26	5.45×10^{-10}	Level 3
CM3599500	$C_8H_{13}NO_2$	3.35	5.46×10^{-10}	Level 3
6–Methyl–2–pyridinemethanol	C_7H_9NO	3.06	3.47×10^{-4}	Level 3
Creatinine	$C_4H_7N_3O$	3.45	1.06×10^{-9}	Level 3
N–(4–fluorophenyl)–2–methyl–5–(morpholinosulfonyl)–3–furamide	$C_{16}H_{17}FN_2O_5S$	4.54	2.89×10^{-9}	Level 3
8–[4–(3,4,5–Trifluorophenyl)cyclohexyl]–1,4–dioxaspiro[4.5]decane	$C_{20}H_{25}F_3O_2$	6.56	4.23×10^{-9}	Level 3
N~5~–(Diaminomethylene)–L–ornithyl–L–valyl–L–alanine	$C_{14}H_{28}N_6O_4$	3.51	5.45×10^{-10}	Level 3
9–Amino–1–nonanol	$C_9H_{21}NO$	3.36	5.45×10^{-10}	Level 3
2,2–Dimethoxy–1–(1–piperidinyl)ethanone	$C_9H_{17}NO_3$	3.39	5.45×10^{-10}	Level 3
1,3–diazaspiro[4.4]nonane–2,4–dione|5,5–TETRAMETHYLENEHYDANTOIN	$C_7H_{10}N_2O_2$	3.11	5.07×10^{-09}	Level 3
2–dimethylaminomethyl–1–methylpyrrole	$C_8H_{14}N_2$	3.49	5.45×10^{-10}	Level 3
Ethyl 3–hydroxy–4,4,4–trifluorobutyrate	$C_6H_9F_3O_3$	3.15	1.52×10^{-5}	Level 3
1–propyl–1H–benzo[d]imidazole hydrobromide	$C_{10}H_{12}N_2$	3.56	6.53×10^{-7}	Level 3
Disperse Yellow 3	$C_{15}H_{15}N_3O_2$	3.90	5.89×10^{-8}	Level 3
3–Hydroxyanthranilic acid	$C_7H_7NO_3$	3.53	5.45×10^{-10}	Level 3
2,2,2–Trifluoro–N–(5–hexen–1–yl)–N–methylacetamide	$C_9H_{14}F_3NO$	3.47	5.45×10^{-10}	Level 3
1–Nitrosocyclohexyl trifluoroacetate	$C_8H_{10}F_3NO_3$	3.21	4.66×10^{-7}	Level 3
Andrographolide	$C_{20}H_{30}O_5$	3.86	5.49×10^{-10}	Level 3
(−)	$C_{18}H_{31}F_5O_3S$	3.44	5.43×10^{-10}	Level 4
(−)	$C_{14}H_{26}F_3N_6O_8PS$	6.09	6.55×10^{-8}	Level 4
(−)	$C_{12}H_9N_7O_4P_2$	5.09	1.15×10^{-8}	Level 4
(−)	$C_5H_{16}FN_4P_3$	4.70	5.47×10^{-10}	Level 4
(−)	$C_{21}H_{30}ClO_{10}P$	5.38	5.96×10^{-10}	Level 4
(−)	$C_{15}H_{26}FN_7O_4$	3.30	5.47×10^{-10}	Level 4
(−)	$C_7H_7N_2O_6P$	3.12	9.71×10^{-10}	Level 4
(−)	$C_6H_7F_3O_4P_2$	3.31	1.36×10^{-9}	Level 4
(−)	$C_{10}H_{16}F_3NO_5$	4.08	5.47×10^{-10}	Level 4
(−)	$C_{11}H_{15}F_5N_4OS$	5.42	8.56×10^{-10}	Level 4
(−)	$C_4H_4N_7P$	7.57	2.50×10^{-7}	Level 4
(−)	$C_{17}H_{32}FNO_5$	4.24	5.45×10^{-10}	Level 4
(−)	$C_4H_{10}F_4N_8O_3$	6.03	1.29×10^{-9}	Level 4
(−)	$C_9H_{13}F_3O_5$	4.49	8.31×10^{-10}	Level 4
(−)	$C_9H_7N_6O_3P$	5.51	5.45×10^{-10}	Level 4
(−)	$C_3H_4N_6O_3$	5.28	1.09×10^{-8}	Level 4
(−)	$C_4H_{12}F_2N_5OPS$	3.59	9.49×10^{-10}	Level 4
(−)	$C_5H_{12}O_{10}$	4.03	3.79×10^{-6}	Level 4
(−)	$C_{10}H_{22}F_2N_4OS_3$	4.48	1.15×10^{-9}	Level 4
(−)	$C_{17}H_{35}F_3N_4O_{12}P_2$	5.26	2.06×10^{-8}	Level 4
(−)	$C_6H_{13}F_2N_2O_5P$	5.13	3.04×10^{-5}	Level 4
(−)	$C_{21}H_{28}F_4N_2O_{13}$	3.71	5.78×10^{-10}	Level 4
(−)	$C_{14}H_{13}FS$	3.61	5.45×10^{-10}	Level 4

	(−)	$C_{28}H_{51}F_2N_2O_3P$	3.58	1.51×10^{-9}	Level 4
	(−)	$C_{12}H_{17}FN_4O_4S_2$	5.13	5.92×10^{-9}	Level 4
	(−)	$C_{15}H_{16}FN_2P_3S$	5.53	5.45×10^{-10}	Level 4
	(−)	$C_8H_{10}N_6O_6$	5.39	5.45×10^{-10}	Level 4
	(−)	$C_{17}H_{29}N_4O_4P$	4.20	1.46×10^{-7}	Level 4
	(−)	$C_4H_7FN_2O_5$	5.13	1.10×10^{-7}	Level 4
	(−)	$C_6H_{14}FN_3OS$	4.00	3.45×10^{-4}	Level 4
	(−)	$C_{13}H_{32}F_2N_6OS$	3.87	1.34×10^{-9}	Level 4
	(−)	$C_8H_{14}F_3NO_4$	3.32	5.45×10^{-10}	Level 4
	(−)	$C_{20}H_{20}F_4N_2O_{10}$	8.10	5.45×10^{-10}	Level 4
	(−)	$C_{16}H_{25}FO_3$	3.32	6.30×10^{-10}	Level 4
	(−)	$C_{12}H_{16}F_4NO_9P$	4.01	6.12×10^{-10}	Level 4
	(−)	$C_{19}H_{31}F_3N_4O$	3.19	3.77×10^{-9}	Level 4
	(−)	$C_8H_{15}FN_4O_9S$	7.10	5.45×10^{-10}	Level 4
	(−)	$C_{17}H_{34}N_6O_5$	3.39	3.87×10^{-7}	Level 4
	(−)	$C_8H_8N_8O_3S$	5.89	5.45×10^{-10}	Level 4
	(−)	$C_{20}H_{21}F_3O_3$	4.38	5.45×10^{-10}	Level 4

Table S8. Compounds more probably present in rural areas (Urban−Rural).

Compound	Formula	Log2 Fold Change	*p*−value	Identified level
Triisopropanolamine	$C_9H_{21}NO_3$	−6.87	2.80×10^{-5}	Level 1
N,N,N',N'−Tetramethyl−2,5−disulfanylhexanediamide	$C_{10}H_{20}N_2O_2S_2$	−5.15	5.76×10^{-10}	Level 2 (ms2)
NP−021797	$C_{12}H_{22}O_3$	−5.19	8.66×10^{-10}	Level 2 (ms2)
2,2'−(1,7−Dioxa−4,10−diazacyclododecane−4,10−diyl)diethanol	$C_{12}H_{26}N_2O_4$	−11.00	5.45×10^{-10}	Level 2 (ms2)
Quingestanol	$C_{25}H_{34}O_2$	−6.08	1.09×10^{-9}	Level 2 (ms3)
Prolinol	$C_5H_{11}NO$	−7.84	5.46×10^{-10}	Level 2 (ms2)
N−(2−Aminoethyl)−9−pentofuranosyl−9H−purin−6−amine	$C_{12}H_{18}N_6O_4$	−6.22	5.43×10^{-10}	Level 2 (ms3)
8−Amino−7−oxononanoic acid	$C_9H_{17}NO_3$	−7.81	1.32×10^{-5}	Level 2 (ms3)
1,2,3,4−Tetramethyl−1,3−cyclopentadiene	C_9H_{14}	−5.05	7.02×10^{-5}	Level 2 (ms2)
(2R,3R,4R,5S,6R)−2−(Hydroxymethyl)−6−propyl−3,4,5−piperidinetriol	$C_9H_{19}NO_4$	−9.18	5.5×10^{-10}	Level 2 (ms3)
Dicirenone	$C_{26}H_{36}O_5$	−6.38	1.44×10^{-9}	Level 2 (ms3)
Heptanophenone	$C_{13}H_{18}O$	−7.56	5.60×10^{-10}	Level 2 (ms3)
3−(2−Oxocyclododecyl)propanenitrile	$C_{15}H_{25}NO$	−13.51	5.46×10^{-10}	Level 2 (ms3)
2−(2−Aminoethyl)−3−oxotetradecanoic acid	$C_{16}H_{31}NO_3$	−5.33	5.45×10^{-10}	Level 2 (ms2)
2,2−Methylenebis(4−ethyl−6−tert−butylphenol)	$C_{25}H_{36}O_2$	−5.55	6.67×10^{-5}	Level 3
2−Thiophenetellurol	C_4H_4STe	−3.06	2.44×10^{-4}	Level 3
N−(9−Decen−1−yl)−2,2,2−trifluoroacetamide	$C_{12}H_{20}F_3NO$	−3.22	5.46×10^{-10}	Level 3
Di−1−tetradecen−1−yl hydrogen phosphate	$C_{28}H_{55}O_4P$	−4.00	7.50×10^{-5}	Level 3
7−Hydroxycoumarinyl−γ−linolenate	$C_{27}H_{34}O_4$	−7.23	5.45×10^{-10}	Level 3
(1E)−1−Nitro−1−tridecene	$C_{13}H_{25}NO_2$	−5.35	9.85×10^{-9}	Level 3
Sorbicillin	$C_{14}H_{16}O_3$	−3.25	5.91×10^{-10}	Level 3
5−[3−(ethoxycarbonyl)−4−piperidinoanilino]−3,3−dimethyl−5−oxopentanoic acid	$C_{21}H_{30}N_2O_5$	−5.68	2.81×10^{-7}	Level 3
NP−021050	$C_{30}H_{48}O_4$	−3.38	5.45×10^{-10}	Level 3
1−Aminocyclododecanecarboxylic acid	$C_{13}H_{25}NO_2$	−4.33	5.46×10^{-10}	Level 3
(2R,9R,10R,15S)−14−(furan−3−yl)−2,6,6,10,15−pentamethyl−5,13−dioxotetracyclo[8.7.0.0Â?,â ·.0ÂaÂa,Âaâ µ]heptadeca−3,11−dien−9−yl acetate	$C_{28}H_{34}O_5$	−7.22	5.45×10^{-10}	Level 3
1,3−di−o−Tolylguanidine	$C_{15}H_{17}N_3$	−5.10	5.45×10^{-10}	Level 3
Viloxazine	$C_{13}H_{19}NO_3$	−4.42	6.60×10^{-10}	Level 3
10−Hydroxycarbazepine	$C_{15}H_{14}N_2O_2$	−6.22	6.03×10^{-10}	Level 3
3−Amino−3−(3,4,5−trimethoxyphenyl)propanoic acid	$C_{12}H_{17}NO_5$	−3.03	5.62×10^{-10}	Level 3
Oxymatrine	$C_{15}H_{24}N_2O_2$	−4.28	5.45×10^{-10}	Level 3
NP−015980	$C_{14}H_{24}O_6$	−3.83	1.09×10^{-9}	Level 3
2−[(4−Fluorophenyl)imino]−3−(2−oxo−2−phenylethyl)−2,3−dihydro−4H−1,3−benzoxazin−4−one	$C_{22}H_{15}FN_2O_3$	−7.44	5.45×10^{-10}	Level 3
8−(Dimethylamino)guanosine	$C_{12}H_{18}N_6O_5$	−5.00	2.83×10^{-5}	Level 3
11−Oxoetiocholanolone	$C_{19}H_{28}O_3$	−3.52	5.45×10^{-10}	Level 3
16α−Hydroxydehydroepiandrosterone	$C_{19}H_{28}O_3$	−5.20	5.47×10^{-10}	Level 3
DL−Glutamine	$C_5H_{10}N_2O_3$	−4.26	5.45×10^{-10}	Level 3
Metolachlor morpholinone	$C_{14}H_{19}NO_2$	−5.10	5.45×10^{-10}	Level 3
2−(3−Phenylpropyl)tetrahydrofuran	$C_{13}H_{18}O$	−4.12	5.97×10^{-4}	Level 3

Compound	Formula	Log2 Fold Change	p−value	Identified level
4−(4−methoxyphenyl)−6−pyridin−4−yl−1,3,5−triazin−2(3 H)−one	$C_{15}H_{12}N_4O_2$	−5.53	5.45×10^{-10}	Level 3
MFCD11502269	$C_{16}H_{33}NO_2$	−3.08	8.33×10^{-10}	Level 3
Betaxolol	$C_{18}H_{29}NO_3$	−3.63	5.45×10^{-10}	Level 3
DL−Carnitine	$C_7H_{15}NO_3$	−4.34	5.45×10^{-10}	Level 3
4−(Cyclooctylamino)−4−oxobutanoic acid	$C_{12}H_{21}NO_3$	−3.30	8.19×10^{-10}	Level 3
Fingolimod	$C_{19}H_{33}NO_2$	−3.10	6.68×10^{-10}	Level 3
2,5−Bis(5−tert−butyl−benzoxazol−2−yl)thiophene	$C_{26}H_{26}N_2O_2S$	−4.71	2.18×10^{-6}	Level 3
UNII:W9EN9DLM98	$C_9H_{21}NO_3$	−8.38	1.89×10^{-4}	Level 3
N−Boc−2−amino−2−methyl−1−propanol	$C_9H_{19}NO_3$	−7.64	7.17×10^{-7}	Level 3
Diheptyl phthalate	$C_{22}H_{34}O_4$	−5.22	4.96×10^{-5}	Level 3
PB2275000	$C_4H_{10}O_3S$	−6.62	1.40×10^{-6}	Level 3
MFCD08144705	$C_7H_{15}NO_2$	−5.74	1.37×10^{-5}	Level 3
4−Hydroxy−1−butanesulfonic acid	$C_4H_{10}O_4S$	−5.00	2.65×10^{-4}	Level 3
Gabapentin	$C_9H_{17}NO_2$	−4.52	3.08×10^{-9}	Level 3
5−[5−hydroxy−3−(hydroxymethyl)pentyl]−8a−(hydroxy methyl)−5,6−dimethyl−3,4,4a,5,6,7,8,8a−octahydronapht halene−1−carboxylic acid	$C_{20}H_{34}O_5$	−3.41	5.45×10^{-10}	Level 3
Pramocaine	$C_{17}H_{27}NO_3$	−5.22	5.45×10^{-10}	Level 3
Diethyl 3−(trifluoromethyl)pentanedioate	$C_{10}H_{15}F_3O_4$	−3.06	5.69×10^{-10}	Level 3
Benzyl {(2S)−1−(methyl{[(2−methyl−2−propanyl)oxy]carbonyl} amino)−3−[(3R)−tetrahydro−2H−pyran−3−yl]−2−propan yl}carbamate	$C_{22}H_{34}N_2O_5$	−5.88	1.17×10^{-8}	Level 3
8−[4−(2−Hydroxyethyl)−1−piperazinyl]−1,3−dimethyl−3 ,7−dihydro−1H−purine−2,6−dione	$C_{13}H_{20}N_6O_3$	−3.57	2.37×10^{-5}	Level 3
3−(Methylsulfonyl)−1−propanol	$C_4H_{10}O_3S$	−6.95	2.79×10^{-7}	Level 3
[2−(hydroxymethyl)−5,5,8a−trimethyl−1,4,4a,5,6,7,8,8a−octahydronaphthalen−1−yl]methanol	$C_{15}H_{26}O_2$	−4.09	5.91×10^{-10}	Level 3
L−Pyroglutamic acid	$C_5H_7NO_3$	−3.99	5.64×10^{-10}	Level 3
2−Methyl−2−propanyl 4−(1−amino−2,2,2−trifluoroethyl)−1−piperidinecarboxyl ate	$C_{12}H_{21}F_3N_2O_2$	−3.27	5.46×10^{-10}	Level 3
Dropropizine	$C_{13}H_{20}N_2O_2$	−3.87	6.01×10^{-10}	Level 3
2,2,2−Trifluoro−N−octylacetamide	$C_{10}H_{18}F_3NO$	−3.19	5.45×10^{-10}	Level 3
NP−004917	$C_{15}H_{26}O_3$	−5.28	5.45×10^{-10}	Level 3
Anapheline	$C_{13}H_{24}N_2O$	−4.18	5.45×10^{-10}	Level 3
Dinotefuran−metabolite−UF	$C_7H_{14}N_2O_2$	−3.95	5.45×10^{-10}	Level 3
2−oxa−4−azatetracyclo[6.3.1.1~6,10~.0~1,5~]tridecan−3−one	$C_{11}H_{15}NO_2$	−3.18	5.63×10^{-10}	Level 3
2−morpholino−1−phenyl−1−ethanol	$C_{12}H_{17}NO_2$	−4.01	1.06×10^{-9}	Level 3
Tolmetin	$C_{15}H_{15}NO_3$	−4.98	5.45×10^{-10}	Level 3
N,N'−1,2−Ethanediylbis(3−sulfanylpropanamide)	$C_8H_{16}N_2O_2S_2$	−6.20	1.28×10^{-7}	Level 3
(3beta,16alpha)−3,16−Dihydroxy−13,28−epoxyoleanan−30−al	$C_{30}H_{48}O_4$	−3.47	5.45×10^{-10}	Level 3
N,N−Dibutyl−4−morpholinesulfonamide	$C_{12}H_{26}N_2O_3S$	−8.98	5.49×10^{-10}	Level 3
1−Aminocycloundecanecarboxylic acid	$C_{12}H_{23}NO_2$	−3.35	5.45×10^{-10}	Level 3
4−oxosebacic acid	$C_{10}H_{16}O_5$	−3.61	5.45×10^{-10}	Level 3

2−Methyl−5,8,11−trioxa−2−azatetradecan−13−ol	$C_{11}H_{25}NO_4$	−11.86	5.45×10^{-10}	Level 3
1−Aminocyclodecanecarboxylic acid	$C_{11}H_{21}NO_2$	−4.60	5.45×10^{-10}	Level 3
2,4−Bis−(octylmercapto)−6−(4−hydroxy−3,5−di−tert−butylanilino)−1,3,5−triazine	$C_{33}H_{56}N_4OS_2$	−6.42	5.58×10^{-5}	Level 3

Compound	Formula	Log2 Fold Change	*p*–value	Identified level
5–Methyl–4–sulfanyl–2–(2,4,4–trimethyl–2–pentanyl)phenol	$C_{15}H_{24}OS$	−9.97	2.85 × 10^{-7}	Level 3
S–Isobutyl ethyl(2–{[(isobutylsulfanyl)carbonyl]amino}ethyl)carbamothioate	$C_{14}H_{28}N_2O_2S_2$	−8.16	5.45 × 10^{-10}	Level 3
N–Decyl–2,2,2–trifluoroacetamide	$C_{12}H_{22}F_3NO$	−4.54	5.45 × 10^{-10}	Level 3
512173	$C_4H_2Cl_4O_3$	−5.22	6.19 × 10^{-10}	Level 3
N–(1–Hydroxy–2–butanyl)undecanamide	$C_{15}H_{31}NO_2$	−3.66	5.45 × 10^{-10}	Level 3
1–Trifluoroacetyl Piperidine	$C_7H_{10}F_3NO$	−7.17	1.84 × 10^{-4}	Level 3
N–Acetylvaline	$C_7H_{13}NO_3$	−5.06	2.45 × 10^{-5}	Level 3
Ethyl 2,4–dioxohexanoate	$C_8H_{12}O_4$	−3.98	5.45 × 10^{-10}	Level 3
NP–018660	$C_{13}H_{22}O_3$	−3.13	1.04 × 10^{-7}	Level 3
N–HYDROXYETHYL–N–METHYLCAPRAMIDE	$C_{13}H_{27}NO_2$	−3.55	5.45 × 10^{-10}	Level 3
UNII:FD6L8T043R	$C_{16}H_{26}N_2O_4$	−8.36	5.45 × 10^{-10}	Level 3
euscaphic acid	$C_{30}H_{48}O_5$	−3.71	5.45 × 10^{-10}	Level 3
5,5–dimethyl–3–morpholinocyclohex–2–en–1–one	$C_{12}H_{19}NO_2$	−4.28	5.45 × 10^{-10}	Level 3
Isoetharine	$C_{13}H_{21}NO_3$	−4.53	5.45 × 10^{-10}	Level 3
3,3'–(1,12–Dodecanediyl)bis(1,2,4–oxadiazol–5–amine)	$C_{16}H_{28}N_6O_2$	−5.77	3.32 × 10^{-7}	Level 3
Jervine	$C_{27}H_{39}NO_3$	−5.61	4.05 × 10^{-7}	Level 3
N–Cyclohexyl–4–(2,2,2–trifluoroethoxy)cyclohexanamine	$C_{14}H_{24}F_3NO$	−5.71	5.45 × 10^{-10}	Level 3
Aldicarb oxime	$C_5H_{11}NOS$	−3.56	6.64 × 10^{-10}	Level 3
UNII:W9EN9DLM98	$C_9H_{21}NO_3$	−9.06	1.77 × 10^{-7}	Level 3
Butyl 2,2,2–trifluoro–N–hydroxyethanimidothioate	$C_6H_{10}F_3NOS$	−5.94	1.34 × 10^{-9}	Level 3
1–(2–Amino–ethyl)–N–Boc–cyclohexylamine	$C_{13}H_{26}N_2O_2$	−4.26	5.45 × 10^{-10}	Level 3
4–Octylphenol	$C_{14}H_{22}O$	−4.64	4.42 × 10^{-6}	Level 3
6,7,8–trimethoxy–2H–chromen–2–one	$C_{12}H_{12}O_5$	−7.52	1.62 × 10^{-9}	Level 3
N–Hexadecyl–1,4–dihydroxy–2–naphthamide	$C_{27}H_{41}NO_3$	−4.25	8.41 × 10^{-5}	Level 3
Diethyl sulfate	$C_4H_{10}O_4S$	−8.85	1.40 × 10^{-9}	Level 3
2–Cyclohexylacetohydrazide	$C_8H_{16}N_2O$	−3.09	5.45 × 10^{-10}	Level 3
Mazindol	$C_{16}H_{13}ClN_2O$	−4.28	5.45 × 10^{-10}	Level 3
2–Methyl–N–[3–(trifluoromethyl)phenyl]propanamide	$C_{11}H_{12}F_3NO$	−5.92	5.51 × 10^{-10}	Level 3
3603307	$C_9H_{19}NO_3$	−8.82	2.93 × 10^{-8}	Level 3
N–(9–oxodecyl)acetamide	$C_{12}H_{23}NO_2$	−3.31	5.45 × 10^{-10}	Level 3
2–HYDROXYLAURYLDIMETHYL BETAINE	$C_{16}H_{33}NO_3$	−5.24	9.30 × 10^{-10}	Level 3
2–Methyl–2–propanyl {2–[2–(2–hydroxyethoxy)ethoxy]ethyl}carbamate	$C_{11}H_{23}NO_5$	−6.76	7.32 × 10^{-10}	Level 3
(−)	$C_{27}H_{51}F_3N_2O_3$	−5.11	5.45 × 10^{-10}	Level 4
(−)	$C_{10}H_{20}F_2N_6OS$	−3.49	5.47 × 10^{-10}	Level 4
(−)	$C_{13}H_{25}N_9O$	−6.58	5.75 × 10^{-10}	Level 4
(−)	$C_{14}H_{25}NO_4P_2$	−3.33	1.13 × 10^{-9}	Level 4
(−)	$C_{20}H_{35}F_3O_3$	−3.08	5.45 × 10^{-10}	Level 4
(−)	$C_8H_{19}FN_8$	−4.13	5.45 × 10^{-10}	Level 4
(−)	$C_9H_{23}F_3N_6O$	−7.58	5.45 × 10^{-10}	Level 4
(−)	$C_{14}H_{27}F_3N_2O_6S_2$	−7.23	5.50 × 10^{-10}	Level 4
(−)	$C_{12}H_{24}F_2N6S$	−3.18	6.51 × 10^{-10}	Level 4
(−)	$C_{18}H_{29}FS$	−3.14	5.55 × 10^{-10}	Level 4
(−)	$C_{15}H_{27}N_5O_3$	−3.94	1.34 × 10^{-9}	Level 4

(−)	$C_{21}H_{45}N_3O_2S_2$	−5.94	1.86×10^{-7}	Level 4

Compound	Formula	Log2 Fold Change	p–value	Identified level
(−)	$C_4H_2F_{11}O_4P_3S_2$	−7.60	9.22 × 10^{-8}	Level 4
(−)	$C_{16}H_{38}N_8O_2S$	−6.27	1.18 × 10^{-9}	Level 4
(−)	$C_4H_{10}FN_3O_4$	−5.50	9.45 × 10^{-10}	Level 4
(−)	$C_{18}H_{35}FN_2O_6S$	−6.64	8.32 × 10^{-10}	Level 4
(−)	$C_{20}H_{39}FN_2O_4$	−6.59	5.45 × 10^{-10}	Level 4
(−)	$C_7HF_4O_{16}P_3S$	−3.55	7.33 × 10^{-4}	Level 4
(−)	$C_9H_{16}F_2N_2O_4S_2$	−3.26	5.47 × 10^{-10}	Level 4
(−)	$C_{16}H_{28}F_4NP$	−4.09	6.51 × 10^{-10}	Level 4
(−)	$C_9H_{24}N_5O_3P$	−6.61	5.45 × 10^{-10}	Level 4
(−)	$C_{12}H_{24}F_4N_4O_2$	−3.45	7.83 × 10^{-10}	Level 4
(−)	$C_{26}H_{36}O_4S_2$	−4.27	6.52 × 10^{-10}	Level 4
(−)	$C_{31}H_{48}N_4S_2$	−3.63	3.69 × 10^{-09}	Level 4
(−)	$C_{12}H_{25}ClN_4O_8$	−6.38	5.45 × 10^{-10}	Level 4
(−)	$C_{15}H_{28}F_3N$	−3.60	5.45 × 10^{-10}	Level 4
(−)	$C_{13}H_{31}FN_8OS_2$	−5.94	2.04 × 10^{-08}	Level 4
(−)	$C_{11}H_{28}N_4O_2P_2S$	−9.28	5.45 × 10^{-10}	Level 4
(−)	$C_3H_4N_6O_2S_2$	−7.26	5.45 × 10^{-10}	Level 4
(−)	$C_{14}H_{21}F_3O_5$	−6.09	2.53 × 10^{-7}	Level 4
(−)	$C_{13}H_{24}F_3NO$	−3.32	5.45 × 10^{-10}	Level 4
(−)	$C_{17}H_{30}FNO$	−8.13	2.09 × 10^{-9}	Level 4
(−)	$C_{14}H_{21}F_3O_4$	−6.89	5.45 × 10^{-10}	Level 4
(−)	$C_{10}H_{17}F_3N_2S_2$	−5.39	5.45 × 10^{-10}	Level 4
(−)	$C_{14}H_{21}F_3$	−3.93	5.45 × 10^{-10}	Level 4
(−)	$C_{18}H_{36}N_2O_2S_2$	−5.34	3.00 × 10^{-8}	Level 4
(−)	$C_5HF_2O_2P_3S$	−5.22	9.54 × 10^{-10}	Level 4
(−)	$C_{14}H_{29}FO_3$	−3.56	5.45 × 10^{-10}	Level 4
(−)	$C_{10}H_{20}F_2N_6O_2S$	−4.77	2.25 × 10^{-8}	Level 4
(−)	$C_{12}H_{21}F_3N_2S_2$	−7.81	5.49 × 10^{-10}	Level 4
(−)	$C_6H_9F_3N_2S_2$	−5.70	5.45 × 10^{-10}	Level 4
(−)	$C_9H_{19}FN_3O_4P$	−5.03	1.53 × 10^{-9}	Level 4
(−)	$C_{10}H_{17}F_3N_2O_2S_3$	−7.81	5.45 × 10^{-10}	Level 4
(−)	$C_{13}H_{22}F_3NO$	−5.43	3.87 × 10^{-9}	Level 4
(−)	$C_{17}H_{37}N_3O_2S_2$	−6.03	5.45 × 10^{-10}	Level 4
(−)	$C_{10}H_{20}F_2N_3OP$	−4.74	5.45 × 10^{-10}	Level 4
(−)	$C_{13}H_{29}NO_5$	−6.94	2.69 × 10^{-9}	Level 4
(−)	$C_{10}H_9F_5N_2O_3$	−4.19	5.45 × 10^{-10}	Level 4
(−)	$C_{19}H_{34}F_3NO$	−4.32	5.45 × 10^{-10}	Level 4
(−)	(−) (m/z=609.80086)	−6.51	1.29 × 10^{-5}	Level 5
(−)	(−) (m/z=541.81345)	−3.58	8.61 × 10^{-4}	Level 5
(−)	(−)(m/z=671.77315)	−6.79	5.31 × 10^{-9}	Level 5

(−) = No name/No formula described in CD

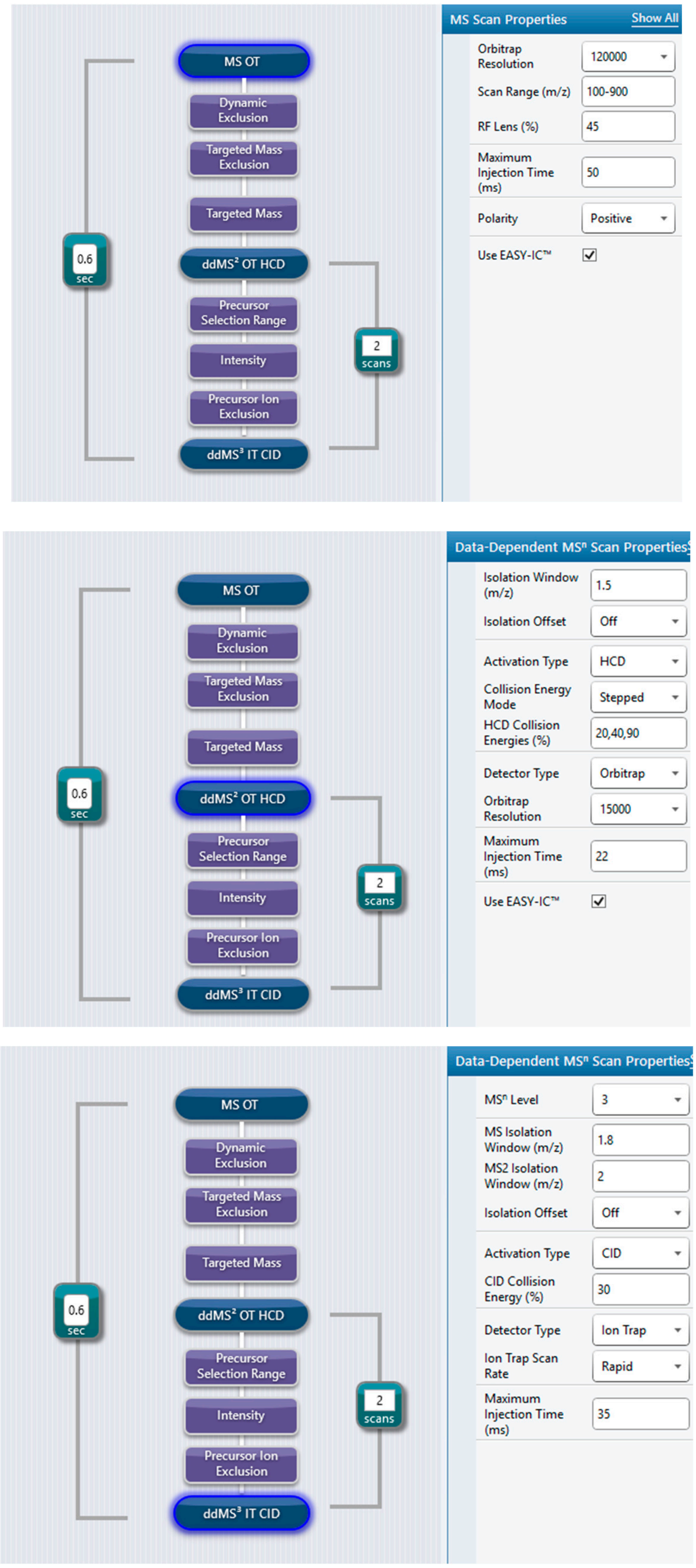

Figure S1. Workflow used in AcquireX Deep Scan Mode–MS^3.

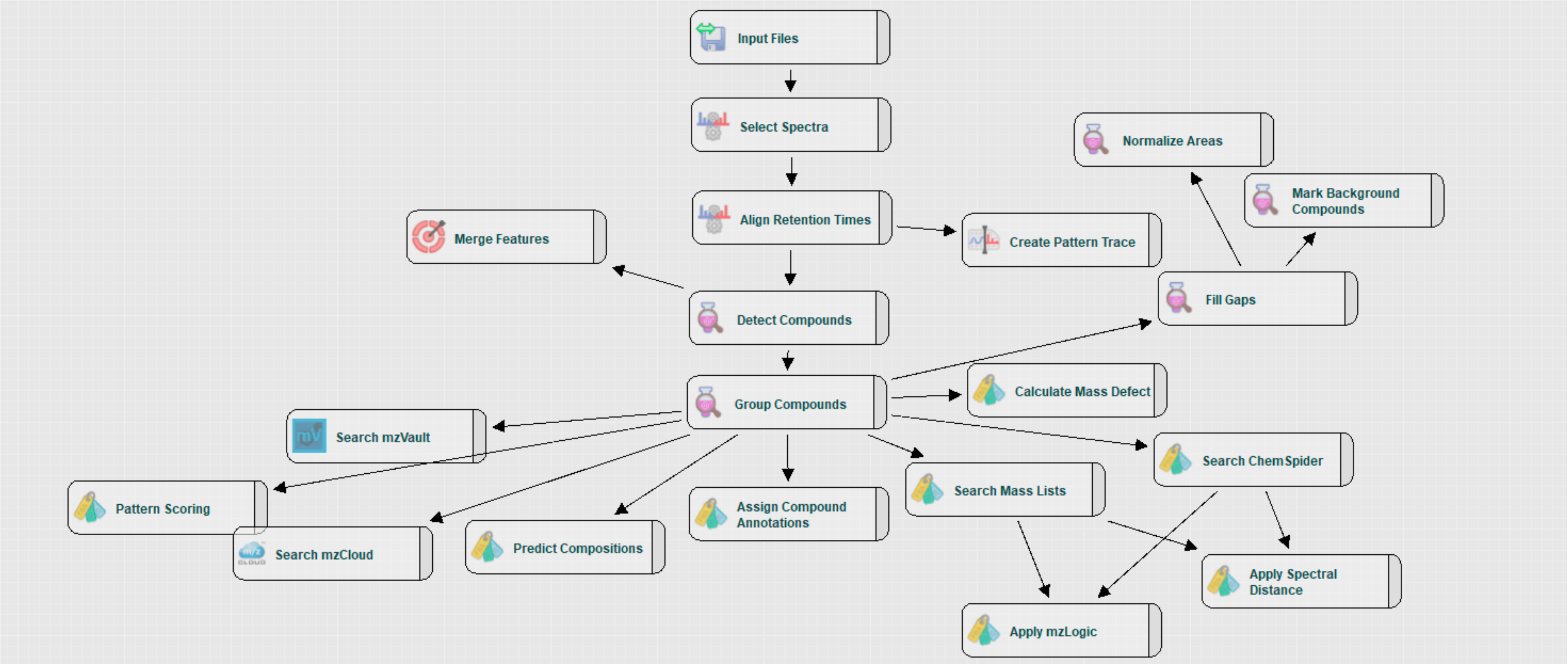

Figure S2. Workflow used in Compound Discoverer™ (CD) data processing.

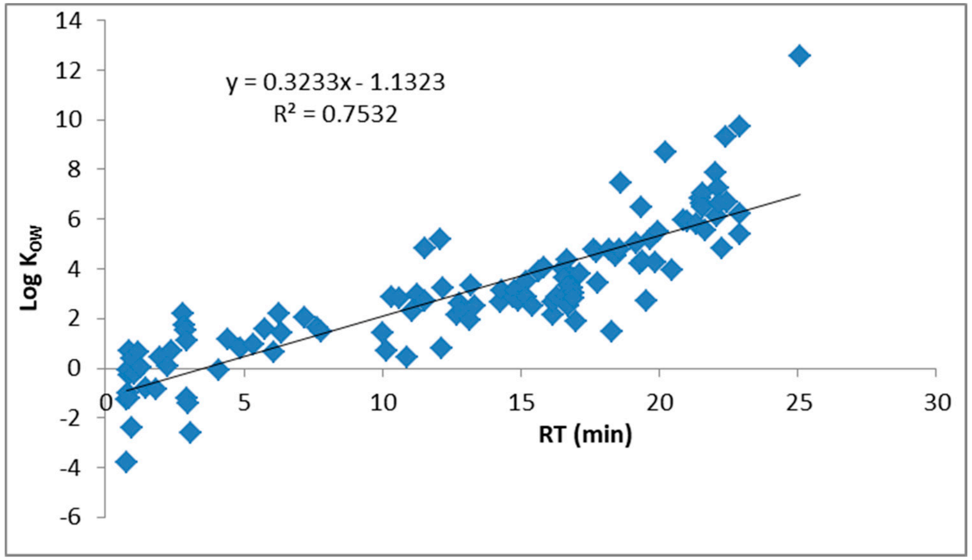

Figure S3. Graphic of Log K_{OW} vs Retention time (RT) of the analytical reference standards.

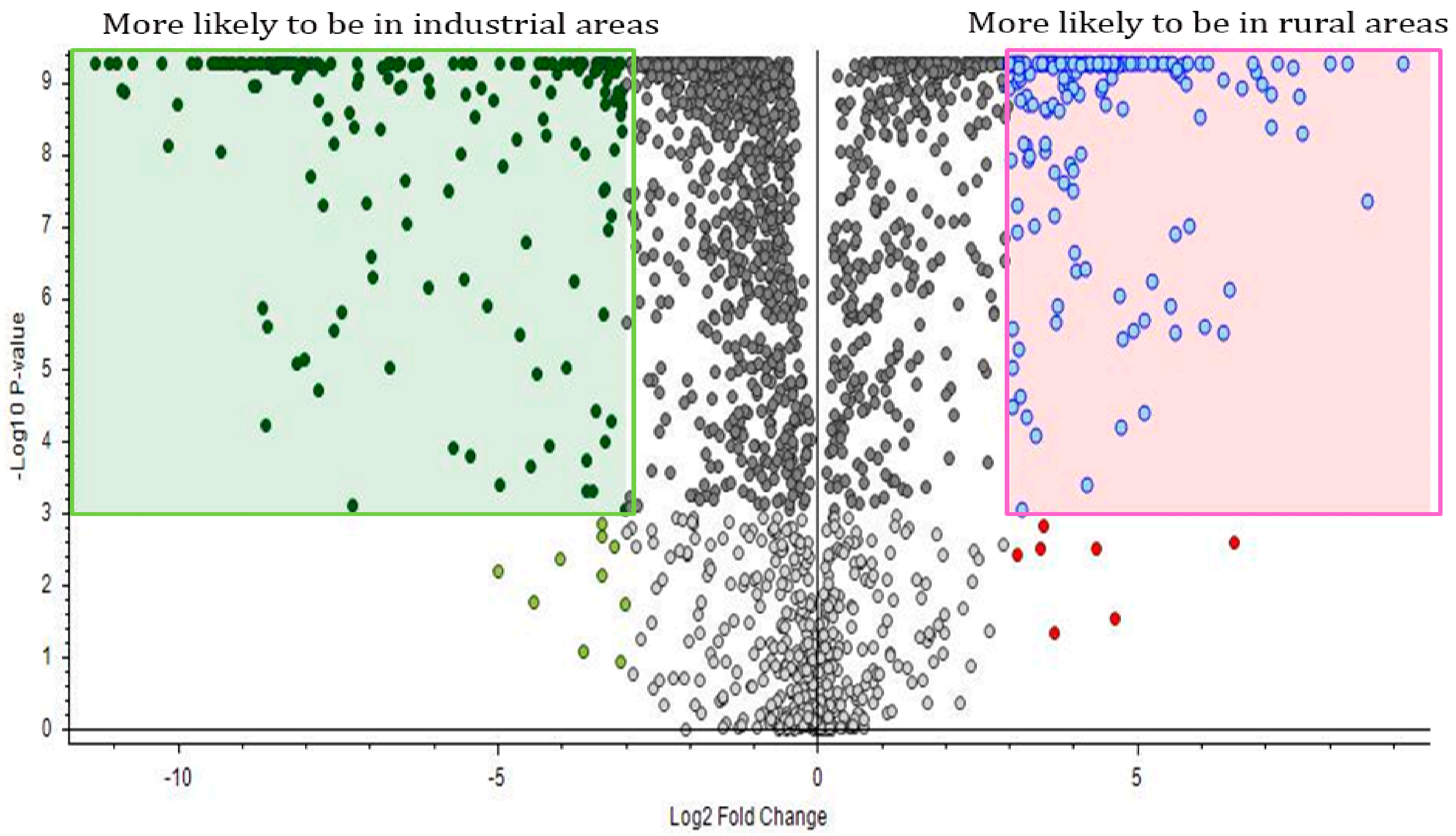

Figure S4. Volcano plot for identification of discriminating substances between industrial and rural areas (employed parameters: p−value=0.001 and log2Fold=3).

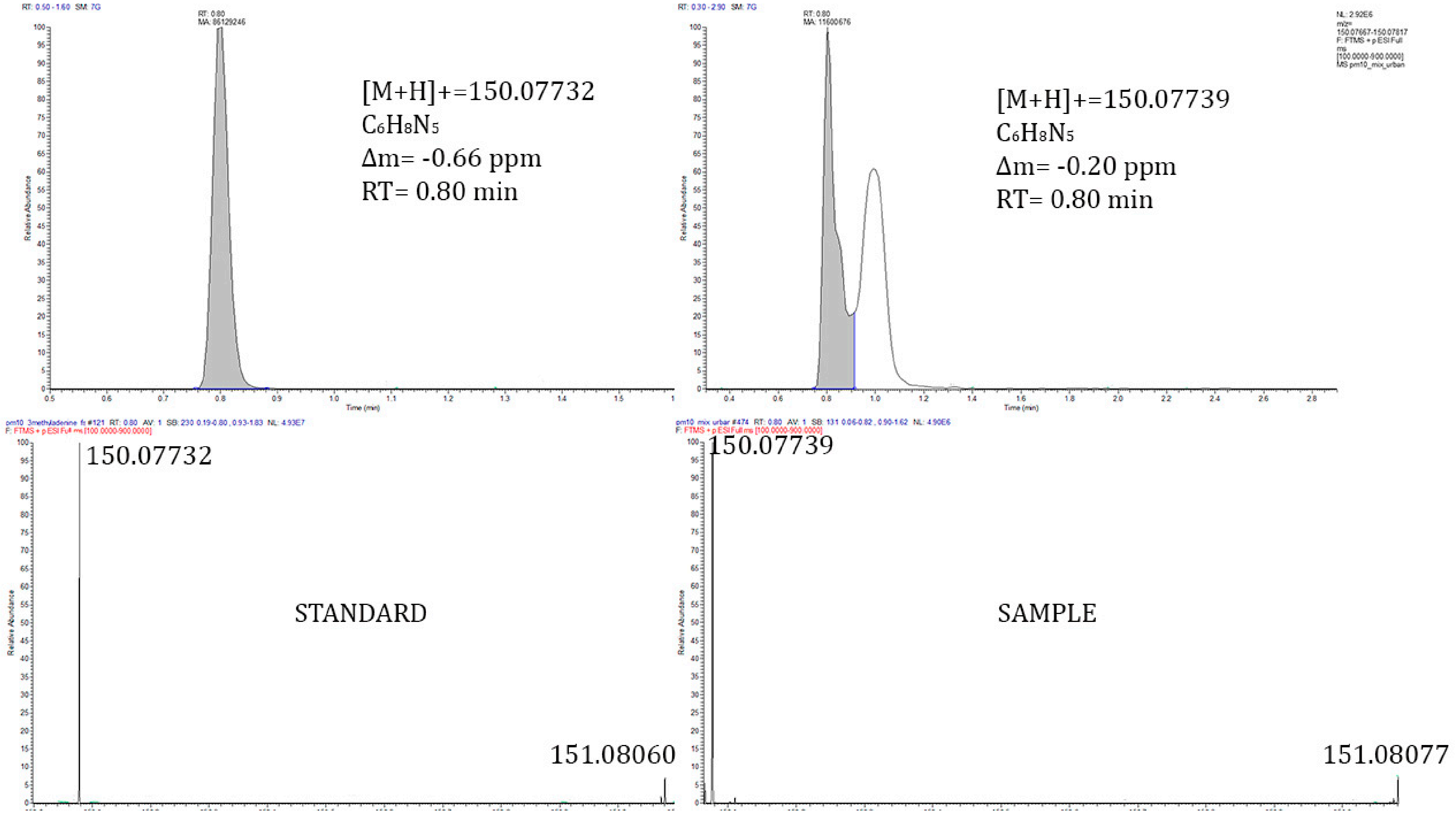

Figure S5. Extracted ion chromatogram (top) and isotopic profile (bottom) corresponding to the protonated molecule of 3−methyladenine in a standard (left) and a real sample (right).

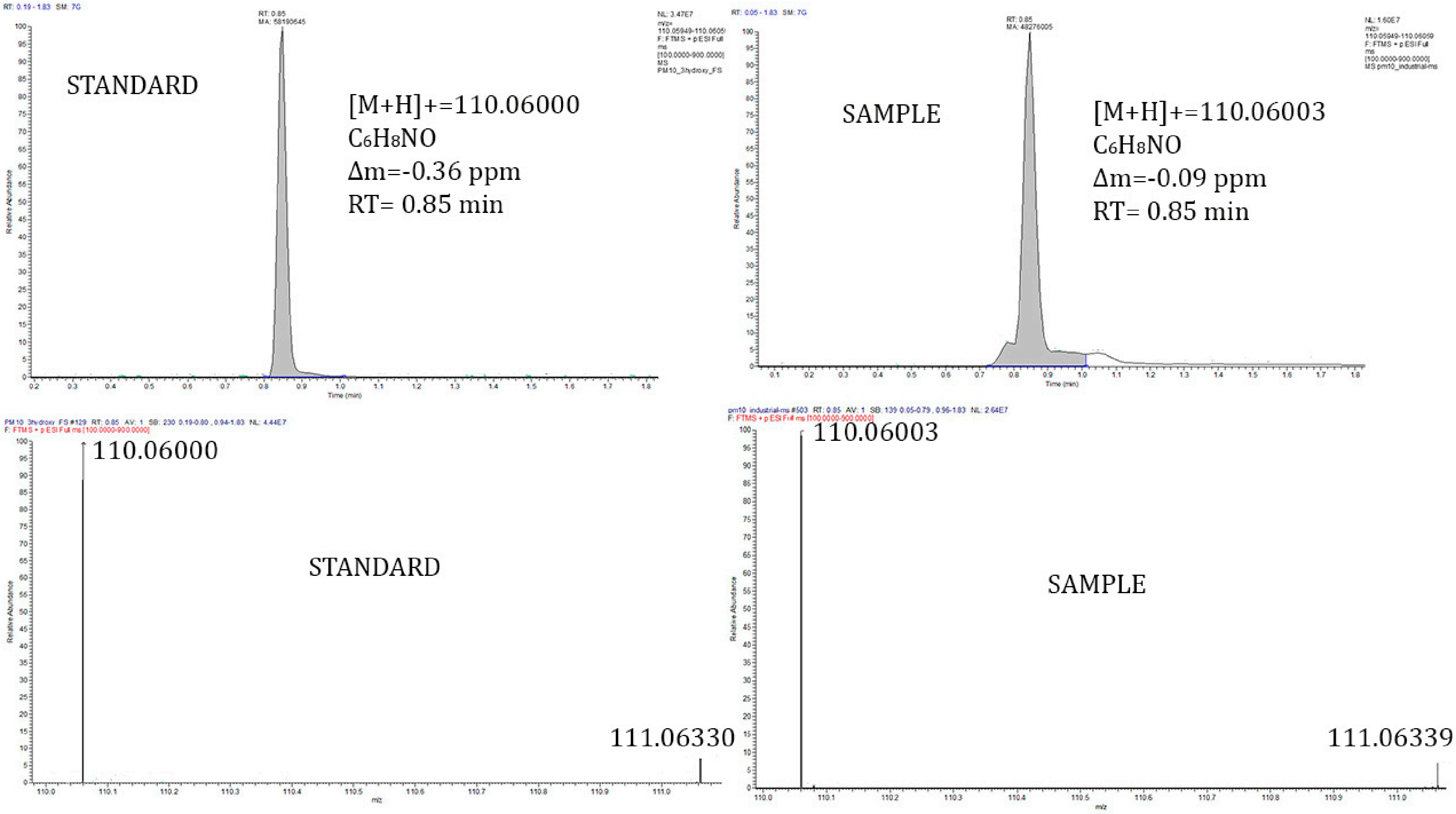

Figure S6. Extracted ion chromatogram (top) and isotopic profile (bottom) corresponding to the protonated molecule of 3–hydroxy–2–methylpyridine in a standard (left) and a real sample (right).

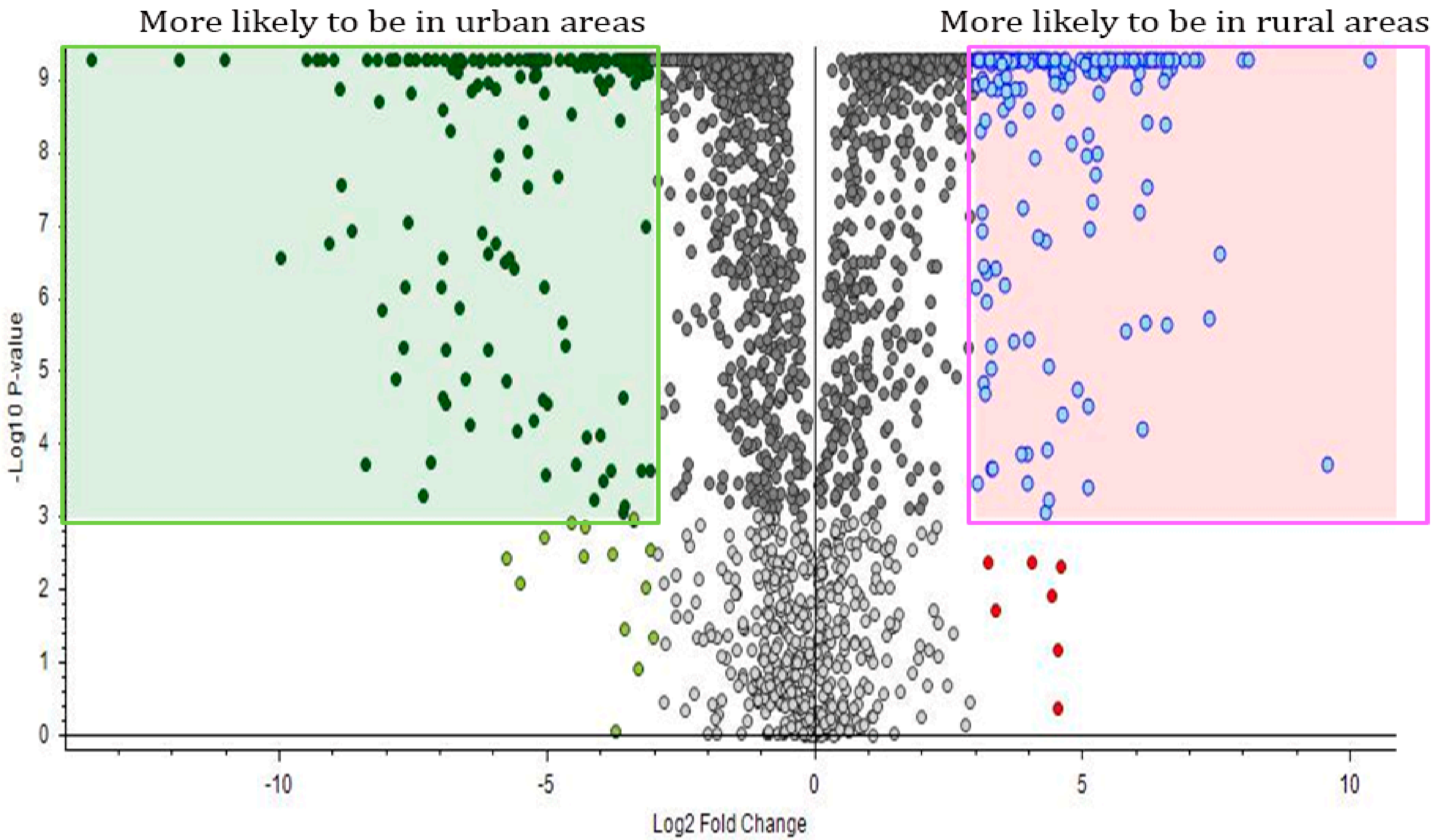

Figure S7. Volcano plot for identification of discriminating substances between urban and rural areas (employed parameters: p–value=0.001 and log2Fold=3).

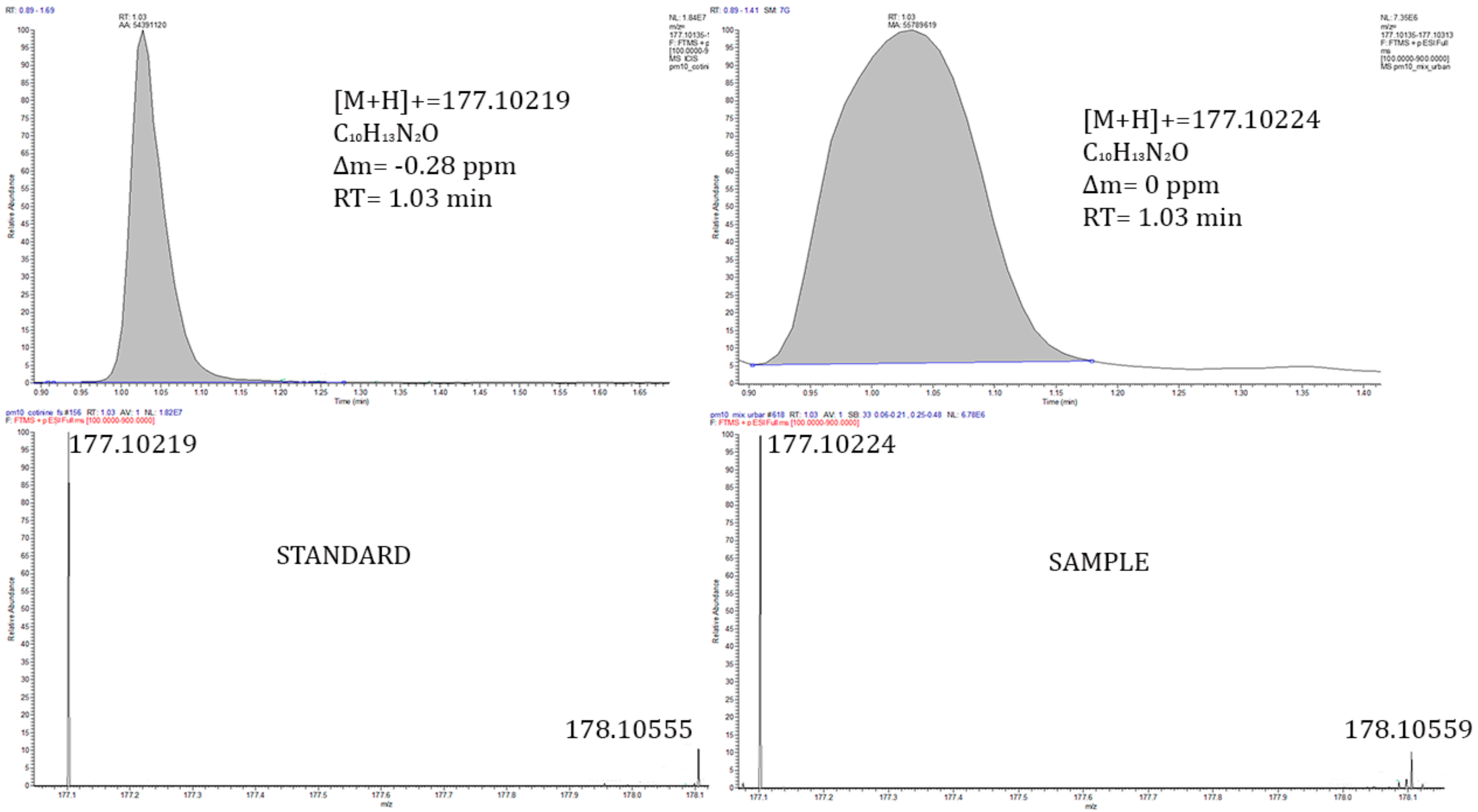

Figure S8. Extracted ion chromatogram (top) and isotopic profile (bottom) corresponding to the protonated molecule of cotinine in a standard (left) and a real sample (right).

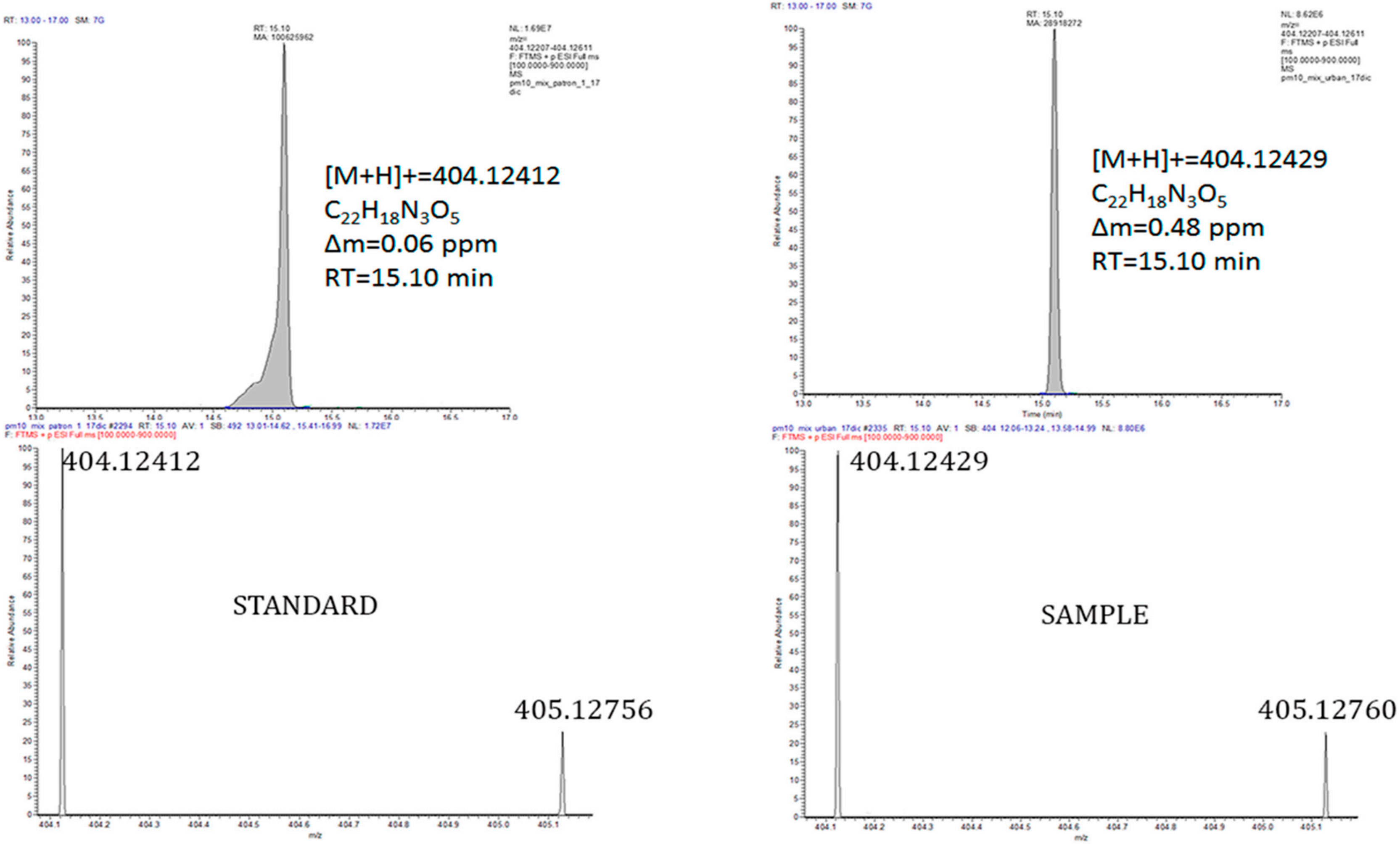

Figure S9. Extracted ion chromatogram (top) and isotopic profile (bottom) corresponding to the protonated molecule of azoxystrobin in a standard (left) and a real sample (right).

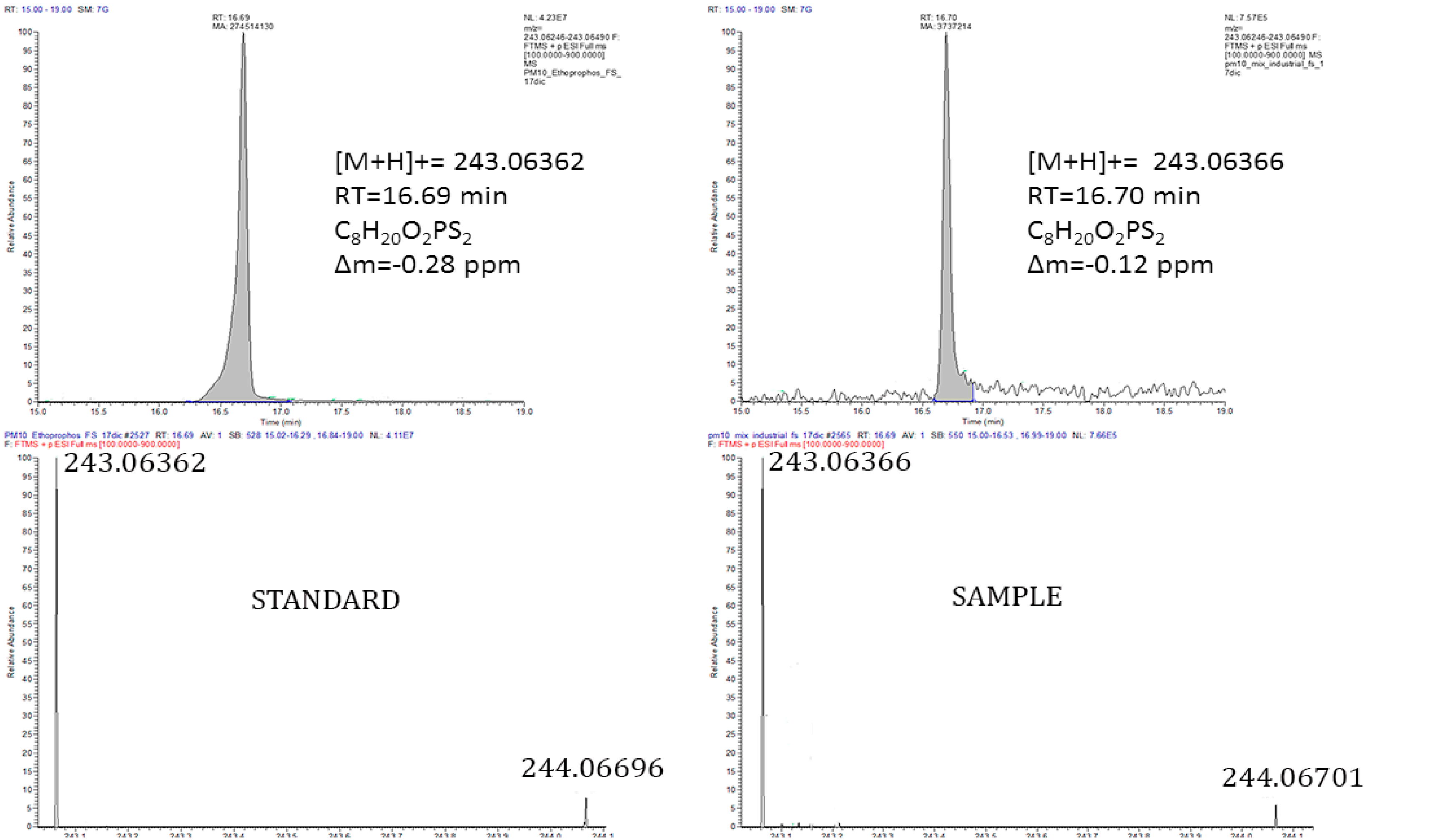

Figure S10. Extracted ion chromatogram (top) and isotopic profile (bottom) corresponding to the protonated molecule of ethoprophos in a standard (left) and a real sample (right).

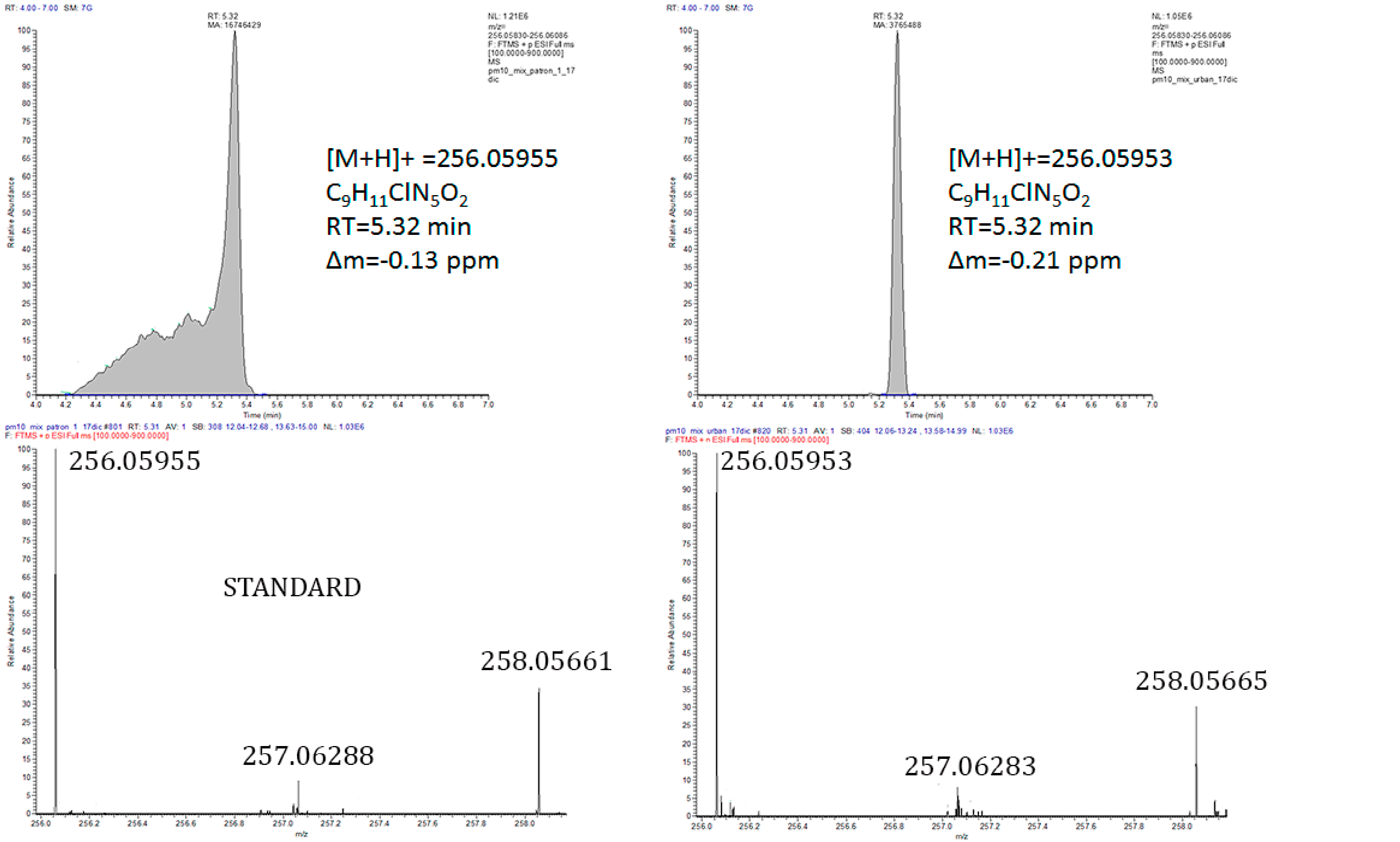

Figure S11. Extracted ion chromatogram (top) and isotopic profile (bottom) corresponding to the protonated molecule of imidacloprid in a standard (left) and a real sample (right).

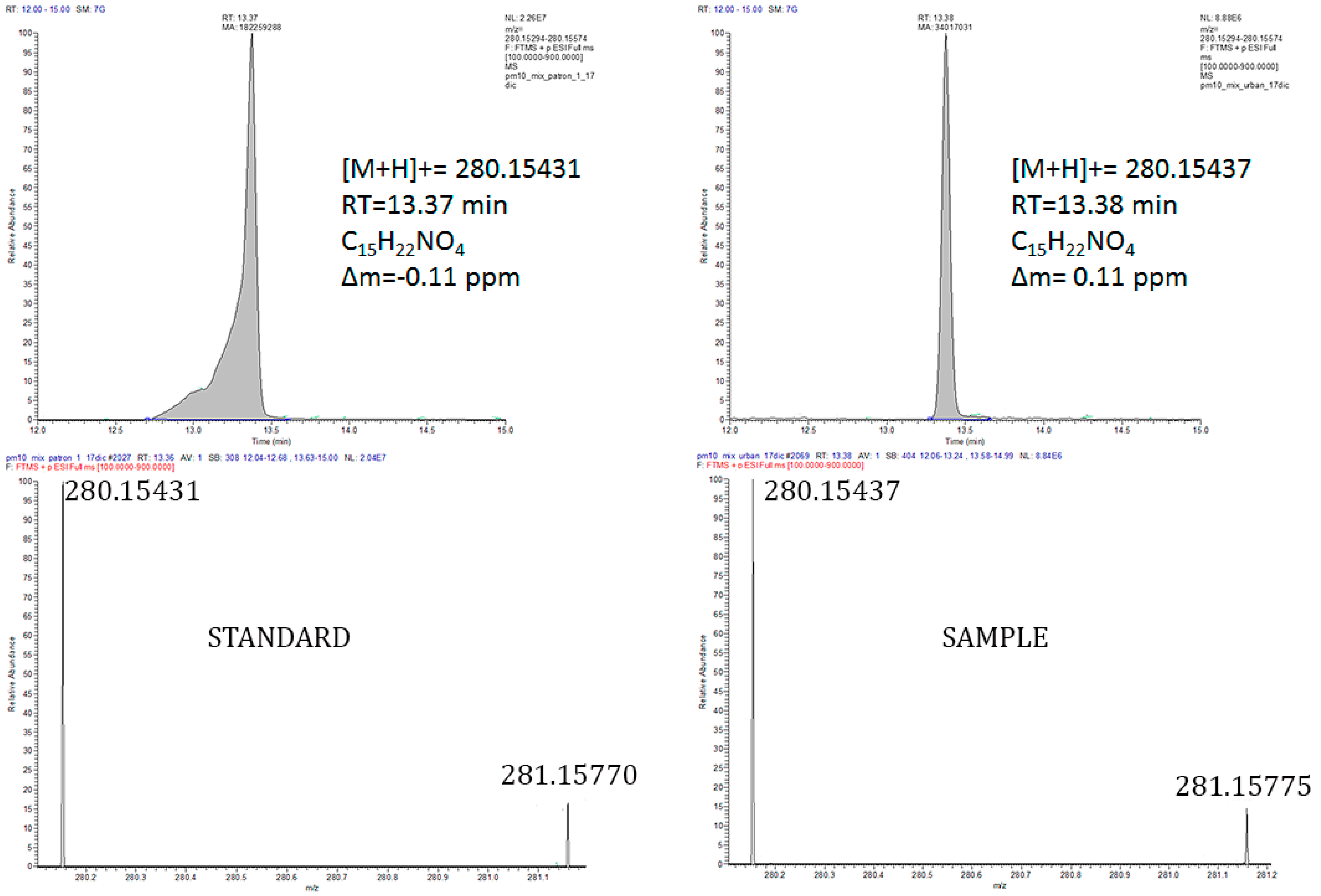

Figure S12. Extracted ion chromatogram (top) and isotopic profile (bottom) corresponding to the protonated molecule of metalaxyl in a standard (left) and a real sample (right).

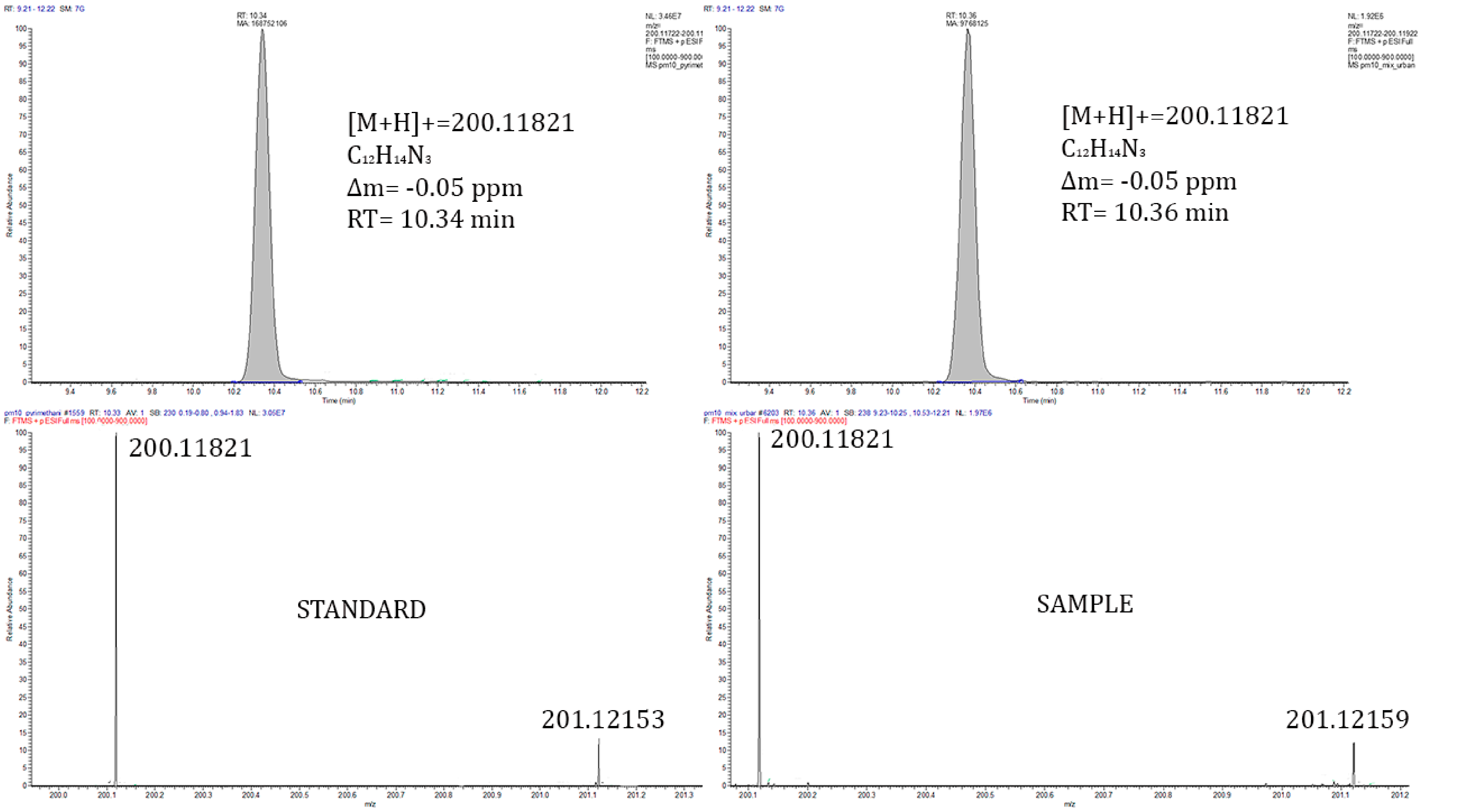

Figure S13. Extracted ion chromatogram (top) and isotopic profile (bottom) corresponding to the protonated molecule of pyrimethanil in a standard (left) and a real sample (right).

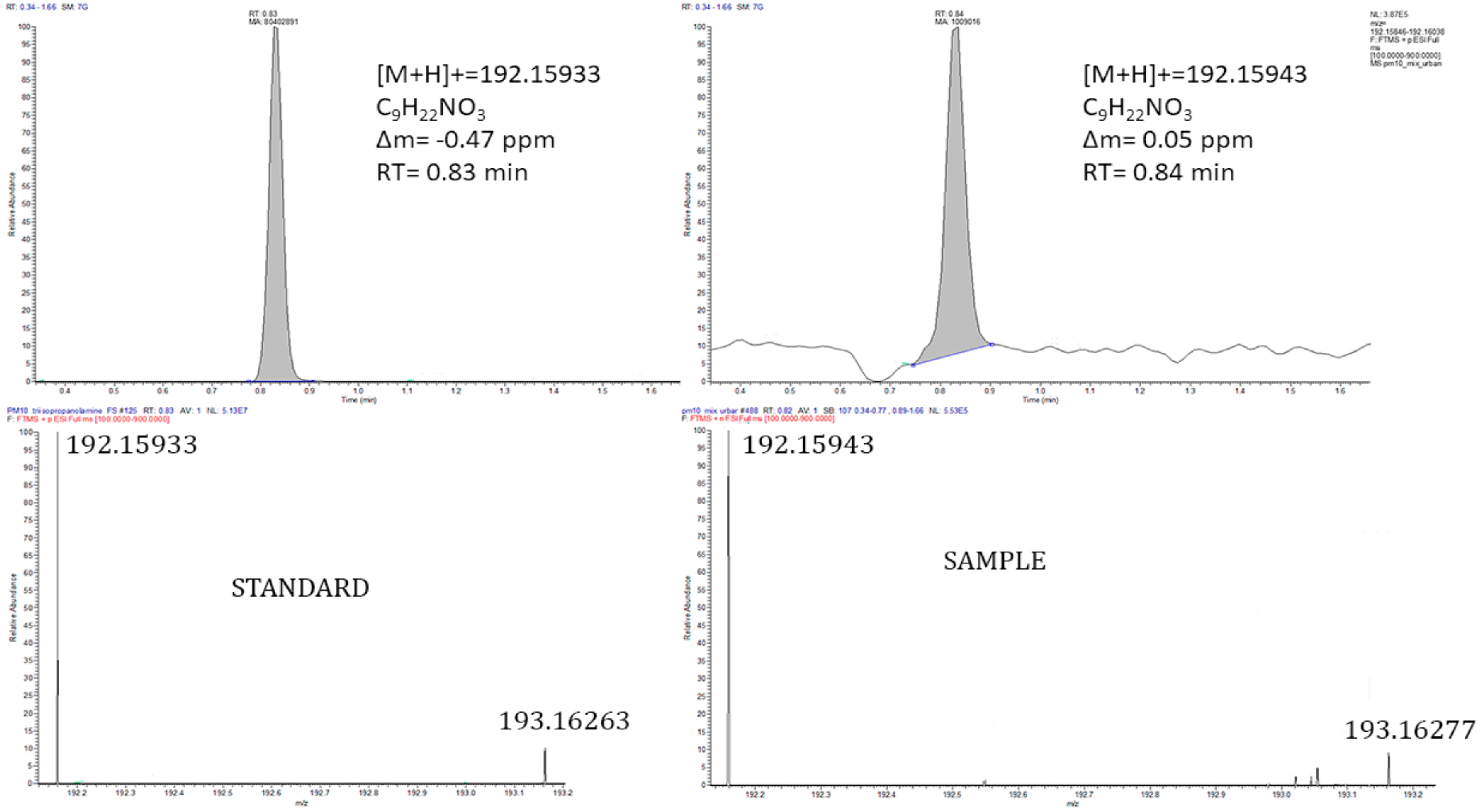

Figure S14. Extracted ion chromatogram (top) and isotopic profile (bottom) corresponding to the protonated molecule of triisopropanolamine in a standard (left) and a real sample (right)
